# Supplementary material for: Development and validation of multivariable clinical diagnostic models to identify type 1 diabetes requiring rapid insulin therapy in adults aged 18–50 years
Source: BMJ Open. 2019 Sep 26;9(9):e031586. doi: 10.1136/bmjopen-2019-031586 (PMC6773323; doi:10.1136/bmjopen-2019-031586)
Supplement: Supplementary data [file bmjopen-2019-031586supp001.pdf]

	DARE	PRIBA	MRC Pro/RetroMaster	MRC crossover
Included participants*	904	368	72	8
Data collection period	2007 to 2017	2011 to 2013	2013 to 2015	2013 to 2015
Study design	Cross-sectional	Longitudinal	Cross-sectional	Interventional Crossover
Setting	Primary and secondary care in eight diabetes research regions, England and retinal screening clinics.	Primary and secondary care in South West England	Primary and secondary care sites South West England, Tayside, Oxford, Glasgow, KCL and Newcastle, U.K.	Exeter and Tayside,U.K.
Inclusion criteria	Clinical diagnosis of diabetes (any type).	Clinical diagnosis of type 2 diabetes. Clinician determined requirement for DPP-IV inhibitor or GLP-1 analogue (HbA1c >7.5%)	Clinical diagnosis of type 2 diabetes non-insulin treated within 6 months of diagnosis. Participants were selected on the basis of rapid or slow progression to insulin therapy (<7, >7 years). Age 18-90 inclusive.	Clinical diagnosis of type 2 diabetes, currently treated with sulphonylurea tablets and no change in treatment in previous 3 months, Last HbA1c (within previous 12 months) ≥42 and ≤75 mmol/mol (6-9%). Age 19-79 inclusive.
Data collection	Clinical measurements and blood sample collected at visit. Ongoing biochemical data collected from pathology laboratories.	Clinical measurements and blood taken at initial visit. Follow up clinical measurements and blood collected at three and six months.	Clinical measures and fasting blood sample taken at visit.	MMT at baseline & MMT on each study drug visits. Three fasting blood collected at crossovers.

Supplementary Table 1: Cohort recruitment and data collection methods summary. *Included in the clinical features model stage 1 development.

Lynam A, *et al. BMJ Open* 2019; 9:e031586. doi: 10.1136/bmjopen-2019-031586

SNP	Gene	Odds Ratio	Weight	Effect Allele
rs2187668, rs7454108	DR3/DR4	48.18	3.87	
	DR3/DR3	21.12	3.05	
	DR4/DR4	21.98	3.09	
	DR4/X	7.03	1.95	
	DR3/X	4.53	1.51	
rs1264813	HLA_A_24	1.54	0.43	T
rs2395029	HLA_B_5701	2.5	0.92	T
rs3129889	HLA_DRB1_15	14.88	2.70	A
rs2476601	PTPN22	1.96	0.67	A
rs689	INS	1.75	0.56	T
rs12722495	IL2RA	1.58	0.46	T
rs2292239	ERBB3	1.35	0.30	T
rs10509540	C10orf59	1.33	0.29	T
rs4948088	COBL	1.3	0.26	C
rs7202877		1.28	0.25	G
rs12708716	CLEC16A	1.23	0.21	A
rs3087243	CTLA4	1.22	0.20	G
rs1893217	PTPN2	1.2	0.18	G
rs11594656	IL2RA	1.19	0.17	T
rs3024505	IL10	1.19	0.17	G
rs9388489	C6orf173	1.17	0.16	G
rs1465788		1.16	0.15	C
rs1990760	IFIH1	1.16	0.15	T
rs3825932	CTSH	1.16	0.15	C
rs425105		1.16	0.15	T
rs763361	CD226	1.16	0.15	T
rs4788084	IL27	1.16	0.15	C
rs17574546		1.14	0.13	C
rs11755527	BACH2	1.13	0.12	G
rs3788013	UBASH3A	1.13	0.12	A
rs2069762	IL2	1.12	0.11	A
rs2281808		1.11	0.10	C
rs5753037		1.1	0.10	T

Supplementary Table 2: Type 1 diabetes SNPs included in the genetic risk score with weights. Effect allele is the risk increasing allele on the positive strand.

Lynam A, *et al. BMJ Open* 2019; 9:e031586. doi: 10.1136/bmjopen-2019-031586

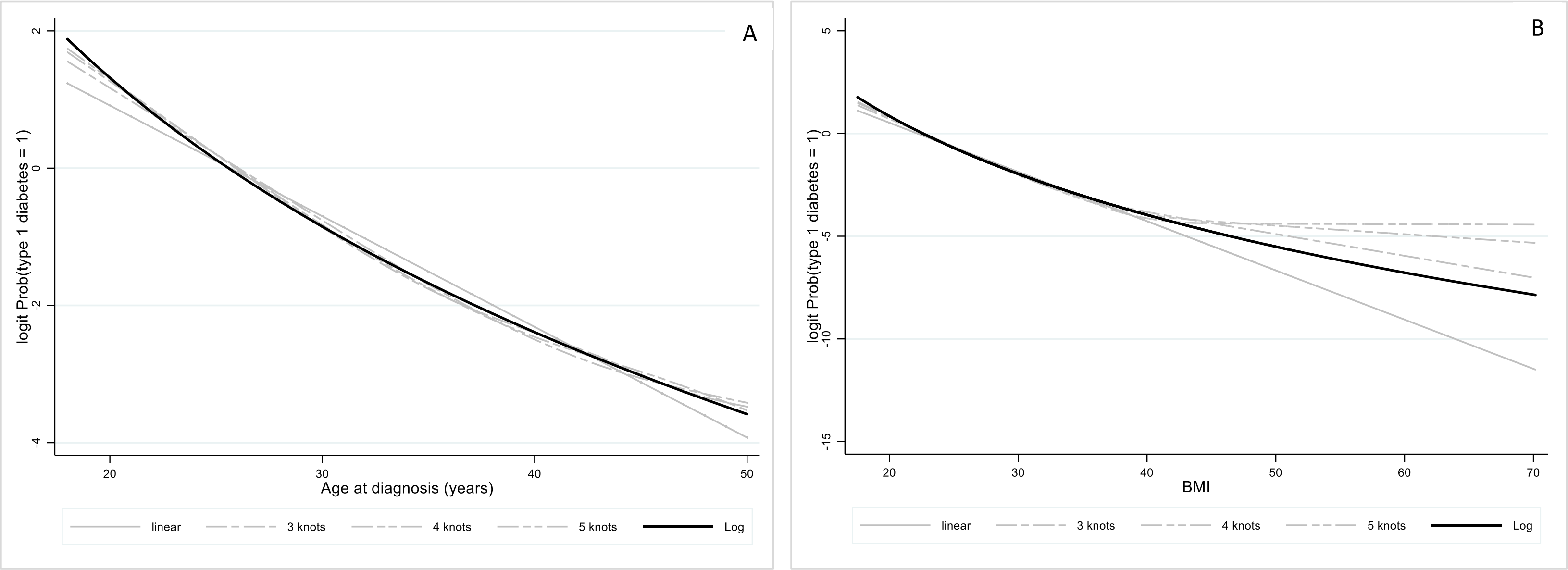

Supplementary Figure 1: Relationship between age at diagnosis (A) and BMI (B) and response modelled using restricted cubic splines (k = 3, 4 and 5) and a simple log transformation. Age at diagnosis and BMI did not predict linearly, the graphs of fitted splines and log transformation suggested that a simple log transformation was sufficient to induce linearity in both variables.

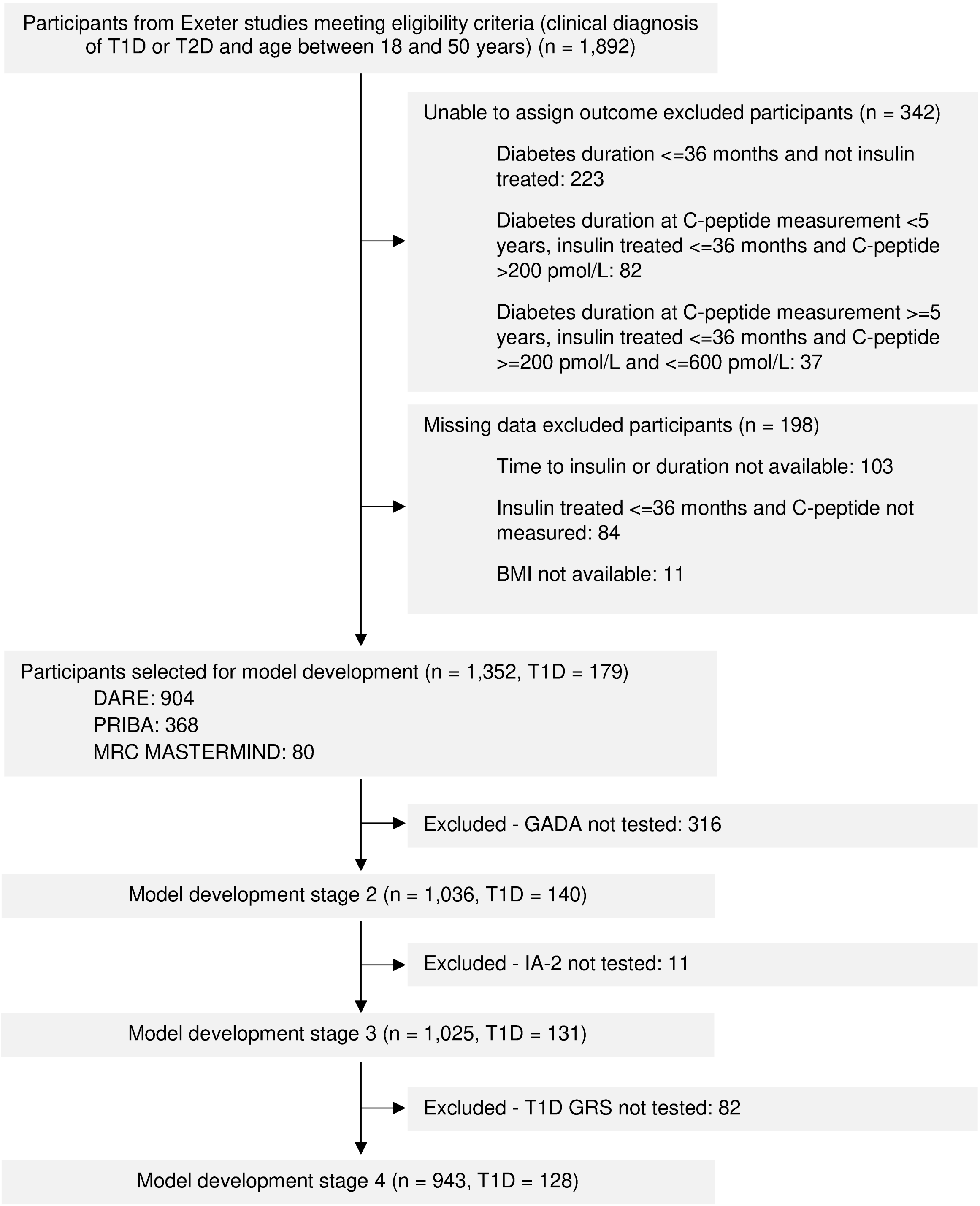

Supplementary Figure 2: Flow diagram of participants through the model development stages. T1D: type 1 diabetes, T2D: type 2 diabetes

Lynam A, *et al. BMJ Open* 2019; 9:e031586. doi: 10.1136/bmjopen-2019-031586

	Model 1 development n = 1,352	Model 2 development n = 1,036	Model 3 development n = 1,025	Model 4 development n = 943
**Characteristic**				
Sex (% Male)	59%	59%	59%	59%
Age at diagnosis (years)*	40 [39, 41]	40 [39, 40]	40 [39, 40]	40 [39, 40]
Age at diagnosis (years) min, max	18, 50	18, 50	18, 50	18, 50
BMI (kg/m^2)*†	33 [32, 33]	33 [32, 33]	33 [32, 33]	33 [32, 33]
BMI (kg/m^2)*† min, max	17.5, 70.2	17.5, 70.2	17.5, 70.2	17.5, 70.2
Duration of diabetes (years)	13 (8, 20)	13 (8, 20)	13 (8, 20)	13 (8, 20)
Type 1 diabetes	13%	14%	13%	14%
HbA1c (%)†	8.2 (7.1, 9.6)	8.3 (7.3, 9.8)	8.3 (7.3, 9.8)	8.2 (7.2, 9.7)
HbA1c (mmol/mol)†	66 (54, 81)	67 (56, 84)	67 (56, 84)	66 (55, 83)
GADA positive (%)	-	12%	12%	12%
IA-2 positive (%)	-	-	4%	4%
T1D GRS	-	-	-	0.24 (0.22, 0.26)
T1D GRS centile	-	-	-	5.8 (1.2, 23.7)
T1D GRS min, max	-	-	-	0.12, 0.32

Supplementary Table 3: Characteristics of the Exeter, U.K. study participants included at each model development stage. Model 1 – Clinical features (Age at diagnosis & BMI), Model 2 – Clinical features + GADA, Model 3 - Clinical features + GADA + IA-2, Model 4 - Clinical features + GADA + IA-2 + T1D GRS. Median (IQR) or % or *Geometric mean [95% CI] for transformed variables. †Measured at recruitment (median 13 years post diagnosis). Minimum and maximum values for each continuous predictor variable used in the models

Characteristic	Model 4 development n = 943	Model 4 development exclusions n = 409	p value for comparison
Sex (% Male)	59%	60%	>0.1
Age at diagnosis (years)*	40 [39, 40]	41 [40, 42]	0.04
BMI (kg/m^2)*†	33 [32, 33]	33 [32, 33]	> 0.1
Duration of diabetes (years)	13 (8, 20)	13 (7, 20)	> 0.1
Type 1 diabetes	14%	12%	> 0.1
HbA1c (%)†	8.2 (7.2, 9.7)	8.0 (6.9, 9.3)	0.009
HbA1c (mmol/mol)†	66 (55, 83)	64 (52, 78)	0.009

Supplementary Table 4: Comparison of characteristics for participants included in the model 4 development and participants included in model 1 development but excluded from model 4. Median (IQR) or % or *Geometric mean [95% CI] for transformed variables. †Measured at recruitment (median 13 years post diagnosis).

Performance parameter	Development sample validation	Internal validation (bootstrap 500) Apparent (SD)	test (SD)	Optimism
**Clinical features model (n = 1,352)**				
ROC [95% CI]	0.90 [0.88, 0.93]	0.9056 (0.013)	0.9038 (0.0005)	0.0018
Calibration-in-the-large	0	0.0000 (0.000)	0.0003 (0.1072)	-0.0003
Calibration slope (b_L)	1	1.0000 (0.000)	0.9977 (0.0678)	0.0023
Brier Score	0.07 (p = 0.50)	-	-	-
Hosmer-Lemeshow	p = 0.95	-	-	-
Jack-knife cross validation†	0.09	-	-	-
**Clinical features + GADA model (n = 1,036)**				
ROC [95% CI]	0.96 [0.95, 0.97]	0.9595 (0.0070)	0.9586 (0.0010)	0.0009
Calibration-in-the-large	0	0.0000 (0.0000)	-0.0019 (0.1472)	0.0019
Calibration slope (b_L)	1	1.0000 (0.0000)	0.9850 (0.0787)	0.015
Brier Score	0.05 (p = 0.35)	-	-	-
Hosmer-Lemeshow	p = 0.39	-	-	-
Jack-knife cross validation†	0.07	-	-	-
**Clinical features + GADA + IA-2 model (n = 1,025)**				
ROC [95% CI]	0.96 [0.95, 0.98]	0.9622 (0.007)	0.9633 (0.0015)	0.0011
Calibration-in-the-large	0	0.0000 (0.000)	0.0055 (0.1567)	-0.0055
Calibration slope (b_L)	1	1.0000 (0.000)	0.9780 (0.0707)	0.022
Brier Score	0.04 (p = 0.31)	-	-	-
Hosmer-Lemeshow	p = 0.14	-	-	-
Jack-knife cross validation †	0.06	-	-	-
**Clinical features + GADA + IA-2 + T1D GRS model (n = 943)**				
ROC [95% CI]	0.97 [0.96, 0.98]	0.9718 (0.0060)	0.9710 (0.0006)	0.0008
Calibration-in-the-large	0	0.0000 (0.0000)	0.0084 (0.1675)	-0.0084
Calibration slope (b_L)	1	1.0000 (0.0000)	0.9880 (0.0810)	0.0124
Brier Score	0.04 (p = 0.35)	-	-	-
Hosmer-Lemeshow	p = 0.84	-	-	-
Jack-knife cross validation †	0.06	-	-	-

Supplementary Table 5: Model performance results for the internal validation performed at each development stage. * P value for Brier score is Spiegelhalter's z-test used to evaluate the calibration component of the Brier score, significant p-values indicate poor calibration. †Result reported as raw cross-validation estimate of prediction error with misclassification cost function (cut-off 0.5). cv.glm function in R version 3.3.3.

Model	LR	Adequacy
Clinical features	324.7 (df 2)	0.67
Clinical features + GADA	418.7 (df 3)	0.87
Clinical features + GADA + IA-2	447.6 (df 5)	0.93
Clinical features + GADA + IA-2 + T1D GRS	481.8 (df 6)	1.00

Supplementary Table 6: Unitless index of adequacy is the proportion of log likelihood explained by each model stage with reference to the end model containing all predictors. Based on replica models developed using stage 4 development sample (n = 943).

Model comparison	Likelihood Ratio test	Net Reclassification Improvement	Integrated Discrimination Improvement
Adding GADA to Clinical features model	LR chi2(1) = 94.02 p <0.001	0.12, p = 0.01	0.13, p < 0.001
Adding IA-2 to Clinical features + GADA model	LR chi2 (2) = 28.82 p < 0.001	0.14, p = 0.004	0.15, p < 0.001
Adding T1D GRS to Clinical features + GADA + IA-2 model	LR chi2 (2) = 34.20 p < 0.001	0.06, p = 0.04	0.06, p < 0.001

Supplementary Table 7: Model fit comparisons of nested models developed using stage 4 development sample (n = 943). Null hypothesis for Likelihood Ratio test: Additional predictor(s) has no predictive information. Net Reclassification Improvement calculated using 50% classification cut-off.

Model	Clinical features ROC AUC	Clinical features + GADA ROC AUC	Clinical features + GADA + IA-2 ROC AUC
Development sample 1 (n = 1,352)	0.90 [0.88, 0.93]	-	-
Development sample 2 (n = 1,036)	-	0.96 [0.95, 0.97]	-
Development sample 3 (n = 1,025)	-	-	0.96 [0.95, 0.98]
**Development sample 4 (n = 943)**	**0.91 [0.89, 0.94]**	**0.96 [0.94, 0.97]**	**0.96 [0.95, 0.98]**

Supplementary Table 8: Model performance comparison with replica models developed using stage 4 development sample (n = 943).

Model	ROC AUC [95% CI]	n
Clinical Features	0.72 [0.61, 0.83]	104
Clinical Features + GADA	0.89 [0.80, 0.98]	78
Clinical Features + GADA + IA2	0.89 [0.80, 0.98]	77
Clinical Features + GADA + IA2 + T1D GRS	0.95 [0.90, 1.00]	71

Supplementary Table 9: ROC AUC calculated including only patients aged 25-35 years (inclusive) at diagnosis and with BMI 25-35 kg/m^2 (inclusive).

Participants from Young Diabetes in Oxford study studies meeting eligibility criteria (clinical diagnosis of T1D or T2D and age between 18 and 50 years) (n =856)

Missing data excluded participants (n = 87)

- Missing C-peptide: 9
- Treatment not known: 18
- Time to insulin or duration not available: 50
- BMI not available: 10

Unable to assign outcome excluded participants (n = 187)

- Diabetes duration <=36 months and not insulin treated: 91
- Insulin treated <=36 months and missing C-peptide: 72
- Insulin treated <=36 months and C-peptide >=200 pmol/L and <=600 pmol/L: 24

Participants selected for clinical features model external validation (n = 582, T1D = 134)

Excluded - GADA not tested: 33

Participants selected for clinical features + GADA model external validation (n = 549, T1D = 122)

Supplementary Figure 3: Flow diagram of participants through the model external validation stages.
T1D: type 1 diabetes, T2D: type 2 diabetes

Lynam A, *et al. BMJ Open* 2019; 9:e031586. doi: 10.1136/bmjopen-2019-031586

Characteristic	Model 1 development n = 1,352	Model 1 validation n = 582	comparison p value	Model 2 development n = 1,036	Model 2 validation n = 549	comparison p value
Sex (% Male)	59%	61%	>0.1	59%	61%	> 0.1
Age at diagnosis (years)	43 (36, 48)	37 (30, 41)	<0.001	43 (36, 48)	37 (30, 41)	< 0.001
BMI (kg/m^2)*	33 (28, 38)	31 (27, 36)	<0.001	33 (28, 38)	31 (27, 36)	< 0.001
Duration of diabetes (years)*	13 (8, 20)	14 (8, 23)	0.03	13 (8, 20)	13 (8, 23)	> 0.1
Type 1 diabetes	13%	23%	<0.001	14%	22%	< 0.001
HbA1c (%)*	8.2 (7.1, 9.6)	8.1 (7.2, 9.3)	>0.1	8.3 (7.3, 9.8)	8.1 (7.2, 9.4)	0.08
HbA1c (mmol/mol)*	66 (54, 81)	65 (55, 78)	>0.1	67 (56, 84)	65 (55, 79)	0.08
GADA (% positive)	-	-	-	12%	20%	< 0.001

Supplementary Table 10: Baseline characteristics comparison of the development and validation data sets for: Model 1 – Clinical features (Age at diagnosis & BMI) and Model 2 – Clinical features + GADA. *Measured at recruitment (median 13 years and 14 years post diagnosis in development data sets and validation data sets). Kruskal-Wallis used for comparison testing continuous variables, chi-square for categorical variables.

Performance parameter	External validation
**Clinical features model (n = 582)**	
ROC [95% CI]	0.86 [0.83, 0.90]
Expected/Observed	1.06
Calibration-in-the-large ($a \mid b_L$=1)	-0.14
Calibration slope (b_L)	0.85
Overall misclassification	-0.14 p = 0.05
Brier Score*	0.11 (p = 0.14)
**Clinical features + GADA model (n = 549)**	
ROC [95% CI]	0.93 [0.90, 0.96]
Expected/Observed	1.08
Calibration-in-the-large ($a \mid b_L$=1)	-0.23
Calibration slope (b_L)	0.90
Overall misclassification	-0.10 p > 0.1
Brier Score*	0.08 (p = 0.29)

Supplementary Table 11: Model performance results for the external validation of the clinical features and clinical features+ GADA models. * P value for Brier score is Spiegelhalter's z-test used to evaluate the calibration component of the Brier score, significant p-values indicate poor calibration.

Clinical features	Development (n = 1,352)					Validation (n = 582)				
	Probability cut-off for classifying type 1 diabetes					Probability cut-off for classifying type 1diabletes				
	10	30	50	70	90	10	30	50	70	90
Sensitivity/specificity (%)	85/79	64/95	49/98	35/99	15/100	91/62	73/85	59/93	45/96	13/99
Accuracy (%)	80	90	91	90	89	69	82	85	84	79
Positive predictive value (PPV) (%)	38	64	79	83	90	42	59	71	77	77
Negative predictive value (NPV) (%)	97	95	93	91	89	96	91	88	85	79

Clinical features + GADA	Development (n = 1,036)					Validation (n = 549)				
	Probability cut-off for classifying type 1 diabetes					Probability cut-off for classifying type 1diabletes				
	10	30	50	70	90	10	30	50	70	90
Sensitivity/specificity (%)	90/88	80/96	66/97	52/99	31/100	97/75	86/89	75/93	55/96	42/97
Accuracy (%)	89	94	93	92	90	80	88	88	87	85
Positive predictive value (PPV) (%)	55	75	80	85	92	53	69	73	80	81
Negative predictive value (NPV) (%)	98	97	95	93	90	99	96	93	88	85

Supplementary Table 12: Classification table comparing the development and validation samples at different cut-offs for probability of type 1 diabetes using the clinical features and clinical features + GADA logistic regression models.

Accuracy = (true positives + true negatives)/total number of participants.

Positive predictive value (PPV) = (sensitivity × prevalence)/[(sensitivity × prevalence) + ([1 – specificity] × [1−prevalence])].

Negative predictive value (NPV) = [specificity × (1 − prevalence)]/[(specificity × [1 − prevalence]) + ([1 − sensitivity] × prevalence)].

PPV and NPV assume prevalence for type 1 diabetes: Clinical features model – 13% (development) and 23% (validation), Clinical features + GADA model - 14% (development) and 22% (validation).

Lynam A, et al. BMJ Open 2019; 9:e031586. doi: 10.1136/bmjopen-2019-031586

Age at diagnosis (years)	BMI (kg/m²)	GADA positive	C-Peptide (PmolL)*	Insulin Treated	Time to insulin (months)	Duration at screening (years)†	Actual diabetes outcome	Probability of type 1 diabetes‡ (%)
18	26	0	775	1	Immediate	15	Type 2 diabetes	80
21	23	0	868	1	Immediate	10	Type 2 diabetes	82
27	29	1	-	0	-	3	Type 2 diabetes	88
38	22	1	550	1	48	10	Type 2 diabetes	88
36	22	1	175	1	72	12	Type 2 diabetes	89
23	32	1	25	1	48	29	Type 2 diabetes	90
30	25	1	25	1	36	30	Type 2 diabetes	91
29	25	1	225	1	48	12	Type 2 diabetes	93
23	28	1	50	1	120	28	Type 2 diabetes	95
33	21	1	65	1	96	47	Type 2 diabetes	95
34	20	1	25	1	120	22	Type 2 diabetes	96
23	22	1	-	0	-	3	Type 2 diabetes	99

Supplementary table 13: Characteristics of participants with probability of Type 1 diabetes > 80% but with type 2 diabetes actual outcome *Non fasting equivalent, measured > 5 years post diagnosis (unless < 200 PmolL prior to 5 years). † C-peptide measured at single screening visit. ‡Clinical features + GADA model applied to participants in the YDX study.

Age at diagnosis (years)	BMI (kg/m^2)	GADA positive	C-Peptide (PmolL)*	Insulin Treated	Time to insulin (months)	Duration at screening (years)†	Actual diabetes outcome	Probability of type 1 diabetes (%)‡
41	40	0	50	1	12	41	Type 1 diabetes	0.6
40	34	0	198	1	12	34	Type 1 diabetes	1.8
43	31	0	125	1	3	1	Type 1 diabetes	2.1
39	33	0	25	1	24	17	Type 1 diabetes	2.5
38	25	0	68	1	Immediate	19	Type 1 diabetes	12.7
39	40	1	50	1	Immediate	16	Type 1 diabetes	14.9

Supplementary table 14: Characteristics of participants with probability of Type 1 diabetes < 16% (Youden's Index cut-off) but with type 1 diabetes actual outcome *Non-fasting equivalent, measured > 5 years post diagnosis (unless < 200 pmolL prior to 5 years). † C-peptide measured at single screening visit. ‡Clinical features + GADA model applied to participants in the YDX study.

Model	ROC [95% CI]	Jack-knife cross validation *
Clinical features + IA-2	0.93 [0.90, 0.95]	0.07
Clinical features + T1D GRS	0.93 [0.90, 0.95]	0.08
Clinical features + IA-2 + T1D GRS	0.95 [0.93, 0.97]	0.06
Clinical features + GADA + T1D GRS	0.97 [0.96, 0.98]	0.07

Supplementary table 15: Model performance results for the four additional models in the online calculator. * Result reported as raw cross-validation estimate of prediction error with misclassification cost function (cut-off 0.5). cv.glm function in R version 3.3.3.

Included	β (SE)	Odds Ratio [95% CI]	p value
Constant (intercept)	37.94 (2.67)	-	-
Age at diagnosis (years) *	-5.09 (0.41)	0.006 [0.003, 0.014]	<0.001
BMI (kg/m^2) *	-6.34 (0. 60)	0.002 [0.001, 0.005]	<0.001

Supplementary Table 16: Clinical features logistic regression model (model 1). * Log transformed. Linear Predictor mean -2.96, sd 1.98

Included	β (SE)	Odds Ratio [95% CI]	p value
Constant (intercept)	-0.98 (0.19)	-	-
Model 1 linear predictor	0.94 (0.08)	2.57 (2.18, 3.03)	< 0.001
GADA positive	3.11 (0.32)	22.50 (12.13, 41.76)	< 0.001

Supplementary Table 17: Clinical features + GADA logistic regression model (model 2). Linear Predictor mean -3.37, sd 2.53

Lynam A, *et al. BMJ Open* 2019; 9:e031586. doi: 10.1136/bmjopen-2019-031586

Included	β (SE)	Odds Ratio [95% CI]	p value
Constant (intercept)	-1.28 (0.21)	-	
Model 1 linear predictor	0.92 (0.09)	2.50 [2.10, 2.98]	< 0.001
Antibody status - GADA positive only	3.08 (0.35)	21.81 [11.06, 43.02]	< 0.001
Antibody status - IA-2 positive only	3.49 (0.78)	32.93 [7.11, 152.64]	< 0.001
Antibody status - GADA & IA-2 both positive	4.35 (0.75)	77.53 [17.74, 338.84]	< 0.001

Supplementary Table 18: Clinical features + GADA + IA-2 logistic regression model (model 3). Linear Predictor mean -3.55, sd 2.58

Included	β (SE)	Odds Ratio [95% CI]	p value
Constant (intercept)	-0.67 (0.24)	-	-
Model 3 linear predictor	0.88 (0.08)	2.40 [2.06, 2.80]	< 0.001
T1D GRS (per 1 SD change)	1.08 (0.21)	2.93 [1.96, 4.39]	< 0.001

Supplementary Table 19: Clinical features + GADA + IA-2 + T1D GRS logistic regression model (model 4). T1D GRS standardized using mean 0.2356997, sd 0.0363499. Linear Predictor mean -3.74, sd 2.89.

Included	β (SE)	Odds Ratio [95% CI]	p value
Constant (intercept)	-0.36 (0.17)	-	-
Model 1 linear predictor	0.99 (0.08)	2.70 [2.30, 3.16]	< 0.001
IA-2 positive	3.19 (0.55)	24.39 [8.27, 71.92]	< 0.001

Supplementary Table 20: Clinical features + IA-2 logistic regression model. Linear Predictor mean -3.17, SD 2.28

Lynam A, *et al. BMJ Open* 2019; 9:e031586. doi: 10.1136/bmjopen-2019-031586

Included	β (SE)	Odds Ratio [95% CI]	p value
Constant (intercept)	-0.65 (0.18)	-	-
Model 1 linear predictor	0.87 (0.07)	2.39 [2.09, 2.74]	< 0.001
T1D GRS (per 1 SD change)	1.22 (0.15)	3.38 [2.51, 4.54]	< 0.001

Supplementary Table 21: Clinical features + T1D GRS logistic regression model. T1D GRS standardized using mean 0.2360879, sd 0.0358468. Linear Predictor mean -3.180108, sd 2.401089.

Included	β (SE)	Odds Ratio [95% CI]	p value
Constant (intercept)	-1.12 (0.23)	-	-
Model 1 linear predictor	0.87 (0.09)	2.40 [2.02, 2.84]	< 0.001
T1D GRS (per 1 SD change)	1.36 (0.20)	3.89 [2.64, 5.74]	< 0.001
IA-2 positive	2.95 (0.65)	19.17 [5.33, 68.81]	< 0.001

Supplementary Table 22: Clinical features + IA-2 + T1D GRS logistic regression model. T1D GRS standardized using mean 0.235673, sd 0.0363399. Linear Predictor mean -3.537275, sd 2.79395.

Included	β (SE)	Odds Ratio [95% CI]	p value
Constant (intercept)	-1.50 (0.24)	-	-
Model 1 linear predictor	0.85 (0.09)	2.33 [1.97, 2.76]	< 0.001
T1D GRS (per 1 SD change)	1.12 (0.20)	3.05 [2.09, 4.46]	< 0.001
GADA positive	2.63 (0.34)	13.89 [7.17, 26.90]	< 0.001

Supplementary Table 23: Clinical features + GADA + T1D GRS logistic regression model. T1D GRS standardized using mean 0.2359649, sd 0.0363407. Linear Predictor mean - 3.596086, sd 2.868552.

Model	Linear predictor (lp) regression equation*
Clinical features	37.94 + (-5.09 * log(age)) + (-6.34 * log(BMI))
Clinical features + GADA†	34.8057844720 + (-4.801441792 * log (Age)) + (-5.980577792 * log(BMI)) + (2.937107976 * GADA†)
Clinical features + GADA + IA-2	33.49649577 + (-4.665598345 * Log(Age)) + (-5.81137397 * Log(BMI)) + (3.082366 * AntiStatus1‡) + (3.494462 * AntiStatus2‡) + (4.350717 * AntiStatus3‡)
Clinical features + GADA + IA-2 + T1D GRS	21.57649882 + (-4.086215772 * Log(Age)) + (-5.096252172 * Log(BMI)) + (2.702010666 * AntiStatus1‡) + (3.063255174 * AntiStatus2‡) + (3.813850704 * AntiStatus3‡) + (30.11052 * T1D GRS)
Clinical features + IA-2	37.26905033 + (3.194096 * IA-2†) + (-5.047657308 * Log(Age)) + (-6.287258808 * Log(BMI))
Clinical features + T1D GRS	24.46138054 + (-4.443506884 * Log(Age)) + (-5.534741384 *Log(BMI)) + (33.93968 * T1D GRS)
Clinical features + IA-2 + T1D GRS	23.2151829 +(2.953142 * IA-2†) + (-4.446784844 *Log(Age))+(-5.538824344 * Log(BMI)) + (37.40205 * T1D GRS)
Clinical features + GADA + T1D GRS	23.20924904 + (2.63093 * GADA†) + (-4.303557843 * Log(Age)) + (-5.360423718 *Log(BMI)) + (31.22606 * T1D GRS)

Supplementary Table 24: *To convert to probability use exp(lp)/(1+exp(lp)). †Dummy variable: negative = 0, positive = 1 ‡Dummy variables: false = 0, true = 1, AntiStatus1 = GADA positive only, AntiStatus2 = IA-2 positive only, AntiStatus3 = Both GADA and IA-2 positive.

Lynam A, *et al. BMJ Open* 2019; 9:e031586. doi: 10.1136/bmjopen-2019-031586
