# Supplementary material for: Development and validation of multivariable clinical diagnostic models to identify type 1 diabetes requiring rapid insulin therapy in adults aged 18–50 years
Source: BMJ Open. 2019 Sep 26;9(9):e031586. doi: 10.1136/bmjopen-2019-031586 (PMC6773323; doi:10.1136/bmjopen-2019-031586)
Supplement: Author's manuscript [file bmjopen-2019-031586.draft_revisions.pdf]

BMJ Open

BMJ Open is committed to open peer review. As part of this commitment we make the peer review history of every article we publish publicly available.

When an article is published we post the peer reviewers' comments and the authors' responses online. We also post the versions of the paper that were used during peer review. These are the versions that the peer review comments apply to.

The versions of the paper that follow are the versions that were submitted during the peer review process. They are not the versions of record or the final published versions. They should not be cited or distributed as the published version of this manuscript.

BMJ Open is an open access journal and the full, final, typeset and author-corrected version of record of the manuscript is available on our site with no access controls, subscription charges or pay-per-view fees (http://bmjopen.bmj.com).

If you have any questions on BMJ Open's open peer review process please email info.bmjopen@bmj.com

BMJ Open

Development and validation of multivariable clinical prediction models to identify type 1 diabetes requiring rapid insulin therapy in adults aged 18 to 50

Journal:	*BMJ Open*
Manuscript ID	bmjopen-2019-031586
Article Type:	Research
Date Submitted by the Author:	10-May-2019
Complete List of Authors:	Lynam, Anita; University of Exeter Medical School, The Institute of Biomedical & Clinical Science<br>McDonald, Timothy; Royal Devon and Exeter NHS Foundation Trust; University of Exeter Medical School, The Institute of Biomedical & Clinical Science<br>Hill, Anita; University of Exeter Medical School, The Institute of Biomedical & Clinical Science<br>Dennis, John; University of Exeter Medical School, The Institute of Biomedical & Clinical Science<br>Oram, Richard; University of Exeter, The Institute of Biomedical & Clinical Science; Royal Devon and Exeter NHS Foundation Trust<br>Pearson, Ewan; University of Dundee<br>Weedon, Michael; University of Exeter<br>Hattersley, Andrew; University of Exeter Medical School, Institute of Biomedical Science<br>Owen, Katharine; Oxford Centre for Diabetes Endocrinology and Metabolism<br>Shields, Beverley; University of Exeter,<br>Jones, Angus; University of Exeter Medical School, The Institute of Biomedical & Clinical Science; University of Exeter
Keywords:	type 1 diabetes, type 2 diabetes, c-peptide, islet autoantibodies, prediction model, genetic risk score

SCHOLARONE™
Manuscripts

Title

Development and validation of multivariable clinical prediction models to identify type 1 diabetes requiring rapid insulin therapy in adults aged 18 to 50

Authors' names

Anita L Lynam, Timothy J McDonald, Anita V Hill, John M Dennis, Richard A Oram, Ewan R Pearson, Michael N Weedon, Andrew T Hattersley, Katharine R Owen, Beverley M Shields*, Angus G Jones*

* These authors contributed equally

Address for each author

1. Anita L Lynam, PhD student, Institute of Biomedical and Clinical Science, College of Medicine and Health, University of Exeter, Exeter, EX2 5DW, UK
2. Timothy J McDonald, Honorary Clinical Associate Professor, Academic Department of Clinical Biochemistry, Royal Devon and Exeter NHS Foundation Trust and Institute of Biomedical and Clinical Science, College of Medicine and Health, University of Exeter, Exeter, EX2 5DW, UK,
3. Anita V Hill, Research Project Manager, NIHR Exeter Clinical Research Facility, University of Exeter College of Medicine & Health, Exeter, EX2 5DW,
4. John M Dennis, Research Fellow in Medical Statistics, Institute of Biomedical and Clinical Science, College of Medicine and Health, University of Exeter, Exeter, EX2 5DW, UK,
5. Richard A Oram, Diabetes UK Harry Keen Fellow, Institute of Biomedical and Clinical Science, College of Medicine and Health, University of Exeter, Exeter, EX2 5DW, UK
6. Ewan R Pearson, Professor of Diabetic Medicine, Division of Molecular and Clinical Medicine, School of Medicine, University of Dundee, Ninewells Hospital and Medical School, Dundee, U.K
7. Michael N Weedon, Associate Professor, Institute of Biomedical and Clinical Science, College of Medicine and Health, University of Exeter, Exeter, EX2 5DW, UK,
8. Andrew T Hattersley, Professor of Molecular Medicine, Institute of Biomedical and Clinical Science, College of Medicine and Health, University of Exeter, Exeter, EX2 5DW, UK,
9. Katharine R Owen, Associate Professor of Diabetes, Oxford Centre for Diabetes Endocrinology and Metabolism, University of Oxford, Churchill Hospital, Oxford, OX3 7LE, UK and Oxford NIHR Biomedical Research Centre, Oxford University Hospitals Foundation Trust, John Radcliffe Hospital, Oxford, UK,

For peer review only - http://bmjopen.bmj.com/site/about/guidelines.xhtml

Corresponding authors

10. Beverley M Shields, Senior Lecturer in Medical Statistics, Institute of Biomedical and Clinical Science, College of Medicine and Health, University of Exeter, Exeter, EX2 5DW, UK

Email: B.Shields@exeter.ac.uk

Phone: +44 1392 408203

11. Angus G Jones, NIHR Clinician Scientist, Institute of Biomedical and Clinical Science, College of Medicine and Health, University of Exeter, Exeter, EX2 5DW

angus.jones@exeter.ac.uk

Phone: +44 1392 408538

For peer review only

Word count: abstract 299 main article 3,857

Tables: 1

Figures: 3

Key Words

Type 1 diabetes

Type 2 diabetes

Classification

C-peptide

GAD

IA-2

Type 1 Diabetes Genetic Risk Score

Abbreviations

GADA: Glutamic acid decarboxylase autoantibody

IA-2A: Islet antigen-2 autoantibody

ROC AUC: Area under the receiver operating characteristic curve

T1D GRS: Type 1 Diabetes Genetic Risk Score

YDX: Young Diabetes in Oxford Study

For peer review only - http://bmjopen.bmj.com/site/about/guidelines.xhtml

Summary box

What is already known on this topic:

- Current guidance on diabetes classification at diagnosis focus on etiopathological definitions with no clear criteria for use in clinical practice.
- Misclassification of diabetes subtype is common, particularly in young adult patients where due to increasing rates of obesity discriminating between type 1 and young-onset type 2 diabetes can be challenging.
- There are no clinical prediction models available to assist clinicians distinguish between type 1 and type 2 diabetes.

What this study adds:

- Clinical prediction models integrating clinical features with biomarkers have high accuracy for identifying type 1 diabetes with rapid insulin requirement in both internal and external validation.
- The development of multiple models allows a staged approach to classification of diabetes, with a clinical features only model used to identify patients with diagnostic uncertainty who may benefit from additional testing.

For peer review only

Abstract

Objective:

To develop and validate multivariable clinical prediction models to assist distinguishing between type 1 and type 2 diabetes in adults aged 18 to 50.

Design:

Multivariable logistic regression analysis was used to develop classification models integrating five pre-specified predictor variables, including clinical features (age of diagnosis, BMI) and clinical biomarkers (GAD and Islet Antigen 2 islet autoantibodies, Type 1 Diabetes Genetic Risk Score), to identify type 1 diabetes with rapid insulin requirement using data from existing cohorts.

Setting:

United Kingdom cohorts recruited from primary and secondary care.

Participants:

1,352 (model development) and 582 (external validation) participants diagnosed with diabetes between the age of 18 and 50 years of white European origin.

Main outcome measures:

Type 1 diabetes was defined by rapid insulin requirement (within 3 years of diagnosis) and severe endogenous insulin deficiency (C-peptide <200pmol/L). Type 2 diabetes was defined by either a lack of rapid insulin requirement or, where insulin treated within 3 years, retained endogenous insulin secretion (C-peptide >600pmol/L at ≥5 years diabetes duration). Model performance was assessed using area under the receiver operating characteristic curve (ROC AUC), and internal and external validation.

For peer review only - http://bmjopen.bmj.com/site/about/guidelines.xhtml

Results:

Type 1 diabetes was present in 13% of participants in the development cohort. All five predictor variables were discriminative and independent predictors of type 1 diabetes ($p<0.001$ for all) with individual ROC AUC ranging from 0.82 to 0.85. Model performance was high: ROC AUC range 0.90 [95%CI 0.88, 0.93] (clinical features only) to 0.97 [0.96, 0.98] (all predictors) with low prediction error. Results were consistent in external validation (clinical features and GADA ROC AUC 0.93 [0.90, 0.96]).

Conclusions:

Clinical prediction models integrating clinical features with biomarkers have high accuracy for identifying type 1 diabetes with rapid insulin requirement, and could assist clinicians and researchers in accurately identifying patients with type 1 diabetes.

For peer review only

For peer review only - http://bmjopen.bmj.com/site/about/guidelines.xhtml

Strengths and Limitations of this study

- Diabetes type is robustly defined using direct measurement of endogenous insulin secretion, an outcome closely related to treatment, education and monitoring requirements.
- A combination of a large development dataset and small number of predictors minimises risk of model overfitting, a common problem with prediction models of this nature.
- Models are robustly internally and externally validated
- The cross section nature of the development and validation cohorts means that time to insulin was self-reported and measurement of model predictors was not undertaken at diagnosis: both BMI and islet autoantibody prevalence may change over time.
- Models have been developed in white European populations with young adult onset diabetes: further work is required to extend this work to other age groups and ethnicities.

For peer review only

For peer review only - http://bmjopen.bmj.com/site/about/guidelines.xhtml

Introduction

Making the correct diagnosis of type 1 and type 2 diabetes is crucial for appropriate management, with guidelines for these conditions recommending very different glucose-lowering treatment and education [1-3]. These differences are predominantly driven by the rapid development of severe endogenous insulin deficiency in type 1 diabetes [1]. This means that patients with type 1 diabetes need rapid insulin treatment and are at risk of life-threatening ketoacidosis without insulin treatment. They develop a requirement for physiological insulin replacement (e.g. multiple injections, carbohydrate counting, pumps) due to the very high glycaemic variability associated with severe insulin deficiency [4, 5] and have poor glycaemic response to most adjuvant glucose-lowering therapies [6]. In contrast, patients with type 2 diabetes continue to make substantial endogenous insulin even many decades after diagnosis [7]. Glycaemia is therefore usually managed initially with lifestyle change or oral agents [4, 8] and, if insulin treatment is needed, a combination of simple insulin regimens and adjuvant non-insulin therapies [4, 5, 8, 9].

For peer review only

Correctly distinguishing between diabetes subtypes at diagnosis is often difficult and misclassification therefore common [10-12]. Current guidelines focus on etiopathological definitions without giving clear criteria for clinical use [1, 13]. In clinical practice, clinical features are predominantly used to determine diabetes subtype but only age at diagnosis and BMI have evidence for utility at diabetes onset, whereas other features used by clinicians such as symptoms at diagnosis, weight loss or ketosis do not have an evidence base [14]. Increasing obesity rates mean that many patients with type 1 diabetes will be obese and type 2 diabetes is occurring in the young [15]. Type 1 diabetes has been recently shown to occur at similar rates in those aged above and below 30 [16]. Therefore simple cut-offs based on age at diagnosis and BMI are unlikely to accurately diagnose diabetes type for many patients [1, 10]. Similarly, there is no single diagnostic test that can be used to classify diabetes robustly at diagnosis. While measurement of islet autoantibodies can assist classification, many patients with type 1 diabetes are islet-autoantibody-negative and many patients with the clinical phenotype of type 2 diabetes, without rapid insulin requirement, are islet-autoantibody-positive [17]. A type 1 genetic risk score has been recently shown to assist diagnosis of diabetes type but this provides imperfect discrimination in isolation [18] .

For peer review only

In order to classify diabetes a suitable "gold standard" is necessary. As the key factor driving differences in treatment decisions between the two subtypes is the lack of endogenous insulin secretion, direct measurement of endogenous insulin secretion in longstanding insulin-treated diabetes (>3-5 years), using C-peptide, provides a robust classification that closely relates to treatment requirements [19]; patients with severe endogenous insulin deficiency (low C-peptide) have the high glucose variability, absolute insulin requirement, and lack of response to non-insulin glucose-lowering therapies that are characteristic of type 1 diabetes, regardless of their clinical characteristics and clinician's diagnosis [7, 11, 19-23]. However, this test may have limited utility at diagnosis, as patients with recent onset type 1 diabetes may have retained endogenous insulin secretion [21, 24].

Clinical prediction models offer a way of combining multiple patient features and biomarkers to improve accuracy of diagnosis or prognosis. In diabetes, diagnostic models combining clinical features are available to predict the risk of prevalent or incident type 2 diabetes [25] and there is a model to identify monogenic forms of diabetes in patients with young-onset diabetes [26]. However there are no models to help distinguish type 1 and type 2 diabetes at diagnosis. We therefore aimed to develop and validate diagnostic multivariable clinical prediction models that combine clinical features and biomarkers to identify type 1 diabetes (defined by rapid insulin requirement and severe endogenous insulin deficiency) in patients aged between 18 and 50 years at diabetes diagnosis.

For peer review only - http://bmjopen.bmj.com/site/about/guidelines.xhtml

Methods

We used logistic regression to model the relationship between each of clinical features and biomarkers, and type 1 diabetes defined by rapid insulin requirement and severe endogenous insulin deficiency (see below). We assessed the performance of the models using both internal validation and external validation.

Study population – development cohort

For model development, participants were identified from Exeter, UK-based cohorts [27-30]. These cohorts were participants with clinically diagnosed diabetes recruited from primary and secondary care. Summaries of the cohorts including recruitment and data collection methods are shown in Supplementary Table 1.

Participants were eligible for the study (model development or validation) if they had a clinical diagnosis of diabetes between the ages of 18 and 50 years. Participants with known secondary or monogenic diabetes [31], or a known disorder of the exocrine pancreas [32], were excluded. All participants included in this study were of white European origin.

Study population - external validation cohort

Participants meeting the study inclusion criteria were identified in the Young Diabetes in Oxford (YDX) study [33]. YDX is a cross-sectional study of participants diagnosed with diabetes (of any type) up to the age of 45 years, recruited from primary and secondary care in the Thames Valley region, UK. Participants with known secondary, pancreatic or monogenic diabetes were excluded.

Ethical approval

All cohort studies used for this research received ethical approval from the UK National Research Ethics Service. All participants gave written informed consent.

For peer review only

For peer review only - http://bmjopen.bmj.com/site/about/guidelines.xhtml

Model outcome: type 1 and type 2 diabetes definition

Type of diabetes was defined by the presence or absence of rapid insulin requirement and severe endogenous insulin deficiency after a diagnosis of diabetes, as follows:

Type 1 diabetes: Insulin treatment within <= 3 years of diabetes diagnosis and severe insulin deficiency (non–fasting C peptide < 200pmol/L) [21].

Type 2 diabetes: Either 1) no insulin requirement for 3 years from diabetes diagnosis or 2) where insulin was started within 3 years of diagnosis, substantial retained endogenous insulin secretion (C-peptide >600pmol/L) at >=5 years diabetes duration.

Cohort participants not meeting the above criteria or with insufficient information were excluded from analysis, as type of diabetes and rapid insulin requirement could not be robustly defined.

Model predictors

Five pre-specified predictor variables were assessed, based on prior evidence and availability: age at diagnosis [14], BMI [14], GAD and IA-2 islet autoantibodies [17, 34], and a Type 1 diabetes Genetic Risk Score (T1D GRS) [18].

Assessment of clinical features

At study recruitment visit, clinical history including time to insulin and age at diagnosis were self-reported by participants in an interview with a research nurse. Height and weight were measured for calculation of BMI.

Laboratory Measurement

C-peptide

In the development cohort, C-peptide was measured on stored EDTA taken at study

For peer review only - http://bmjopen.bmj.com/site/about/guidelines.xhtml

visits (non-fasting random [35], fasting, or at 90 minutes in a post-mixed-meal tolerance test (majority 87% non-fasting)). With specific additional consent, C-peptide was also measured on post-recruitment non-fasting EDTA samples collected as part of routine clinical care. Fasting C-peptide values were multiplied by 2.5 to non-fasting equivalent [21]. The median C-peptide value was used where more than one eligible C-peptide value was available (62% of participants requiring this measure for outcome definition). C-peptide was measured using an electrochemiluminescence immunoassay on a Roche Diagnostics E170 analyser (Roche, Mannheim, Germany) by the Academic Department of Blood Sciences at the Royal Devon and Exeter Hospital. In the external validation cohort, C-peptide measurement was performed in the Biochemistry Laboratory of the Oxford University Hospitals NHS Trust using a chemiluminescence immunoassay on an ADVIA Centaur analyser (Siemens Healthcare Diagnostics Ltd).

Islet autoantibodies

In the development cohort, GADA and IA-2A were measured on EDTA taken at recruitment or obtained from local laboratory records. Both islet autoantibodies were measured using the RSR Ltd ELISA assays (RSR Ltd, Cardiff, UK) on the Dynex DS2 ELISA Robot (Dynex Technologics, Worthing, UK) by the Academic Department of Blood Sciences at the Royal Devon and Exeter Hospital. The department participates in the International Autoantibody Standardization Programme. The cut-off for positivity for GADA was ≥11 units/ml and IA-2A was ≥15 units/ml, based on the 97.5th centile of 1,559 controls without diabetes [34].

In the external validation cohort, GADA was measured by a radioimmunoassay using ^{35}S-labeled full-length GAD65 by the Department of Clinical Science, University of Bristol, Bristol, U.K. Results were expressed in World Health Organization (WHO)

For peer review only - http://bmjopen.bmj.com/site/about/guidelines.xhtml

units per millilitre derived from a standard curve calibrated from international reference material (National Institute for Biological Standards and Control code 97/550). The cut-off for positivity for GADA was 13 WHO Units/mL initially, using a local assay (samples measured n=218, DASP2010 sensitivity 88% at 93% specificity) and changed to 33 DK Units/mL later in the study (standard assay, DASP2010 sensitivity 80%, specificity 97%).

Type 1 Diabetes Genetic Risk Score (T1D GRS)

The T1D GRS was calculated on the development cohort as previously described [18]. In brief, T1D GRS consists of 30 common type 1 diabetes genetic variants (single nucleotide polymorphisms (SNPs)) from HLA and non-HLA loci; each variant is weighted by its effect size on type 1 diabetes risk from previously published literature, with weights for DR3/DR4-DQ8 assigned based on imputed haplotypes (Supplementary Table 2). All SNPs had an INFO > 0.8. The combined score represents an individual's genetic susceptibility to type 1 diabetes. T1D GRS calculation was not performed if genotyping results were missing for either of the two alleles with the greatest weighting (DR3/DR4-DQ8 or HLA_DRB1_15) or if more than two of any other SNPs were missing. For ease of clinical interpretation the score is presented in this article as the score and centile position of the distribution in the Wellcome Trust Case Control Consortium type 1 diabetes population [36].

Statistical analysis

Model development

We used logistic regression analysis to develop the models. Models were developed on a complete-case basis.

For peer review only

For peer review only - http://bmjopen.bmj.com/site/about/guidelines.xhtml

Age at diagnosis, BMI and T1D GRS were modelled as continuous variables and transformations used to ensure linearity on the logit scale [37] (Supplementary Figure 1). GADA and IA-2A were both dichotomized into negative or positive based on the cut-off for positivity in line with how the results are reported clinically [2]. Sample sizes were checked using both minimal Events Per Variable (EPV) criteria (>=10) [38] and square root of the mean squared prediction error (rMPSE) [39] and were considered sufficient for reliable prediction modelling.

As some participants had missing diagnostic test data, models were built and validated in four stages to maximise the sample size at each stage: 1) model including only clinical features (age at diagnosis and BMI); 2) Addition of GADA to the linear predictor from model 1; 3) Addition of both GADA and IA-2A to the linear predictor from model 1; 4) Addition of T1D GRS to model 3 linear predictor.

Evaluation of model performance: Internal validation

Three internal validation techniques were used to assess the discrimination and calibration performance of the models: 1) directly using the data used to develop the model (apparent validation, ROC AUC); 2) Jack-knife cross-validation; 3) Bootstrapping (with replacement method) [37]

Evaluation of model performance: External validation

Performances of model 1 (clinical features) and model 2 (clinical features + GADA), were evaluated in the YDX study cohort. We were unable to externally evaluate models 3 and 4 as IA-2 autoantibodies and T1D GRS were not available in the YDX study.

For peer review only - http://bmjopen.bmj.com/site/about/guidelines.xhtml

Model comparisons

Four nested replica models were built on the subset of participants with complete data on all predictor variables (n = 943). The predictive information of each additional predictor on the model performance was assessed using the Unitless Index of Adequacy [37], log likelihood ratio test [37], Net Reclassification Improvement and Integrated Discrimination Improvement [40] .

Sensitivity analysis

Model development of all 4 models was repeated on 943 participants with complete data.

All statistical analyses were performed using STATA version 15, STATA Corp, Texas, USA (unless otherwise stated).

Patient Involvement

Patients with diabetes were involved in prioritising the research question and development of the original funding application. This study did not involve the collection of primary data, but this research was reviewed and access to data approved by the Peninsula Research Bank Lay steering committee, who also contributed to the design and development of the source cohort studies.

For peer review only

For peer review only - http://bmjopen.bmj.com/site/about/guidelines.xhtml

Results

1,352 (type 1 diabetes n = 179) participants met analysis inclusion criteria for the clinical features model with 943 participants having all predictor variables measured. 39 (22%) of the 179 participants with type 1 diabetes by the study definition had not been treated with insulin from diagnosis. Of those treated with continuous insulin from diagnosis, 29 (17%) had a model outcome of type 2 diabetes. A flow diagram detailing those excluded is shown in Supplementary Figure 2. Only 37 (2% of the cohort) had an undefinable outcome due to intermediate C-peptide levels (200-600pmol/L when insulin-treated within 3 years of diagnosis). The remaining exclusions were due to either missing data or short duration of diabetes. The characteristics and type 1 diabetes outcome prevalence of the included participants were similar in all four development samples (Supplementary Table 3). There were no clinically relevant differences in the characteristics of the participants who were excluded from the fourth model development stage (n = 409) (Supplementary Table 4). Islet autoantibodies and C-peptide were measured at median 13 years and 16 years post-diagnosis respectively.

Clinical features or biomarkers in isolation overlap substantially between diabetes types (Figure 1)

Participants with type 1 diabetes and rapid insulin requirement were diagnosed younger compared to the participants with type 2 diabetes (median 27 vs 44 years, $p < 0.001$) and had a lower BMI (median 26 vs 34 kg/m^2, $p < 0.001$). Positive autoantibodies (GADA, IA-2A or both) were more common in the participants with type 1 diabetes (71% of participants with type 1 diabetes vs 5% of participants with type 2 diabetes, $p < 0.001$). Patients with type 1 diabetes had a higher T1D GRS

For peer review only - http://bmjopen.bmj.com/site/about/guidelines.xhtml

(median 0.27 vs 0.23 (equivalent to 40th and 4th centile of the Wellcome Trust Case Control Consortium population with type 1 diabetes [36], $p < 0.001$). These features overlapped substantially between participants meeting criteria for type 1 and type 2 diabetes (Figure 1) with AUC ROC for these features in isolation: 0.82 (age at diagnosis), 0.83 (BMI), 0.83 (islet autoantibodies) and 0.85 (T1D GRS).

Combining clinical features using a prediction model improves model discrimination

In model 1, age at diagnosis and BMI were both significant independent predictors of type 1 diabetes, with the odds of having type 1 diabetes increasing with younger age at diagnosis and lower BMI. Combined, these features provided excellent discrimination (ROC AUC=0.904, perfect test = 1) (Figure 2a), with low probabilities capturing the majority of participants with type 2 diabetes and type 1 diabetes being very unlikely (Figure 2b; sensitivity, specificity, and positive and negative predictive values at various probability cut-offs are reported in Table 1). In successive models adding in GADA (model 2), then IA-2A (model 3) and then T1D GRS (model 4), the addition of each predictor to the previous model resulted in significant improvements in discrimination (Figure 2 and Supplementary Table 5) and model fit (Supplementary Tables 6 and 7). In sensitivity analysis, results were similar when restricting all models to only the 943 participants with complete data on all predictor variables (Supplementary Table 8).

Internal validation suggests robust model performance

Results of the internal validation bootstrap (Supplementary Table 5) indicate good model discrimination, with very similar model performance in bootstrapped samples (near identical ROC AUC for all models (max decrease = 0.0018)), high calibration indicating the predicted probabilities closely fit the observed probabilities (calibration

For peer review only - http://bmjopen.bmj.com/site/about/guidelines.xhtml

slope range 0.98 - 1.00 (0.9 – 1.1 is indicative of good calibration)), and very low levels of optimism suggesting little error due to overfitting.

Model performance remains high in an external validation cohort with different characteristics

582 participants in the YDX study met criteria for external validation (Supplementary Figure 3). Compared to the participants in the Exeter model development cohort, the participants in the YDX study were younger at diagnosis (consistent with the narrower age range in YDX (18-45y) (median 37 years vs 43 years, $p < 0.001$)), had a lower BMI (median 31 kg/m^2 vs 33 kg/m^2, $p < 0.001$), had a higher percentage of GADA (20% versus 12%, $p < 0.001$) and a higher prevalence of type 1 diabetes by study definition (22% vs 14%, $p < 0.001$) (see Supplementary Table 9 for participant characteristics).

There was a small decrease in performance of the model 1 (clinical features) and model 2 (clinical features and GADA) when they were applied to the external validation samples but both still showed high levels of discrimination despite differences in the two cohorts (ROC AUC = 0.865 and 0.930 for models 1 and 2, respectively, (Figure 3 and Supplementary Table 10)). Both models slightly over estimated type 1 diabetes prevalence but there was no evidence of miscalibration (Figures 3b and e, Supplementary Table 10). Sensitivity and specificity in the validation cohort are shown in Supplementary Table 11.

For peer review only - http://bmjopen.bmj.com/site/about/guidelines.xhtml

Participants with high model probability type 1 diabetes but type 2 diabetes outcome have the characteristics of type 1 diabetes but took > 3 years to commence insulin therapy.

Supplementary Table 12 shows the characteristics of 12 participants in the external validation cohort with >80% model type 1 diabetes probability, but an actual model outcome of type 2 diabetes. These participants had the clinical characteristics associated with type 1 diabetes with GADA positivity and low C-peptide in the majority of cases (median C-peptide 120 pmol/L). However the time to insulin was > 3 years in GADA positive cases, suggesting slow onset autoimmune diabetes. In contrast, the 6 participants who had a low model type 1 diabetes probability (< 16%) but an actual model outcome of type 1 diabetes (Supplementary Table 13) had features associated with type 2 diabetes.

Online calculator

The four models have been incorporated into an online calculator (beta version available at https://www.diabetesgenes.org/t1dt2d-prediction-model/). An additional four models with different combinations of the five predictor variables were also developed for the online calculator, to allow every combination of clinical features plus the other biomarkers as optional. As expected, ROC AUC and prediction error results for these four additional models were intermediate between the basic clinical features model and the full model with all features (see Supplementary Table 14).

Supplementary Tables 15 - 22 inclusive show the β coefficients and odds ratios for all models. The regression equations for the online calculator are shown in Supplementary Table 23.

For peer review only - http://bmjopen.bmj.com/site/about/guidelines.xhtml

Discussion

We have developed, evaluated and validated clinical prediction models combining age at diagnosis, BMI, GADA, IA-2A, and T1D GRS to provide estimates of a patient's risk of having type 1 diabetes requiring rapid insulin therapy from diagnosis. These models show high performance, and could potentially assist classification of diabetes in clinical practice and provide a tool for evidence based classification in research cohorts.

Model performance was optimised in the model combining all five predictors (ROC AUC 0.97). However, all models performed well with ROC AUC > 0.9 and low cross-validated prediction errors in development. The results of the external validation provide additional confidence in model performance. This was undertaken in a distinct dataset with different type 1 diabetes prevalence and biochemical assays.

This is the first study developing clinical prediction models for classification of type 1 and 2 diabetes. Key strengths of this study include our systematic approach to model development including robust internal and external validation [41]. Our staged approach to model development means that we have maximised the information gained from each predictor. Our model is parsimonious, we have used only five predictors previously shown to be associated with type 1 diabetes. This, in combination with large datasets, mean we have a high number of events per variable and very low risk of overfitting, a common problem with prediction models of this nature. Our use of predominantly population-based cohorts recruited largely from a primary care setting (for model development) means our results are likely to reflect true associations in patients seen in clinical practice. The overall prevalence of study defined type 1 diabetes of 13% in our development dataset is close to the 11% reported type 1 diabetes prevalence at diagnosis in a UK population aged 20-50 [42].

For peer review only - http://bmjopen.bmj.com/site/about/guidelines.xhtml

A limitation of our study is the cross-sectional nature of our cohorts meaning that age at diagnosis and time to insulin were self-reported at a single visit. Insulin commencement was also based on clinical decision-making rather than a trial protocol. BMI and antibodies were measured at median 13 years after diagnosis. BMI, and GAD and IA-2 antibodies change modestly over time in adult onset diabetes, with previous research suggesting an approximately 18% lower combined GADA and IA-2A prevalence after 13.5 years diabetes duration in this age group [43] , and BMI having higher discrimination for diabetes classification when measured at diagnosis [44]. The lack of information at diagnosis also meant we were unable to assess whether other features available at diagnosis may assist classification, such as presentation glycaemia, ketosis, or weight loss. A prospective study to prospectively validate these models, and assess whether other features may assist classification is therefore ongoing (https://clinicaltrials.gov/ct2/show/NCT03737799).

A further limitation is that this model has been developed and tested in a white European population with young onset diabetes, extension of this work to non-white populations and older age groups is therefore a priority for future research.

These models have the potential to help robustly classify diabetes in research cohorts, and may have particular utility where genetic but not antibody data is available, a common situation in many biobanks. They may also assist clinical decision making, with the important caveats that this evidence can only be applied to patients aged 18-50, of white ethnicity, and that these models are intended to act as a decision aid in conjunction with other information which a clinician may use to inform treatment decisions (for example severity of hyperglycaemia): they do not replace expert clinical opinion. A web-based calculator and smartphone app could be used to display the estimate of the patient’s probability of having type 1 diabetes

For peer review only - http://bmjopen.bmj.com/site/about/guidelines.xhtml

based on the predictor variable values entered. The models can be used with age of diagnosis and BMI as a minimum; users will then have a choice to add results of GADA, IA-2A and T1D GRS in any combination. This could therefore be used by clinicians as a triage-based approach to diabetes subtype diagnosis. For example, probabilities calculated on clinical features could be used as the basis for antibody testing, or the additional value likely to be gained from antibody or genetic testing could be assessed by inputting dummy results into the model. We propose providing the continuous probability outcome of the models rather than giving a threshold. This is because the decision made on whether to commence insulin for a given probability of type 1 diabetes will vary enormously due to other factors. For example temporary insulin treatment may be appropriate regardless of likely classification where hyperglycaemia is severe, and in some circumstances it may be appropriate to trial oral therapy even where type 1 diabetes has a high probability, for example where a person's occupation would be affected by insulin treatment and they can be carefully monitored for glycaemic deterioration.

In conclusion clinical prediction models integrating clinical features with biomarkers have high accuracy for identifying type 1 diabetes with rapid insulin requirement in white participants aged 18 to 50 at diabetes diagnosis, and may assist clinicians in identifying patients with type 1 diabetes in clinical practice.

Acknowledgments

The authors thank participants who took part in these studies and the research teams who undertook cohort recruitment. We thank Catherine Angwin of the NIHR Exeter Clinical Research Facility for assistance with data preparation, and Rachel

For peer review only - http://bmjopen.bmj.com/site/about/guidelines.xhtml

Nice of the Blood Sciences Department, Royal Devon and Exeter Hospital for assistance with sample analysis. We are grateful to Maarten van Smeden for allowing us to access to his Beyond EPV R-Shiny app (BETA version).

Transparency statement

The manuscript's guarantors (AGJ and BMS) affirms that the manuscript is an honest, accurate, and transparent account of the study being reported; that no important aspects of the study have been omitted; and that any discrepancies from the study as planned have been explained.

Author Contributions

A.L.L, B.M.S and A.G.J conceived the idea and designed the study. A.L.L, T.J.M, A.V.H, E.R.P, M.N.W, A.T.H, K.R.O and A.G.J researched the data. A.L.L analysed the data with assistance from B.M.S and A.G.J. T.J.M, J.M.D, R.A.O, A.T.H and K.R.O discussed and contributed to study design and provided support for the analysis and interpretation of results. A.L.L drafted the manuscript with assistance from B.M.S and A.G.J. All authors critically revised the manuscript and approved the final version. The corresponding author attests that all listed authors meet authorship criteria and that no others meeting the criteria have been omitted.

A.G.J. and BMS are the guarantors of this work.

Funding:

This work was funded by NIHR Clinician Scientist award CS-2015-15-018. The Young Diabetes in Oxford Study was funded by the European Community FP7 programme CEED3 (HEALTH-F2-2008-223211), the Oxford Hospitals Charitable

For peer review only - http://bmjopen.bmj.com/site/about/guidelines.xhtml

Fund and by the NIHR Oxford Biomedical Research Centre. The Diabetes Alliance for Research in England (DARE) study was funded by the Wellcome Trust and supported by the Exeter NIHR Clinical Research Facility. The MASTERMIND study was funded by the UK Medical Research Council (MR/N00633X/) and supported by the NIHR Exeter Clinical Research Facility. The PRIBA study was funded by the National Institute for Health Research (UK) (DRF-2010-03-72) and supported by the NIHR Exeter Clinical Research Facility.

T.J.M is a National Institute for Health Research Senior Clinical Senior Lecturer. R.A.O is supported by a Diabetes UK Harry Keen Fellowship (16/0005529). . E.R.P. is a Wellcome Trust New Investigator (102820/Z/13/Z). A.T.H and B.M.S are supported by the NIHR Exeter Clinical Research Facility. A.T.H is a Wellcome Trust Senior Investigator and NIHR Senior Investigator. A.G.J is supported by an NIHR Clinician Scientist award (CS-2015-15-018).

The views expressed in this article are those of the authors do not necessarily represent those of the National Institute for Health Research, the National Health Service or the Department of Health.

The study sponsor was not involved in the design of the study; the collection, analysis, and interpretation of data; writing the report; or the decision to submit the report for publication

Copyright/license for publication:

The Corresponding Author has the right to grant on behalf of all authors and does grant on behalf of all authors, a worldwide licence to the Publishers and its licensees in perpetuity, in all forms, formats and media (whether known now or created in the future), to i) publish, reproduce, distribute, display and store the Contribution, ii)

For peer review only

translate the Contribution into other languages, create adaptations, reprints, include within collections and create summaries, extracts and/or, abstracts of the Contribution, iii) create any other derivative work(s) based on the Contribution, iv) to exploit all subsidiary rights in the Contribution, v) the inclusion of electronic links from the Contribution to third party material where-ever it may be located; and, vi) licence any third party to do any or all of the above.

Competing interests declaration:

All authors have completed the ICMJE uniform disclosure form at www.icmje.org/coi_disclosure.pdf and declare: no support from any organisation for the submitted work; no financial relationships with any organisations that might have an interest in the submitted work in the previous three years; no other relationships or activities that could appear to have influenced the submitted work.

Ethics approval:

Not required

Data Availability

Data from the Exeter cohorts included in this research is held by the Peninsula Research Bank, managed by the NIHR Exeter Clinical Research Facility. Guidance for applying to use the Peninsula Research Bank resource are given on the following website: https://exetercrfnihr.org/about/exeter-10000-prb/

For peer review only

References

[1] American Diabetes Association (2018) 2. Classification and Diagnosis of Diabetes: Standards of Medical Care in Diabetes—2018. Diabetes care 41: S13 - S27
[2] National Institute for Health and Care Excellence (2015) Type 1 diabetes in adults: diagnosis and management (NICE guideline NG17). Available from https://www.nice.org.uk/guidance/ng17, accessed 14/08/2018
[3] National Institute for Health and Care Excellence (2015) Type 2 diabetes in adults: management (NICE guideline NG28). Available from https://www.nice.org.uk/guidance/ng28, accessed 14/08/2018
[4] American Diabetes Association (2018) 8. Pharmacologic Approaches to Glycemic Treatment: Standards of Medical Care in Diabetes—2018. Diabetes care 41: S73 - S85
[5] DeWitt DE, Hirsch IB (2003) Outpatient insulin therapy in type 1 and type 2 diabetes mellitus: Scientific review. JAMA 289: 2254-2264
[6] Frandsen CS, Dejgaard TF, Madsbad S (2016) Non-insulin drugs to treat hyperglycaemia in type 1 diabetes mellitus. The Lancet Diabetes & Endocrinology 4: 766-780
[7] (1998) Effect of intensive therapy on residual β-cell function in patients with type 1 diabetes in the diabetes control and complications trial: A randomized, controlled trial. Annals of Internal Medicine 128: 517-523
[8] Inzucchi SE, Bergenstal RM, Buse JB, et al. (2012) Management of hyperglycaemia in type 2 diabetes: a patient-centered approach. Position statement of the American Diabetes Association (ADA) and the European Association for the Study of Diabetes (EASD). Diabetologia 55: 1577-1596
[9] Riddle MC (2008) Combined Therapy With Insulin Plus Oral Agents: Is There Any Advantage? Diabetes care 31: S125
[10] Farmer A, Fox R (2011) Diagnosis, classification, and treatment of diabetes. BMJ 342
[11] Hope SV, Knight BA, Shields BM, et al. (2018) Random non-fasting C-peptide testing can identify patients with insulin-treated type 2 diabetes at high risk of hypoglycaemia. Diabetologia 61: 66-74
[12] Stone MA, Camosso-Stefinovic J, Wilkinson J, De Lusignan S, Hattersley AT, Khunti K (2010) Incorrect and incomplete coding and classification of diabetes: a systematic review. Diabetic Medicine 27: 491-497
[13] World Health Organization (2006) Definition and diagnosis of diabetes mellitus and intermediate hyperglycaemia: report of a WHO/IDF consultation. In:
[14] Shields BM, Peters JL, Cooper C, et al. (2015) Can clinical features be used to differentiate type 1 from type 2 diabetes? A systematic review of the literature. BMJ open 5
[15] Rosenbloom AL, Joe JR, Young RS, Winter WE (1999) Emerging epidemic of type 2 diabetes in youth. Diabetes care 22: 345
[16] Thomas NJ, Jones SE, Weedon MN, Shields BM, Oram RA, Hattersley AT (2018) Frequency and phenotype of type 1 diabetes in the first six decades of life: a cross-sectional, genetically stratified survival analysis from UK Biobank. The Lancet Diabetes & Endocrinology 6: 122-129
[17] Niskanen LK, Tuomi T, Karjalainen J, Groop LC, Uusitupa MIJ (1995) GAD Antibodies in NIDDM: Ten-year follow-up from the diagnosis. Diabetes care 18: 1557

For peer review only - http://bmjopen.bmj.com/site/about/guidelines.xhtml

[18] Oram RA, Patel K, Hill A, et al. (2016) A Type 1 diabetes genetic risk score can aid discrimination between Type 1 and Type 2 diabetes in young adults. Diabetes care 39: 337-344
[19] Jones AG, Besser REJ, Shields BM, et al. (2012) Assessment of endogenous insulin secretion in insulin treated diabetes predicts postprandial glucose and treatment response to prandial insulin. BMC Endocrine Disorders 12: 6
[20] Steffes MW, Sibley S, Jackson M, Thomas W (2003) Beta-cell function and the development of diabetes-related complications in the diabetes control and complications trial. Diabetes care 26: 832-836
[21] Jones AG, Hattersley AT (2013) The clinical utility of C-peptide measurement in the care of patients with diabetes. Diabetic Medicine 30: 803-817
[22] Jones AG, McDonald TJ, Shields BM, et al. (2016) Markers of beta cell failure predict poor glycemic response to GLP-1 receptor agonist therapy in type 2 diabetes. Diabetes care 39: 250-257
[23] Chow LS, Chen H, Miller ME, Marcovina SM, Seaquist ER (2015) Biomarkers related to severe hypoglycaemia and lack of good glycaemic control in ACCORD. Diabetologia 58: 1160-1166
[24] Thunander M, Törn C, Petersson C, Ossiansson B, Fornander J, Landin-Olsson M (2012) Levels of C-peptide, body mass index and age, and their usefulness in classification of diabetes in relation to autoimmunity, in adults with newly diagnosed diabetes in Kronoberg, Sweden. European journal of endocrinology 166: 1021-1029
[25] Collins GS, Mallett S, Omar O, Yu L-M (2011) Developing risk prediction models for type 2 diabetes: a systematic review of methodology and reporting. BMC Medicine 9: 103-103
[26] Shields BM, McDonald TJ, Ellard S, Campbell MJ, Hyde C, Hattersley AT (2012) The development and validation of a clinical prediction model to determine the probability of MODY in patients with young-onset diabetes. Diabetologia 55: 1265-1272
[27] DiabetesGenes.org Diabetes Alliance for Research in England (DARE) Available from https://www.diabetesgenes.org/current-research/dare/, accessed 23 November 2017
[28] ClinicalTrials.gov RetroMASTER - Retrospective Cohort MRC ABPI STratification and Extreme Response Mechanism in Diabetes. Available from https://www.clinicaltrials.gov/ct2/show/NCT02109978
[29] ClinicalTrials.gov MASTERMIND - Understanding Individual Variation in Treatment Response in Type 2 Diabetes (Mastermind). Available from https://www.clinicaltrials.gov/ct2/show/NCT01847144?term=mastermind accessed 31 July 2018
[30] clinicaltrials.gov PROMASTER - PROspective Cohort MRC ABPI STratification and Extreme Response Mechanism in Diabetes (PROMASTER). Available from https://www.clinicaltrials.gov/ct2/show/NCT02105792?term=promaster&rank=1, accessed 31 July 2018
[31] Shields BM, Shepherd M, Hudson M, et al. (2017) Population-Based Assessment of a Biomarker-Based Screening Pathway to Aid Diagnosis of Monogenic Diabetes in Young-Onset Patients. Diabetes care 40: 1017-1025
[32] Woodmansey C, McGovern AP, McCullough KA, et al. (2017) Incidence, Demographics, and Clinical Characteristics of Diabetes of the Exocrine Pancreas (Type 3c): A Retrospective Cohort Study. Diabetes care

For peer review only - http://bmjopen.bmj.com/site/about/guidelines.xhtml

[33] Thanabalasingham G, Pal A, Selwood MP, et al. (2012) Systematic Assessment of Etiology in Adults With a Clinical Diagnosis of Young-Onset Type 2 Diabetes Is a Successful Strategy for Identifying Maturity-Onset Diabetes of the Young. Diabetes care 35: 1206-1212
[34] McDonald TJ, Colclough K, Brown R, et al. (2011) Islet autoantibodies can discriminate maturity-onset diabetes of the young (MODY) from Type 1 diabetes. Diabetic medicine : a journal of the British Diabetic Association 28: 1028-1033
[35] Hope SV, Knight BA, Shields BM, Hattersley AT, McDonald TJ, Jones AG (2016) Random non-fasting C-peptide: bringing robust assessment of endogenous insulin secretion to the clinic. Diabetic medicine : a journal of the British Diabetic Association 33: 1554-1558
[36] The Wellcome Trust Case Control C (2007) Genome-wide association study of 14,000 cases of seven common diseases and 3,000 shared controls. Nature 447: 661-678
[37] Harrell F (2015) Regression Modeling Strategies. Springer International Publishing, Switzerland
[38] Peduzzi P, Concato J, Kemper E, Holford TR, Feinstein AR (1996) A simulation study of the number of events per variable in logistic regression analysis. J Clin Epidemiol 49: 1373-1379
[39] van Smeden M, Moons KGM, de Groot JAH, et al. (2018) Sample size for binary logistic prediction models: Beyond events per variable criteria. Statistical Methods in Medical Research: 0962280218784726
[40] Steyerberg EW, Vickers AJ, Cook NR, et al. (2010) Assessing the performance of prediction models: a framework for some traditional and novel measures. Epidemiology (Cambridge, Mass) 21: 128-138
[41] Steyerberg EW, Vergouwe Y (2014) Towards better clinical prediction models: seven steps for development and an ABCD for validation. European Heart Journal 35: 1925-1931
[42] Scottish Diabetes Survey 2017 http://www.diabetesinscotland.org.uk/Publications/SDS%202017.pdf . Accessed 03/05/2019
[43] Tridgell DM, Spiekerman C, Wang RS, Greenbaum CJ (2011) Interaction of Onset and Duration of Diabetes on the Percent of GAD and IA-2 Antibody–Positive Subjects in the Type 1 Diabetes Genetics Consortium Database. Diabetes care 34: 988
[44] Hope SV, Wienand-Barnett S, Shepherd M, et al. (2016) Practical Classification Guidelines for Diabetes in patients treated with insulin: a cross-sectional study of the accuracy of diabetes diagnosis. The British journal of general practice : the journal of the Royal College of General Practitioners 66: e315-322

For peer review only

Figure Legends

Figure 1: Density plots for (A) age at diagnosis, (B) BMI and (D) T1D GRS. Stacked bar chart (C) showing percentages of participants (total n = 943 (stage 4 model development sample)) by actual type 1 diabetes outcome and GADA/IA-2A status. Dashed line shows the distribution for type 2 diabetes (T2D) (n = 815), solid line shows the distribution for type 1 diabetes (T1D) (n = 128) of participants included in the stage 4 model development.

Figure 2: Development sample validation results. Plots are the results from the validation of the models. First row (a and b): clinical features logistic regression model (n = 1,315). Second row (c and d): clinical features + GADA logistic regression model (n = 1,036). Third row (e and f): clinical features + GADA + IA-2A logistic regression model (n = 1,025). Fourth row (g and h): clinical features + GADA + IA-2A + T1D GRS logistic regression model (n =943). Plots (a), (c), (e), & (g) are ROC curves showing discrimination ability of the models. Plots (b), (d), (f) & (h) are boxplots of fitted model probabilities grouped by actual diabetes outcome.

Figure 3: External validation results. Plots on the first row (a, b, c) are the results from the external validation of the clinical features logistic regression model applied to participants in the YDX study (n = 582). The second row of plots (d, e, f) are the results from the external validation of the clinical features + GADA logistic regression model applied to participants in the YDX study (n = 549). Plots (a) & (d) are ROC curves showing discrimination ability of the models, dashed line represents the reference line. Plots (b) & (e) are calibration plots. Plots (c) & (f) are boxplots of fitted model probabilities grouped by actual diabetes outcome.

For peer review only - http://bmjopen.bmj.com/site/about/guidelines.xhtml

Tables

Clinical features (n = 1,352)						
	Probability (%) cut-off for classifying type 1 diabetes					
	10	30	50	70	90	12 (Youden's Index)
Sensitivity/specificity (%)	85/79	64/95	49/98	35/99	15/100	83/83
Accuracy (%)	80	90	91	90	89	83
Positive predictive value (PPV) (%)	38	64	79	83	90	42
Negative predictive value (NPV) (%)	97	95	93	91	89	97
Clinical features + GADA (n = 1,036)						
	Probability (%) cut-off for classifying type 1 diabetes					
	10	30	50	70	90	16 (Youden's Index)
Sensitivity/specificity (%)	90/88	80/96	66/97	52/99	31/100	86/92
Accuracy (%)	89	94	93	92	90	92
Positive predictive value (PPV) (%)	55	75	80	85	92	64
Negative predictive value (NPV) (%)	98	97	95	93	90	98
Clinical features + GADA + IA-2A (n = 1,025)						
	Probability (%) cut-off for classifying type 1 diabetes					
	10	30	50	70	90	12 (Youden's Index)
Sensitivity/specificity (%)	91/91	80/96	69/98	57/99	37/100	90/92
Accuracy (%)	91	94	94	93	92	92
Positive predictive value (PPV) (%)	59	75	81	85	92	62
Negative predictive value (NPV) (%)	99	97	96	94	92	98
Clinical features + GADA + IA-2A + T1D GRS (n = 943)						
	Probability (%) cut-off for classifying type 1 diabetes					
	10	30	50	70	90	14 (Youden's Index)
Sensitivity/specificity (%)	92/90	84/96	74/98	63/99	41/100	91/93
Accuracy (%)	90	95	94	94	92	93
Positive predictive value (PPV) (%)	59	78	83	88	93	67
Negative predictive value (NPV) (%)	99	98	96	94	92	99

Table 1: Model performance at different cut-offs for all four logistic regression models (development cohort). Positive and negative predictive values relate to type 1 diabetes.
Accuracy = (true positives + true negatives)/total number of participants.
Positive predictive value (PPV) =
[(sensitivity × prevalence)/[(sensitivity × prevalence) + ([1 – specificity] × [1−prevalence])].
Negative predictive value (NPV) =
[specificity × (1 − prevalence)]/[(specificity × [1 − prevalence]) + ([1 − sensitivity] × prevalence)].
Youden's Index - best trade-off between sensitivity and specificity (sensitivity+specificity – 1).

For peer review only

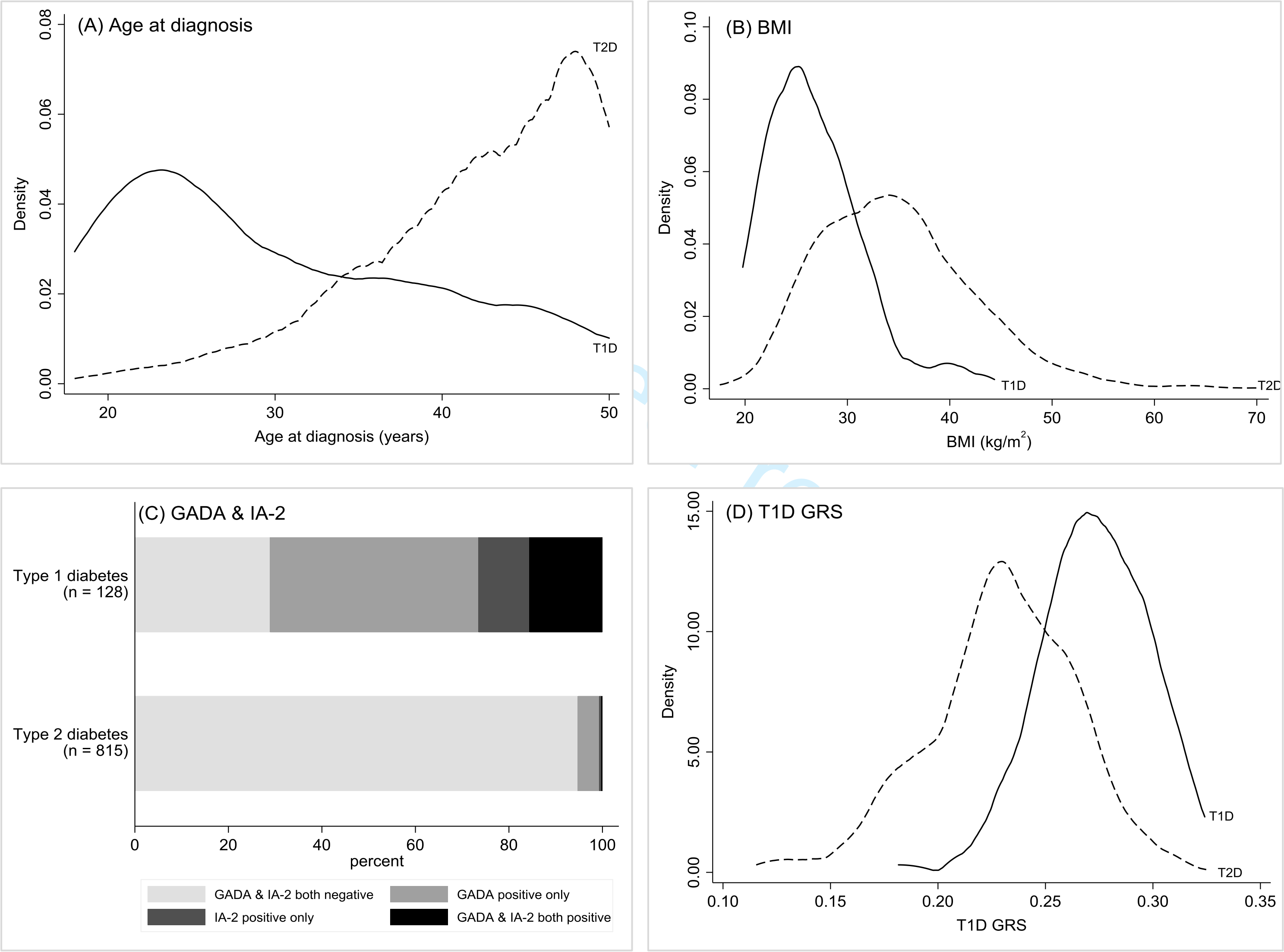
(A) Age at diagnosis
Density
0.00
0.02
0.04
0.06
0.08
T2D
T1D
20
30
40
50
Age at diagnosis (years)
(B) BMI
Density
0.00
0.02
0.04
0.06
0.08
0.10
T1D
T2D
20
30
40
50
60
70
BMI (kg/m²)
(C) GADA & IA-2
Type 1 diabetes
(n = 128)
Type 2 diabetes
(n = 815)
0
20
40
60
80
100
percent
GADA & IA-2 both negative
GADA positive only
IA-2 positive only
GADA & IA-2 both positive
(D) T1D GRS
Density
0.00
5.00
10.00
15.00
T1D
T2D
0.10
0.15
0.20
0.25
0.30
0.35
T1D GRS

Figure 1

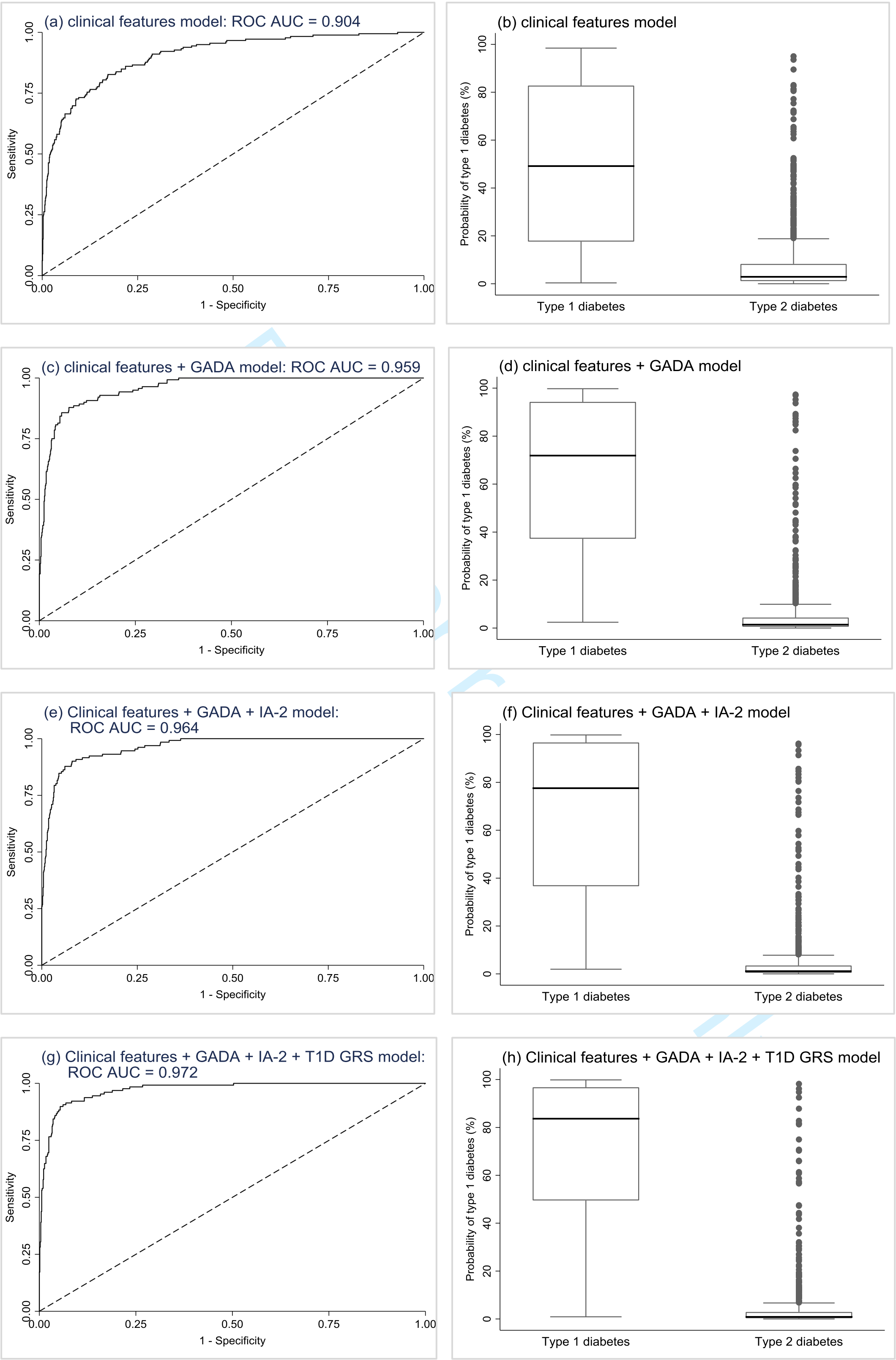
(a) clinical features model: ROC AUC = 0.904
Sensitivity
1 - Specificity
(b) clinical features model
Probability of type 1 diabetes (%)
Type 1 diabetes
Type 2 diabetes
(c) clinical features + GADA model: ROC AUC = 0.959
Sensitivity
1 - Specificity
(d) clinical features + GADA model
Probability of type 1 diabetes (%)
Type 1 diabetes
Type 2 diabetes
(e) Clinical features + GADA + IA-2 model:
ROC AUC = 0.964
Sensitivity
1 - Specificity
(f) Clinical features + GADA + IA-2 model
Probability of type 1 diabetes (%)
Type 1 diabetes
Type 2 diabetes
(g) Clinical features + GADA + IA-2 + T1D GRS model:
ROC AUC = 0.972
Sensitivity
1 - Specificity
(h) Clinical features + GADA + IA-2 + T1D GRS model
Probability of type 1 diabetes (%)
Type 1 diabetes
Type 2 diabetes

Figure 2

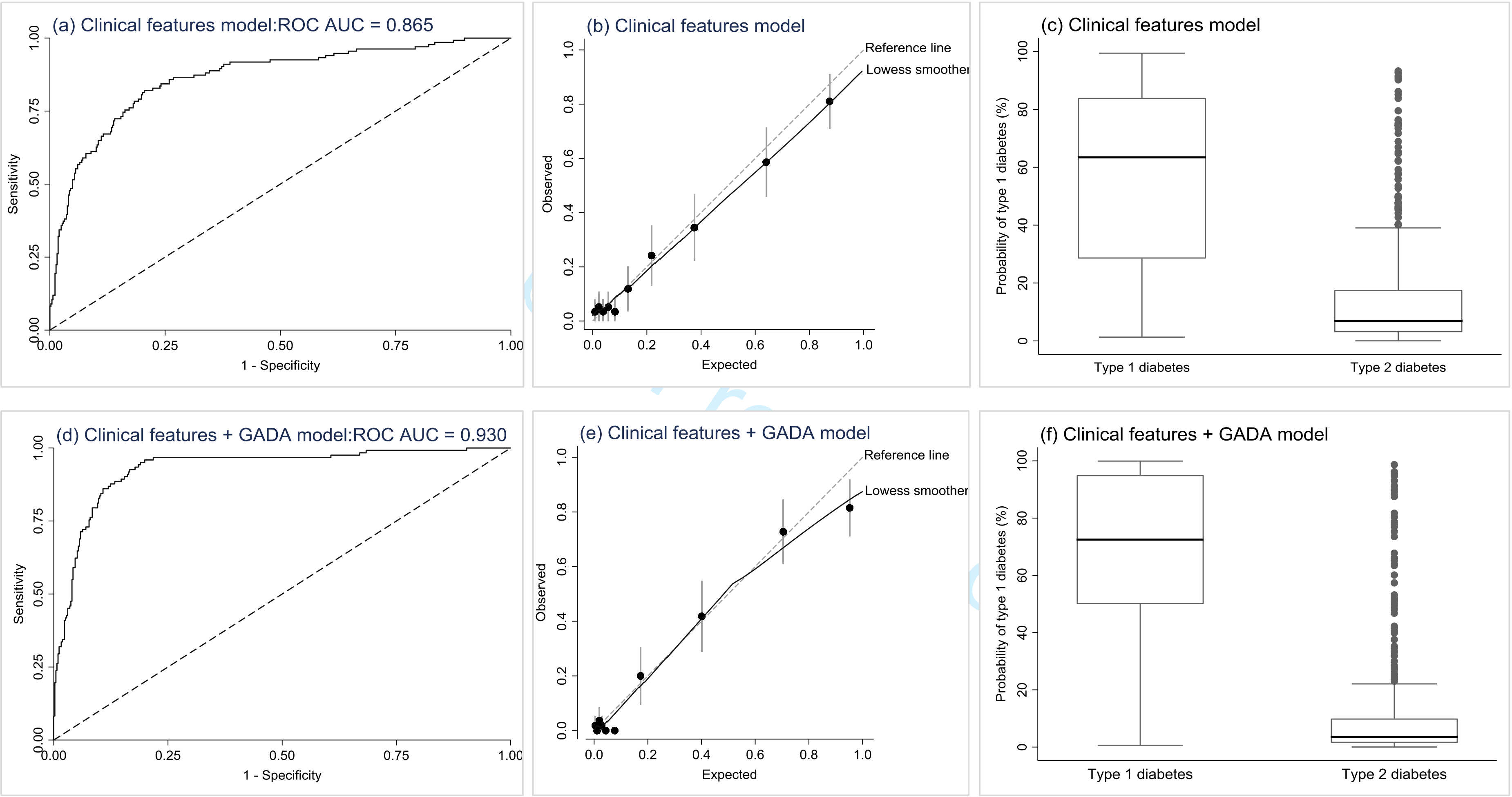
(a) Clinical features model:ROC AUC = 0.865
Sensitivity
1 - Specificity
0.00
0.25
0.50
0.75
1.00
(b) Clinical features model
Reference line
Lowess smoother
Observed
Expected
0.0
0.2
0.4
0.6
0.8
1.0
(c) Clinical features model
Probability of type 1 diabetes (%)
0
20
40
60
80
100
Type 1 diabetes
Type 2 diabetes
(d) Clinical features + GADA model:ROC AUC = 0.930
(e) Clinical features + GADA model
(f) Clinical features + GADA model

Figure 3

	DARE	PRIBA	MRC Pro/RetroMaster	MRC crossover
Included participants*	904	368	72	8
Data collection period	2007 to 2017	2011 to 2013	2013 to 2015	2013 to 2015
Study design	Cross-sectional	Longitudinal	Cross-sectional	Interventional Crossover
Setting	Primary and secondary care in eight diabetes research regions, England and retinal screening clinics.	Primary and secondary care in South West England	Primary and secondary care sites South West England, Tayside, Oxford, Glasgow, KCL and Newcastle, U.K.	Exeter and Tayside,U.K.
Inclusion criteria	Clinical diagnosis of diabetes (any type).	Clinical diagnosis of type 2 diabetes. Clinician determined requirement for DPP-IV inhibitor or GLP-1 analogue (HbA1c >7.5%)	Clinical diagnosis of type 2 diabetes non-insulin treated within 6 months of diagnosis. Participants were selected on the basis of rapid or slow progression to insulin therapy (<7, >7 years). Age 18-90 inclusive.	Clinical diagnosis of type 2 diabetes, currently treated with sulphonylurea tablets and no change in treatment in previous 3 months, Last HbA1c (within previous 12 months) ≥42 and ≤75 mmol/mol (6-9%). Age 19-79 inclusive.
Data collection	Clinical measurements and blood sample collected at visit. Ongoing biochemical data collected from pathology laboratories.	Clinical measurements and blood taken at initial visit. Follow up clinical measurements and blood collected at three and six months.	Clinical measures and fasting blood sample taken at visit.	MMT at baseline & MMT on each study drug visits. Three fasting blood collected at crossovers.

Supplementary Table 1: Cohort recruitment and data collection methods summary. *Included in the clinical features model stage 1 development.

SNP	Gene	Odds Ratio	Weight	Effect Allele
rs2187668, rs7454108	DR3/DR4	48.18	3.87	
	DR3/DR3	21.12	3.05	
	DR4/DR4	21.98	3.09	
	DR4/X	7.03	1.95	
	DR3/X	4.53	1.51	
rs1264813	HLA_A_24	1.54	0.43	T
rs2395029	HLA_B_5701	2.5	0.92	T
rs3129889	HLA_DRB1_15	14.88	2.70	A
rs2476601	PTPN22	1.96	0.67	A
rs689	INS	1.75	0.56	T
rs12722495	IL2RA	1.58	0.46	T
rs2292239	ERBB3	1.35	0.30	T
rs10509540	C10orf59	1.33	0.29	T
rs4948088	COBL	1.3	0.26	C
rs7202877		1.28	0.25	G
rs12708716	CLEC16A	1.23	0.21	A
rs3087243	CTLA4	1.22	0.20	G
rs1893217	PTPN2	1.2	0.18	G
rs11594656	IL2RA	1.19	0.17	T
rs3024505	IL10	1.19	0.17	G
rs9388489	C6orf173	1.17	0.16	G
rs1465788		1.16	0.15	C
rs1990760	IFIH1	1.16	0.15	T
rs3825932	CTSH	1.16	0.15	C
rs425105		1.16	0.15	T
rs763361	CD226	1.16	0.15	T
rs4788084	IL27	1.16	0.15	C
rs17574546		1.14	0.13	C
rs11755527	BACH2	1.13	0.12	G
rs3788013	UBASH3A	1.13	0.12	A
rs2069762	IL2	1.12	0.11	A
rs2281808		1.11	0.10	C
rs5753037		1.1	0.10	T

Supplementary Table 2: Type 1 diabetes SNPs included in the genetic risk score with weights. Effect allele is the risk increasing allele on the positive strand.

For peer review only

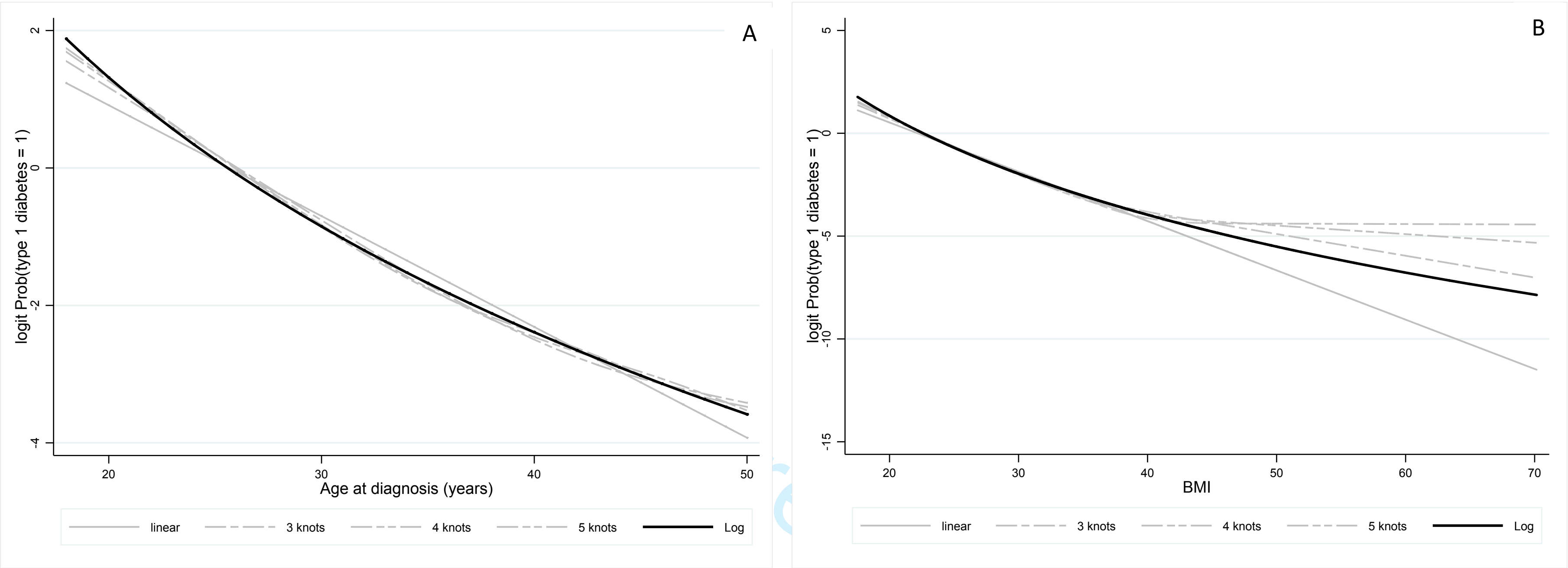

Supplementary Figure 1: Relationship between age at diagnosis (A) and BMI (B) and response modelled using restricted cubic splines (k = 3, 4 and 5) and a simple log transformation. Age at diagnosis and BMI did not predict linearly, the graphs of fitted splines and log transformation suggested that a simple log transformation was sufficient to induce linearity in both variables.

For peer review only - http://bmjopen.bmj.com/site/about/guidelines.xhtml

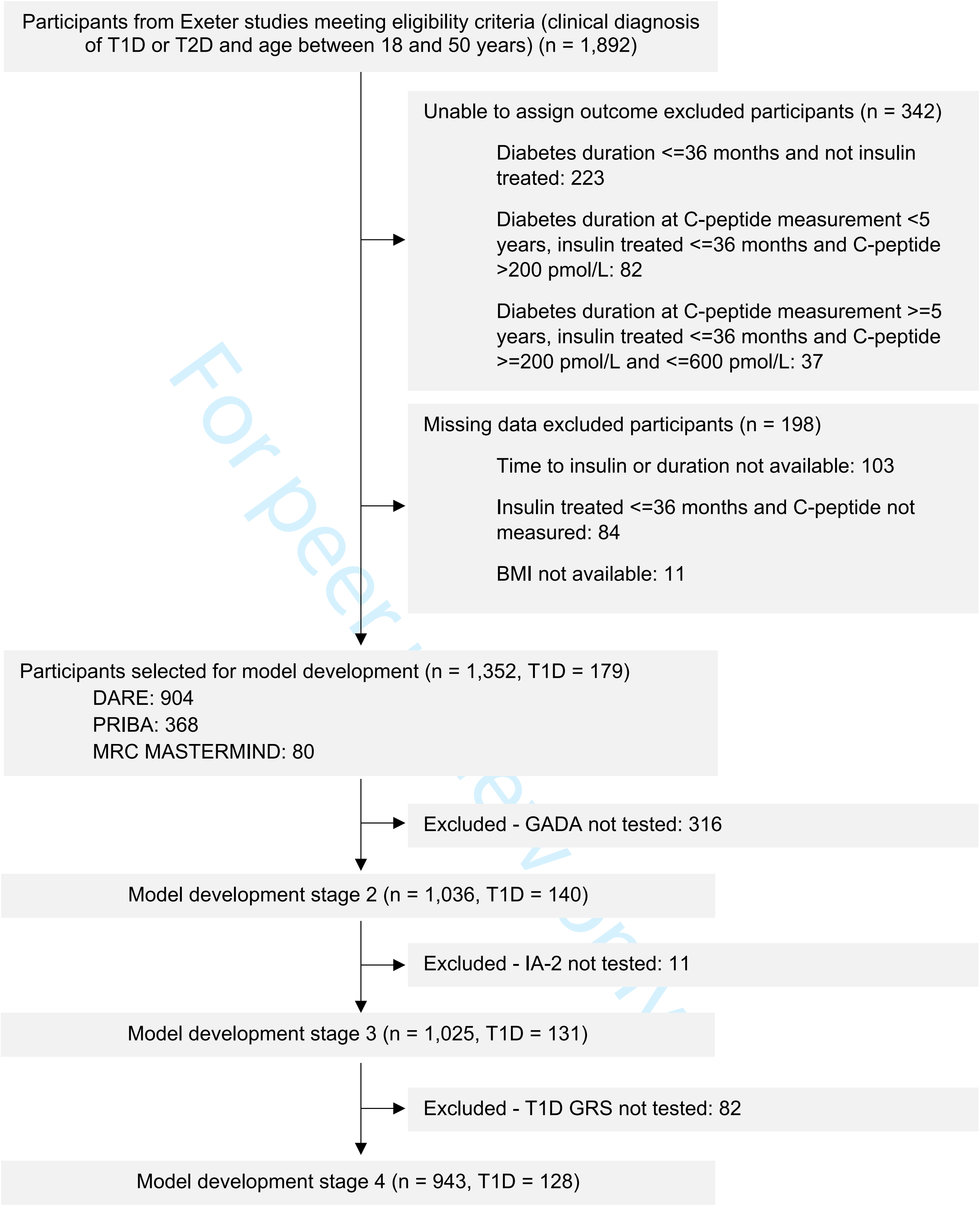

Supplementary Figure 2: Flow diagram of participants through the model development stages. T1D: type 1 diabetes, T2D: type 2 diabetes

For peer review only - http://bmjopen.bmj.com/site/about/guidelines.xhtml

	Model 1 development n = 1,352	Model 2 development n = 1,036	Model 3 development n = 1,025	Model 4 development n = 943
**Characteristic**				
Sex (% Male)	59%	59%	59%	59%
Age at diagnosis (years)*	40 [39, 41]	40 [39, 40]	40 [39, 40]	40 [39, 40]
Age at diagnosis (years) min, max	18, 50	18, 50	18, 50	18, 50
BMI (kg/m^2)*†	33 [32, 33]	33 [32, 33]	33 [32, 33]	33 [32, 33]
BMI (kg/m^2)*† min, max	17.5, 70.2	17.5, 70.2	17.5, 70.2	17.5, 70.2
Duration of diabetes (years)	13 (8, 20)	13 (8, 20)	13 (8, 20)	13 (8, 20)
Type 1 diabetes	13%	14%	13%	14%
HbA1c (%)†	8.2 (7.1, 9.6)	8.3 (7.3, 9.8)	8.3 (7.3, 9.8)	8.2 (7.2, 9.7)
HbA1c (mmol/mol)†	66 (54, 81)	67 (56, 84)	67 (56, 84)	66 (55, 83)
GADA positive (%)	-	12%	12%	12%
IA-2 positive (%)	-	-	4%	4%
T1D GRS	-	-	-	0.24 (0.22, 0.26)
T1D GRS centile	-	-	-	5.8 (1.2, 23.7)
T1D GRS min, max	-	-	-	0.12, 0.32

Supplementary Table 3: Characteristics of the Exeter, U.K. study participants included at each model development stage. Model 1 – Clinical features (Age at diagnosis & BMI), Model 2 – Clinical features + GADA, Model 3 - Clinical features + GADA + IA-2, Model 4 - Clinical features + GADA + IA-2 + T1D GRS. Median (IQR) or % or *Geometric mean [95% CI] for transformed variables. †Measured at recruitment (median 13 years post diagnosis). Minimum and maximum values for each continuous predictor variable used in the models

For peer review only - http://bmjopen.bmj.com/site/about/guidelines.xhtml

	Model 4 development n = 943	Model 4 development exclusions n = 409	p value for comparison
**Characteristic**			
Sex (% Male)	59%	60%	>0.1
Age at diagnosis (years)*	40 [39, 40]	41 [40, 42]	0.04
BMI (kg/m^2)*†	33 [32, 33]	33 [32, 33]	> 0.1
Duration of diabetes (years)	13 (8, 20)	13 (7, 20)	> 0.1
Type 1 diabetes	14%	12%	> 0.1
HbA1c (%)†	8.2 (7.2, 9.7)	8.0 (6.9, 9.3)	0.009
HbA1c (mmol/mol)†	66 (55, 83)	64 (52, 78)	0.009

Supplementary Table 4: Comparison of characteristics for participants included in the model 4 development and participants included in model 1 development but excluded from model 4. Median (IQR) or % or *Geometric mean [95% CI] for transformed variables. †Measured at recruitment (median 13 years post diagnosis).

For peer review only

For peer review only - http://bmjopen.bmj.com/site/about/guidelines.xhtml

Performance parameter	Development sample validation	Internal validation (bootstrap 500) Apparent (SD)	test (SD)	Optimism
**Clinical features model (n = 1,352)**				
ROC [95% CI]	0.90 [0.88, 0.93]	0.9056 (0.013)	0.9038 (0.0005)	0.0018
Calibration-in-the-large	0	0.0000 (0.000)	0.0003 (0.1072)	-0.0003
Calibration slope (b_L)	1	1.0000 (0.000)	0.9977 (0.0678)	0.0023
Brier Score	0.07 (p = 0.50)	-	-	-
Hosmer-Lemeshow	p = 0.95	-	-	-
Jack-knife cross validation†	0.09	-	-	-
**Clinical features + GADA model (n = 1,036)**				
ROC [95% CI]	0.96 [0.95, 0.97]	0.9595 (0.0070)	0.9586 (0.0010)	0.0009
Calibration-in-the-large	0	0.0000 (0.0000)	-0.0019 (0.1472)	0.0019
Calibration slope (b_L)	1	1.0000 (0.0000)	0.9850 (0.0787)	0.015
Brier Score	0.05 (p = 0.35)	-	-	-
Hosmer-Lemeshow	p = 0.39	-	-	-
Jack-knife cross validation†	0.07	-	-	-
**Clinical features + GADA + IA-2 model (n = 1,025)**				
ROC [95% CI]	0.96 [0.95, 0.98]	0.9622 (0.007)	0.9633 (0.0015)	0.0011
Calibration-in-the-large	0	0.0000 (0.000)	0.0055 (0.1567)	-0.0055
Calibration slope (b_L)	1	1.0000 (0.000)	0.9780 (0.0707)	0.022
Brier Score	0.04 (p = 0.31)	-	-	-
Hosmer-Lemeshow	p = 0.14	-	-	-
Jack-knife cross validation †	0.06	-	-	-
**Clinical features + GADA + IA-2 + T1D GRS model (n = 943)**				
ROC [95% CI]	0.97 [0.96, 0.98]	0.9718 (0.0060)	0.9710 (0.0006)	0.0008
Calibration-in-the-large	0	0.0000 (0.0000)	0.0084 (0.1675)	-0.0084
Calibration slope (b_L)	1	1.0000 (0.0000)	0.9880 (0.0810)	0.0124
Brier Score	0.04 (p = 0.35)	-	-	-
Hosmer-Lemeshow	p = 0.84	-	-	-
Jack-knife cross validation †	0.06	-	-	-

Supplementary Table 5: Model performance results for the internal validation performed at each development stage. * P value for Brier score is Spiegelhalter's z-test used to evaluate the calibration component of the Brier score, significant p-values indicate poor calibration. †Result reported as raw cross-validation estimate of prediction error with misclassification cost function (cut-off 0.5). cv.glm function in R version 3.3.3.

For peer review only - http://bmjopen.bmj.com/site/about/guidelines.xhtml

Model	LR	Adequacy
Clinical features	324.7 (df 2)	0.67
Clinical features + GADA	418.7 (df 3)	0.87
Clinical features + GADA + IA-2	447.6 (df 5)	0.93
Clinical features + GADA + IA-2 + T1D GRS	481.8 (df 6)	1.00

Supplementary Table 6: Unitless index of adequacy is the proportion of log likelihood explained by each model stage with reference to the end model containing all predictors. Based on replica models developed using stage 4 development sample (n = 943).

Model comparison	Likelihood Ratio test	Net Reclassification Improvement	Integrated Discrimination Improvement
Adding GADA to Clinical features model	LR chi2(1) = 94.02 p <0.001	0.12, p = 0.01	0.13, p < 0.001
Adding IA-2 to Clinical features + GADA model	LR chi2 (2) = 28.82 p < 0.001	0.14, p = 0.004	0.15, p < 0.001
Adding T1D GRS to Clinical features + GADA + IA-2 model	LR chi2 (2) = 34.20 p < 0.001	0.06, p = 0.04	0.06, p < 0.001

Supplementary Table 7: Model fit comparisons of nested models developed using stage 4 development sample (n = 943). Null hypothesis for Likelihood Ratio test: Additional predictor(s) has no predictive information. Net Reclassification Improvement calculated using 50% classification cut-off.

Model	Clinical features ROC AUC	Clinical features + GADA ROC AUC	Clinical features + GADA + IA-2 ROC AUC
Development sample 1 (n = 1,352)	0.90 [0.88, 0.93]	-	-
Development sample 2 (n = 1,036)	-	0.96 [0.95, 0.97]	-
Development sample 3 (n = 1,025)	-	-	0.96 [0.95, 0.98]
**Development sample 4 (n = 943)**	**0.91 [0.89, 0.94]**	**0.96 [0.94, 0.97]**	**0.96 [0.95, 0.98]**

Supplementary Table 8: Model performance comparison with replica models developed using stage 4 development sample (n = 943).

For peer review only - http://bmjopen.bmj.com/site/about/guidelines.xhtml

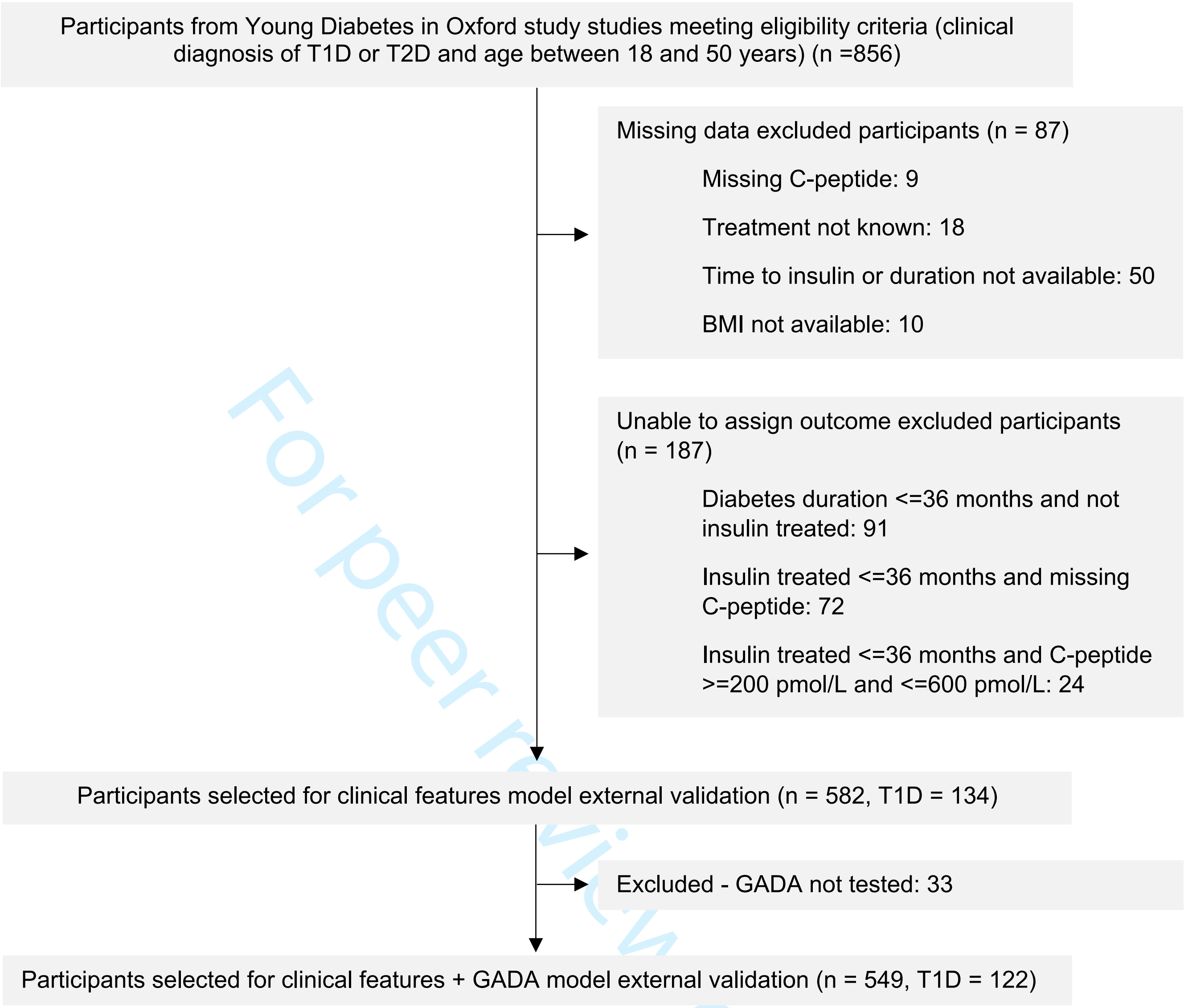

Supplementary Figure 3: Flow diagram of participants through the model external validation stages. T1D: type 1 diabetes, T2D: type 2 diabetes

For peer review only

For peer review only - http://bmjopen.bmj.com/site/about/guidelines.xhtml

Characteristic	Model 1 development n = 1,352	Model 1 validation n = 582	comparison p value	Model 2 development n = 1,036	Model 2 validation n = 549	comparison p value
Sex (% Male)	59%	61%	>0.1	59%	61%	> 0.1
Age at diagnosis (years)	43 (36, 48)	37 (30, 41)	<0.001	43 (36, 48)	37 (30, 41)	< 0.001
BMI (kg/m^2)*	33 (28, 38)	31 (27, 36)	<0.001	33 (28, 38)	31 (27, 36)	< 0.001
Duration of diabetes (years)*	13 (8, 20)	14 (8, 23)	0.03	13 (8, 20)	13 (8, 23)	> 0.1
Type 1 diabetes	13%	23%	<0.001	14%	22%	< 0.001
HbA1c (%)*	8.2 (7.1, 9.6)	8.1 (7.2, 9.3)	>0.1	8.3 (7.3, 9.8)	8.1 (7.2, 9.4)	0.08
HbA1c (mmol/mol)*	66 (54, 81)	65 (55, 78)	>0.1	67 (56, 84)	65 (55, 79)	0.08
GADA (% positive)	-	-	-	12%	20%	< 0.001

Supplementary Table 9: Baseline characteristics comparison of the development and validation data sets for: Model 1 – Clinical features (Age at diagnosis & BMI) and Model 2 – Clinical features + GADA. *Measured at recruitment (median 13 years and 14 years post diagnosis in development data sets and validation data sets). Kruskal-Wallis used for comparison testing continuous variables, chi-square for categorical variables.

For peer review only - http://bmjopen.bmj.com/site/about/guidelines.xhtml

For peer review only

Performance parameter	External validation
**Clinical features model (n = 582)**	
ROC [95% CI]	0.86 [0.83, 0.90]
Expected/Observed	1.06
Calibration-in-the-large ($a|b_L$=1)	-0.14
Calibration slope (b_L)	0.85
Overall misclassification	-0.14 p = 0.05
Brier Score*	0.11 (p = 0.14)
**Clinical features + GADA model (n = 549)**	
ROC [95% CI]	0.93 [0.90, 0.96]
Expected/Observed	1.08
Calibration-in-the-large ($a|b_L$=1)	-0.23
Calibration slope (b_L)	0.90
Overall misclassification	-0.10 p > 0.1
Brier Score*	0.08 (p = 0.29)

Supplementary Table 10: Model performance results for the external validation of the clinical features and clinical features+ GADA models. * P value for Brier score is Spiegelhalter's z-test used to evaluate the calibration component of the Brier score, significant p-values indicate poor calibration.

For peer review only

Clinical features	Development (n = 1,352)					Validation (n = 582)				
	Probability cut-off for classifying type 1 diabetes					Probability cut-off for classifying type 1diabletes				
	10	30	50	70	90	10	30	50	70	90
Sensitivity/specificity (%)	85/79	64/95	49/98	35/99	15/100	91/62	73/85	59/93	45/96	13/99
Accuracy (%)	80	90	91	90	89	69	82	85	84	79
Positive predictive value (PPV) (%)	38	64	79	83	90	42	59	71	77	77
Negative predictive value (NPV) (%)	97	95	93	91	89	96	91	88	85	79

Clinical features + GADA	Development (n = 1,036)					Validation (n = 549)				
	Probability cut-off for classifying type 1 diabetes					Probability cut-off for classifying type 1diabletes				
	10	30	50	70	90	10	30	50	70	90
Sensitivity/specificity (%)	90/88	80/96	66/97	52/99	31/100	97/75	86/89	75/93	55/96	42/97
Accuracy (%)	89	94	93	92	90	80	88	88	87	85
Positive predictive value (PPV) (%)	55	75	80	85	92	53	69	73	80	81
Negative predictive value (NPV) (%)	98	97	95	93	90	99	96	93	88	85

Supplementary Table 11: Classification table comparing the development and validation samples at different cut-offs for probability of type 1 diabetes using the clinical features and clinical features + GADA logistic regression models.

Accuracy = (true positives + true negatives)/total number of participants.

Positive predictive value (PPV) = (sensitivity × prevalence)/[(sensitivity × prevalence) + ([1 – specificity] × [1−prevalence])].

Negative predictive value (NPV) = [specificity × (1 − prevalence)]/[(specificity × [1 − prevalence]) + ([1 − sensitivity] × prevalence)].

PPV and NPV assume prevalence for type 1 diabetes: Clinical features model – 13% (development) and 23% (validation), Clinical features + GADA model - 14% (development) and 22% (validation).

For peer review only

For peer review only - http://bmjopen.bmj.com/site/about/guidelines.xhtml

Age at diagnosis (years)	BMI (kg/m^2)	GADA positive	C-Peptide (PmolL)*	Insulin Treated	Time to insulin (months)	Duration at screening (years)†	Actual diabetes outcome	Probability of type 1 diabetes‡ (%)
18	26	0	775	1	Immediate	15	Type 2 diabetes	80
21	23	0	868	1	Immediate	10	Type 2 diabetes	82
27	29	1	-	0	-	3	Type 2 diabetes	88
38	22	1	550	1	48	10	Type 2 diabetes	88
36	22	1	175	1	72	12	Type 2 diabetes	89
23	32	1	25	1	48	29	Type 2 diabetes	90
30	25	1	25	1	36	30	Type 2 diabetes	91
29	25	1	225	1	48	12	Type 2 diabetes	93
23	28	1	50	1	120	28	Type 2 diabetes	95
33	21	1	65	1	96	47	Type 2 diabetes	95
34	20	1	25	1	120	22	Type 2 diabetes	96
23	22	1	-	0	-	3	Type 2 diabetes	99

Supplementary table 12: Characteristics of participants with probability of Type 1 diabetes > 80% but with type 2 diabetes actual outcome *Non fasting equivalent, measured > 5 years post diagnosis (unless < 200 PmolL prior to 5 years). † C-peptide measured at single screening visit. ‡Clinical features + GADA model applied to participants in the YDX study.

For peer review only

For peer review only - http://bmjopen.bmj.com/site/about/guidelines.xhtml

Age at diagnosis (years)	BMI (kg/m^2)	GADA positive	C-Peptide (PmolL)*	Insulin Treated	Time to insulin (months)	Duration at screening (years)†	Actual diabetes outcome	Probability of type 1 diabetes (%)‡
41	40	0	50	1	12	41	Type 1 diabetes	0.6
40	34	0	198	1	12	34	Type 1 diabetes	1.8
43	31	0	125	1	3	1	Type 1 diabetes	2.1
39	33	0	25	1	24	17	Type 1 diabetes	2.5
38	25	0	68	1	Immediate	19	Type 1 diabetes	12.7
39	40	1	50	1	Immediate	16	Type 1 diabetes	14.9

Supplementary table 13: Characteristics of participants with probability of Type 1 diabetes < 16% (Youden's Index cut-off) but with type 1 diabetes actual outcome *Non-fasting equivalent, measured > 5 years post diagnosis (unless < 200 pmolL prior to 5 years). † C-peptide measured at single screening visit. ‡Clinical features + GADA model applied to participants in the YDX study.

Model	ROC [95% CI]	Jack-knife cross validation *
Clinical features + IA-2	0.93 [0.90, 0.95]	0.07
Clinical features + T1D GRS	0.93 [0.90, 0.95]	0.08
Clinical features + IA-2 + T1D GRS	0.95 [0.93, 0.97]	0.06
Clinical features + GADA + T1D GRS	0.97 [0.96, 0.98]	0.07

Supplementary table 14: Model performance results for the four additional models in the online calculator. * Result reported as raw cross-validation estimate of prediction error with misclassification cost function (cut-off 0.5). cv.glm function in R version 3.3.3.

For peer review only

Included	β (SE)	Odds Ratio [95% CI]	p value
Constant (intercept)	37.94 (2.67)	-	-
Age at diagnosis (years) *	-5.09 (0.41)	0.006 [0.003, 0.014]	<0.001
BMI (kg/m^2) *	-6.34 (0. 60)	0.002 [0.001, 0.005]	<0.001

Supplementary Table 15: Clinical features logistic regression model (model 1). * Log transformed. Linear Predictor mean -2.96, sd 1.98

Included	β (SE)	Odds Ratio [95% CI]	p value
Constant (intercept)	-0.98 (0.19)	-	-
Model 1 linear predictor	0.94 (0.08)	2.57 (2.18, 3.03)	< 0.001
GADA positive	3.11 (0.32)	22.50 (12.13, 41.76)	< 0.001

Supplementary Table 16: Clinical features + GADA logistic regression model (model 2). Linear Predictor mean -3.37, sd 2.53

For peer review only

Included	β (SE)	Odds Ratio [95% CI]	p value
Constant (intercept)	-1.28 (0.21)	-	
Model 1 linear predictor	0.92 (0.09)	2.50 [2.10, 2.98]	< 0.001
Antibody status - GADA positive only	3.08 (0.35)	21.81 [11.06, 43.02]	< 0.001
Antibody status - IA-2 positive only	3.49 (0.78)	32.93 [7.11, 152.64]	< 0.001
Antibody status - GADA & IA-2 both positive	4.35 (0.75)	77.53 [17.74, 338.84]	< 0.001

Supplementary Table 17: Clinical features + GADA + IA-2 logistic regression model (model 3). Linear Predictor mean -3.55, sd 2.58

Included	β (SE)	Odds Ratio [95% CI]	p value
Constant (intercept)	-0.67 (0.24)	-	-
Model 3 linear predictor	0.88 (0.08)	2.40 [2.06, 2.80]	< 0.001
T1D GRS (per 1 SD change)	1.08 (0.21)	2.93 [1.96, 4.39]	< 0.001

Supplementary Table 18: Clinical features + GADA + IA-2 + T1D GRS logistic regression model (model 4). T1D GRS standardized using mean 0.2356997, sd 0.0363499. Linear Predictor mean -3.74, sd 2.89.

Included	β (SE)	Odds Ratio [95% CI]	p value
Constant (intercept)	-0.36 (0.17)	-	-
Model 1 linear predictor	0.99 (0.08)	2.70 [2.30, 3.16]	< 0.001
IA-2 positive	3.19 (0.55)	24.39 [8.27, 71.92]	< 0.001

Supplementary Table 19: Clinical features + IA-2 logistic regression model. Linear Predictor mean -3.17, SD 2.28

For peer review only

For peer review only - http://bmjopen.bmj.com/site/about/guidelines.xhtml

Included	β (SE)	Odds Ratio [95% CI]	p value
Constant (intercept)	-0.65 (0.18)	-	-
Model 1 linear predictor	0.87 (0.07)	2.39 [2.09, 2.74]	< 0.001
T1D GRS (per 1 SD change)	1.22 (0.15)	3.38 [2.51, 4.54]	< 0.001

Supplementary Table 20: Clinical features + T1D GRS logistic regression model. T1D GRS standardized using mean 0.2360879, sd 0.0358468. Linear Predictor mean -3.180108, sd 2.401089.

Included	β (SE)	Odds Ratio [95% CI]	p value
Constant (intercept)	-1.12 (0.23)	-	-
Model 1 linear predictor	0.87 (0.09)	2.40 [2.02, 2.84]	< 0.001
T1D GRS (per 1 SD change)	1.36 (0.20)	3.89 [2.64, 5.74]	< 0.001
IA-2 positive	2.95 (0.65)	19.17 [5.33, 68.81]	< 0.001

Supplementary Table 21: Clinical features + IA-2 + T1D GRS logistic regression model. T1D GRS standardized using mean 0.235673, sd 0.0363399. Linear Predictor mean -3.537275, sd 2.79395.

Included	β (SE)	Odds Ratio [95% CI]	p value
Constant (intercept)	-1.50 (0.24)	-	-
Model 1 linear predictor	0.85 (0.09)	2.33 [1.97, 2.76]	< 0.001
T1D GRS (per 1 SD change)	1.12 (0.20)	3.05 [2.09, 4.46]	< 0.001
GADA positive	2.63 (0.34)	13.89 [7.17, 26.90]	< 0.001

Supplementary Table 22: Clinical features + GADA + T1D GRS logistic regression model. T1D GRS standardized using mean 0.2359649, sd 0.0363407. Linear Predictor mean - 3.596086, sd 2.868552.

For peer review only - http://bmjopen.bmj.com/site/about/guidelines.xhtml

Model	Linear predictor (lp) regression equation*
Clinical features	37.94 + (-5.09 * log(age)) + (-6.34 * log(BMI))
Clinical features + GADA†	34.8057844720 + (-4.801441792 * log (Age)) + (-5.980577792 * log(BMI)) + (2.937107976 * GADA†)
Clinical features + GADA + IA-2	33.49649577 + (-4.665598345 * Log(Age)) + (-5.81137397 * Log(BMI)) + (3.082366 * AntiStatus1‡) + (3.494462 * AntiStatus2‡) + (4.350717 * AntiStatus3‡)
Clinical features + GADA + IA-2 + T1D GRS	21.57649882 + (-4.086215772 * Log(Age)) + (-5.096252172 * Log(BMI)) + (2.702010666 * AntiStatus1‡) + (3.063255174 * AntiStatus2‡) + (3.813850704 * AntiStatus3‡) + (30.11052 * T1D GRS)
Clinical features + IA-2	37.26905033 + (3.194096 * IA-2†) + (-5.047657308 * Log(Age)) + (-6.287258808 * Log(BMI))
Clinical features + T1D GRS	24.46138054 + (-4.443506884 * Log(Age)) + (-5.534741384 *Log(BMI)) + (33.93968 * T1D GRS)
Clinical features + IA-2 + T1D GRS	23.2151829 +(2.953142 * IA-2†) + (-4.446784844 *Log(Age))+(-5.538824344 * Log(BMI)) + (37.40205 * T1D GRS)
Clinical features + GADA + T1D GRS	23.20924904 + (2.63093 * GADA†) + (-4.303557843 * Log(Age)) + (-5.360423718 *Log(BMI)) + (31.22606 * T1D GRS)

Supplementary Table 23: *To convert to probability use exp(lp)/(1+exp(lp)). †Dummy variable: negative = 0, positive = 1 ‡Dummy variables: false = 0, true = 1, AntiStatus1 = GADA positive only, AntiStatus2 = IA-2 positive only, AntiStatus3 = Both GADA and IA-2 positive.

For peer review only

TRIPOD Checklist: Prediction Model Development and Validation

Section/Topic	Item		Checklist Item	Page
**Title and abstract**				
Title	1	D;V	Identify the study as developing and/or validating a multivariable prediction model, the target population, and the outcome to be predicted.	1
Abstract	2	D;V	Provide a summary of objectives, study design, setting, participants, sample size, predictors, outcome, statistical analysis, results, and conclusions.	4,5
**Introduction**				
Background and objectives	3a	D;V	Explain the medical context (including whether diagnostic or prognostic) and rationale for developing or validating the multivariable prediction model, including references to existing models.	7
	3b	D;V	Specify the objectives, including whether the study describes the development or validation of the model or both.	9
**Methods**				
Source of data	4a	D;V	Describe the study design or source of data (e.g., randomized trial, cohort, or registry data), separately for the development and validation data sets, if applicable.	10 ,
	4b	D;V	Specify the key study dates, including start of accrual; end of accrual; and, if applicable, end of follow-up.	S.T.1
Participants	5a	D;V	Specify key elements of the study setting (e.g., primary care, secondary care, general population) including number and location of centres.	S.T.1
	5b	D;V	Describe eligibility criteria for participants.	10, SF2
	5c	D;V	Give details of treatments received, if relevant.	NA
Outcome	6a	D;V	Clearly define the outcome that is predicted by the prediction model, including how and when assessed.	11
	6b	D;V	Report any actions to blind assessment of the outcome to be predicted.	NA
Predictors	7a	D;V	Clearly define all predictors used in developing or validating the multivariable prediction model, including how and when they were measured.	11-13
	7b	D;V	Report any actions to blind assessment of predictors for the outcome and other predictors.	NA
Sample size	8	D;V	Explain how the study size was arrived at.	14, S.F.2
Missing data	9	D;V	Describe how missing data were handled (e.g., complete-case analysis, single imputation, multiple imputation) with details of any imputation method.	13,14
Statistical analysis methods	10a	D	Describe how predictors were handled in the analyses.	13,14
	10b	D	Specify type of model, all model-building procedures (including any predictor selection), and method for internal validation.	13,14
	10c	V	For validation, describe how the predictions were calculated.	13,14
	10d	D;V	Specify all measures used to assess model performance and, if relevant, to compare multiple models.	13,14
	10e	V	Describe any model updating (e.g., recalibration) arising from the validation, if done.	NA
Risk groups	11	D;V	Provide details on how risk groups were created, if done.	NA
Development vs. validation	12	V	For validation, identify any differences from the development data in setting, eligibility criteria, outcome, and predictors.	18
**Results**				
Participants	13a	D;V	Describe the flow of participants through the study, including the number of participants with and without the outcome and, if applicable, a summary of the follow-up time. A diagram may be helpful.	S.F.2
	13b	D;V	Describe the characteristics of the participants (basic demographics, clinical features, available predictors), including the number of participants with missing data for predictors and outcome.	S.F.2 S.T.3
	13c	V	For validation, show a comparison with the development data of the distribution of important variables (demographics, predictors and outcome).	S.T.9
Model development	14a	D	Specify the number of participants and outcome events in each analysis.	S.F.2
	14b	D	If done, report the unadjusted association between each candidate predictor and outcome.	17
Model specification	15a	D	Present the full prediction model to allow predictions for individuals (i.e., all regression coefficients, and model intercept or baseline survival at a given time point).	S.T.15 - 22
	15b	D	Explain how to the use the prediction model.	S.T. 23
Model performance	16	D;V	Report performance measures (with CIs) for the prediction model.	S.T.5
Model-updating	17	V	If done, report the results from any model updating (i.e., model specification, model performance).	NA
**Discussion**				
Limitations	18	D;V	Discuss any limitations of the study (such as nonrepresentative sample, few events per predictor, missing data).	21
Interpretation	19a	V	For validation, discuss the results with reference to performance in the development data, and any other validation data.	20
	19b	D;V	Give an overall interpretation of the results, considering objectives, limitations, results from similar studies, and other relevant evidence.	20-22
Implications	20	D;V	Discuss the potential clinical use of the model and implications for future research.	22
**Other information**				
Supplementary information	21	D;V	Provide information about the availability of supplementary resources, such as study protocol, Web calculator, and data sets.	19
Funding	22	D;V	Give the source of funding and the role of the funders for the present study.	23, 24

For peer review only

TRIPOD Checklist: Prediction Model Development and Validation

*Items relevant only to the development of a prediction model are denoted by D, items relating solely to a validation of a prediction model are denoted by V, and items relating to both are denoted D;V. We recommend using the TRIPOD Checklist in conjunction with the TRIPOD Explanation and Elaboration document.

Abbreviations:

NA = not applicable

S.T = Supplementary table

S.F = Supplementary Figure

For peer review only

Development and validation of multivariable clinical diagnostic models to identify type 1 diabetes requiring rapid insulin therapy in adults aged 18 to 50

Journal:	*BMJ Open*
Manuscript ID	bmjopen-2019-031586.R1
Article Type:	Original research
Date Submitted by the Author:	09-Jul-2019
Complete List of Authors:	Lynam, Anita; University of Exeter Medical School, The Institute of Biomedical & Clinical Science<br>McDonald, Timothy; Royal Devon and Exeter NHS Foundation Trust; University of Exeter Medical School, The Institute of Biomedical & Clinical Science<br>Hill, Anita; University of Exeter Medical School, The Institute of Biomedical & Clinical Science<br>Dennis, John; University of Exeter Medical School, The Institute of Biomedical & Clinical Science<br>Oram, Richard; University of Exeter, The Institute of Biomedical & Clinical Science; Royal Devon and Exeter NHS Foundation Trust<br>Pearson, Ewan; University of Dundee<br>Weedon, Michael; University of Exeter<br>Hattersley, Andrew; University of Exeter Medical School, Institute of Biomedical Science<br>Owen, Katharine; Oxford Centre for Diabetes Endocrinology and Metabolism<br>Shields, Beverley; University of Exeter,<br>Jones, Angus; University of Exeter Medical School, The Institute of Biomedical & Clinical Science; University of Exeter
**Primary Subject Heading**:	Diabetes and endocrinology
Secondary Subject Heading:	General practice / Family practice, Diagnostics
Keywords:	type 1 diabetes, type 2 diabetes, c-peptide, islet autoantibodies, prediction model, genetic risk score

SCHOLARONE™ Manuscripts

Title

Development and validation of multivariable clinical diagnostic models to identify type 1 diabetes requiring rapid insulin therapy in adults aged 18 to 50

Authors' names

Anita L Lynam, Timothy J McDonald, Anita V Hill, John M Dennis, Richard A Oram, Ewan R Pearson, Michael N Weedon, Andrew T Hattersley, Katharine R Owen, Beverley M Shields*, Angus G Jones*

* These authors contributed equally

Address for each author

1. Anita L Lynam, PhD student, Institute of Biomedical and Clinical Science, College of Medicine and Health, University of Exeter, Exeter, EX2 5DW, UK
2. Timothy J McDonald, Honorary Clinical Associate Professor, Academic Department of Clinical Biochemistry, Royal Devon and Exeter NHS Foundation Trust and Institute of Biomedical and Clinical Science, College of Medicine and Health, University of Exeter, Exeter, EX2 5DW, UK,
3. Anita V Hill, Research Project Manager, NIHR Exeter Clinical Research Facility, University of Exeter College of Medicine & Health, Exeter, EX2 5DW,
4. John M Dennis, Research Fellow in Medical Statistics, Institute of Biomedical and Clinical Science, College of Medicine and Health, University of Exeter, Exeter, EX2 5DW, UK,
5. Richard A Oram, Diabetes UK Harry Keen Fellow, Institute of Biomedical and Clinical Science, College of Medicine and Health, University of Exeter, Exeter, EX2 5DW, UK
6. Ewan R Pearson, Professor of Diabetic Medicine, Division of Molecular and Clinical Medicine, School of Medicine, University of Dundee, Ninewells Hospital and Medical School, Dundee, U.K
7. Michael N Weedon, Associate Professor, Institute of Biomedical and Clinical Science, College of Medicine and Health, University of Exeter, Exeter, EX2 5DW, UK,
8. Andrew T Hattersley, Professor of Molecular Medicine, Institute of Biomedical and Clinical Science, College of Medicine and Health, University of Exeter, Exeter, EX2 5DW, UK,
9. Katharine R Owen, Associate Professor of Diabetes, Oxford Centre for Diabetes Endocrinology and Metabolism, University of Oxford, Churchill Hospital, Oxford, OX3 7LE, UK and Oxford NIHR Biomedical Research Centre, Oxford University Hospitals Foundation Trust, John Radcliffe Hospital, Oxford, UK,

For peer review only - http://bmjopen.bmj.com/site/about/guidelines.xhtml

Corresponding authors

10. Beverley M Shields, Senior Lecturer in Medical Statistics, Institute of Biomedical and Clinical Science, College of Medicine and Health, University of Exeter, Exeter, EX2 5DW, UK

    Email: B.Shields@exeter.ac.uk

    Phone: +44 1392 408203

11. Angus G Jones, NIHR Clinician Scientist, Institute of Biomedical and Clinical Science, College of Medicine and Health, University of Exeter, Exeter, EX2 5DW

    angus.jones@exeter.ac.uk

    Phone: +44 1392 408538

For peer review only

Word count: abstract 299 main article 4014

Tables: 1

Figures: 3

Key Words

Type 1 diabetes

Type 2 diabetes

Classification

C-peptide

GADA

IA-2A

Type 1 Diabetes Genetic Risk Score

Abbreviations

GADA: Glutamic acid decarboxylase autoantibody

IA-2A: Islet antigen-2 autoantibody

ROC AUC: Area under the receiver operating characteristic curve

T1D GRS: Type 1 Diabetes Genetic Risk Score

YDX: Young Diabetes in Oxford Study

For peer review only - http://bmjopen.bmj.com/site/about/guidelines.xhtml

Abstract

Objective:

To develop and validate multivariable clinical diagnostic models to assist distinguishing between type 1 and type 2 diabetes in adults aged 18 to 50.

Design:

Multivariable logistic regression analysis was used to develop classification models integrating five pre-specified predictor variables, including clinical features (age of diagnosis, BMI) and clinical biomarkers (GADA and Islet Antigen 2 islet autoantibodies, Type 1 Diabetes Genetic Risk Score), to identify type 1 diabetes with rapid insulin requirement using data from existing cohorts.

Setting:

United Kingdom cohorts recruited from primary and secondary care.

Participants:

1,352 (model development) and 582 (external validation) participants diagnosed with diabetes between the age of 18 and 50 years of white European origin.

Main outcome measures:

Type 1 diabetes was defined by rapid insulin requirement (within 3 years of diagnosis) and severe endogenous insulin deficiency (C-peptide <200pmol/L). Type 2 diabetes was defined by either a lack of rapid insulin requirement or, where insulin treated within 3 years, retained endogenous insulin secretion (C-peptide >600pmol/L at ≥5 years diabetes duration). Model performance was assessed using area under the receiver operating characteristic curve (ROC AUC), and internal and external validation.

For peer review only - http://bmjopen.bmj.com/site/about/guidelines.xhtml

For peer review only

Results:

Type 1 diabetes was present in 13% of participants in the development cohort. All five predictor variables were discriminative and independent predictors of type 1 diabetes ($p<0.001$ for all) with individual ROC AUC ranging from 0.82 to 0.85. Model performance was high: ROC AUC range 0.90 [95%CI 0.88, 0.93] (clinical features only) to 0.97 [0.96, 0.98] (all predictors) with low prediction error. Results were consistent in external validation (clinical features and GADA ROC AUC 0.93 [0.90, 0.96]).

Conclusions:

Clinical diagnostic models integrating clinical features with biomarkers have high accuracy for identifying type 1 diabetes with rapid insulin requirement, and could assist clinicians and researchers in accurately identifying patients with type 1 diabetes.

For peer review only

Strengths and Limitations of this study

- Diabetes type is robustly defined using direct measurement of endogenous insulin secretion, an outcome closely related to treatment, education and monitoring requirements.
- A combination of a large development dataset and small number of predictors minimises risk of model overfitting, a common problem with diagnostic models of this nature.
- Models are robustly internally and externally validated
- The cross section nature of the development and validation cohorts means that time to insulin was self-reported and measurement of model predictors was not undertaken at diagnosis: both BMI and islet autoantibody prevalence may change over time.
- Models have been developed in white European populations with young adult onset diabetes: further work is required to extend this work to other age groups and ethnicities.

For peer review only

Introduction

Making the correct diagnosis of type 1 and type 2 diabetes is crucial for appropriate management, with guidelines for these conditions recommending very different glucose-lowering treatment and education [1-3]. These differences are predominantly driven by the rapid development of severe endogenous insulin deficiency in type 1 diabetes [1]. This means that patients with type 1 diabetes need rapid insulin treatment and are at risk of life-threatening ketoacidosis without insulin treatment. They develop a requirement for physiological insulin replacement (e.g. multiple injections, carbohydrate counting, pumps) due to the very high glycaemic variability associated with severe insulin deficiency [4, 5] and have poor glycaemic response to most adjuvant glucose-lowering therapies [6]. In contrast, patients with type 2 diabetes continue to make substantial endogenous insulin even many decades after diagnosis [7]. Glycaemia is therefore usually managed initially with lifestyle change or oral agents [4, 8] and, if insulin treatment is needed, a combination of simple insulin regimens and adjuvant non-insulin therapies [4, 5, 8, 9].

For peer review only

Correctly distinguishing between diabetes subtypes at diagnosis is often difficult and misclassification therefore common [10-12]. Current guidelines focus on etiopathological definitions without giving clear criteria for clinical use [1, 13]. In clinical practice, clinical features are predominantly used to determine diabetes subtype but only age at diagnosis and BMI have evidence for utility at diabetes onset, whereas other features used by clinicians such as symptoms at diagnosis, weight loss or ketosis do not have an evidence base [14]. Increasing obesity rates mean that many patients with type 1 diabetes will be obese and type 2 diabetes is occurring in the young [15]. Type 1 diabetes has been recently shown to occur at similar rates in those aged above and below 30 [16]. Therefore simple cut-offs based on age at diagnosis and BMI are unlikely to accurately diagnose diabetes type for many patients [1, 10]. Similarly, there is no single diagnostic test that can be used to classify diabetes robustly at diagnosis. While measurement of islet autoantibodies can assist classification, many patients with type 1 diabetes are islet autoantibody negative and many patients with the clinical phenotype of type 2 diabetes, without rapid insulin requirement, are islet autoantibody-positive [17]. A type 1 genetic risk score has been recently shown to assist diagnosis of diabetes type but this provides imperfect discrimination in isolation [18] .

For peer review only

In order to classify diabetes a suitable “gold standard” is necessary. As the key factor driving differences in treatment decisions between the two subtypes is the lack of endogenous insulin secretion, direct measurement of endogenous insulin secretion in longstanding insulin-treated diabetes (>3-5 years), using C-peptide, provides a robust classification that closely relates to treatment requirements [19]; patients with severe endogenous insulin deficiency (low C-peptide) have the high glucose variability, absolute insulin requirement, and lack of response to non-insulin glucose-lowering therapies that are characteristic of type 1 diabetes, regardless of their clinical characteristics and clinician’s diagnosis [7, 11, 19-23]. However, this test may have limited utility at diagnosis, as patients with recent onset type 1 diabetes may have retained endogenous insulin secretion [21, 24].

Clinical prediction models offer a way of combining multiple patient features and biomarkers to improve accuracy of diagnosis or prognosis. In diabetes, diagnostic models combining clinical features are available to predict the risk of prevalent or incident type 2 diabetes [25] and there is a model to identify monogenic forms of diabetes in patients with young-onset diabetes [26]. However there are no statistical prediction models to help distinguish type 1 and type 2 diabetes at diagnosis. We therefore aimed to develop and validate multivariable clinical diagnostic models that combine clinical features and biomarkers to identify type 1 diabetes (defined by rapid insulin requirement and severe endogenous insulin deficiency) in patients aged between 18 and 50 years at diabetes diagnosis.

For peer review only - http://bmjopen.bmj.com/site/about/guidelines.xhtml

Methods

We used logistic regression to model the relationship between each of clinical features and biomarkers, and type 1 diabetes defined by rapid insulin requirement and severe endogenous insulin deficiency (see below). We assessed the performance of the models using both internal validation and external validation.

Study population – development cohort

For model development, participants were identified from Exeter, UK-based cohorts [27-30]. These cohorts were participants with clinically diagnosed diabetes recruited from primary and secondary care. Summaries of the cohorts including recruitment and data collection methods are shown in Supplementary Table 1.

Participants were eligible for the study (model development or validation) if they had a clinical diagnosis of diabetes between the ages of 18 and 50 years. Participants with known secondary or monogenic diabetes [31], or a known disorder of the exocrine pancreas [32], were excluded. All participants included in this study were of white European origin.

Study population - external validation cohort

Participants meeting the study inclusion criteria were identified in the Young Diabetes in Oxford (YDX) study [33]. YDX is a cross-sectional study of participants diagnosed with diabetes (of any type) up to the age of 45 years, recruited from primary and secondary care in the Thames Valley region, UK. Participants with known secondary, pancreatic or monogenic diabetes were excluded.

Ethical approval

All cohort studies used for this research received ethical approval from the UK National Research Ethics Service. All participants gave written informed consent.

For peer review only - http://bmjopen.bmj.com/site/about/guidelines.xhtml

Model outcome: type 1 and type 2 diabetes definition

Type of diabetes was defined by the presence or absence of rapid insulin requirement and severe endogenous insulin deficiency after a diagnosis of diabetes, as follows:

Type 1 diabetes: Insulin treatment within <= 3 years of diabetes diagnosis and severe insulin deficiency (non–fasting C-peptide < 200pmol/L) [21].

Type 2 diabetes: Either 1) no insulin requirement for 3 years from diabetes diagnosis or 2) where insulin was started within 3 years of diagnosis, substantial retained endogenous insulin secretion (C-peptide >600pmol/L) at >=5 years diabetes duration.

Cohort participants not meeting the above criteria or with insufficient information were excluded from analysis, as type of diabetes and rapid insulin requirement could not be robustly defined.

Model predictors

Five pre-specified predictor variables were assessed, based on prior evidence and availability: age at diagnosis [14], BMI [14], GADA and IA-2A islet autoantibodies [17, 34], and a Type 1 diabetes Genetic Risk Score (T1D GRS) [18].

Assessment of clinical features

At study recruitment visit, clinical history including time to insulin and age at diagnosis were self-reported by participants in an interview with a research nurse. Height and weight were measured for calculation of BMI.

Laboratory Measurement

C-peptide

In the development cohort, C-peptide was measured on stored EDTA taken at study

For peer review only - http://bmjopen.bmj.com/site/about/guidelines.xhtml

visits (non-fasting random [35], fasting, or at 90 minutes in a post-mixed-meal tolerance test (majority 87% non-fasting)). With specific additional consent, C-peptide was also measured on post-recruitment non-fasting EDTA samples collected as part of routine clinical care. Fasting C-peptide values were multiplied by 2.5 to non-fasting equivalent [21]. The median C-peptide value was used where more than one eligible C-peptide value was available (62% of participants requiring this measure for outcome definition). C-peptide was measured using an electrochemiluminescence immunoassay on a Roche Diagnostics E170 analyser (Roche, Mannheim, Germany) by the Academic Department of Blood Sciences at the Royal Devon and Exeter Hospital. In the external validation cohort, C-peptide measurement was performed in the Biochemistry Laboratory of the Oxford University Hospitals NHS Trust using a chemiluminescence immunoassay on an ADVIA Centaur analyser (Siemens Healthcare Diagnostics Ltd).

Islet autoantibodies

In the development cohort, GADA and IA-2A were measured on EDTA taken at recruitment or obtained from local laboratory records. Both islet autoantibodies were measured using the RSR Ltd ELISA assays (RSR Ltd, Cardiff, UK) on the Dynex DS2 ELISA Robot (Dynex Technologies, Worthing, UK) by the Academic Department of Blood Sciences at the Royal Devon and Exeter Hospital. The department participates in the International Autoantibody Standardization Programme. The cut-off for positivity for GADA was ≥11 units/ml and IA-2A was ≥15 units/ml, based on the 97.5th centile of 1,559 controls without diabetes [34].

In the external validation cohort, GADA was measured by a radioimmunoassay using ^{35}S-labeled full-length GAD65 by the Department of Clinical Science, University of Bristol, Bristol, U.K. Results were expressed in World Health Organization (WHO)

For peer review only - http://bmjopen.bmj.com/site/about/guidelines.xhtml

For peer review only

units per millilitre derived from a standard curve calibrated from international reference material (National Institute for Biological Standards and Control code 97/550). The cut-off for positivity for GADA was 13 WHO Units/mL initially, using a local assay (samples measured n=218, DASP2010 sensitivity 88% at 93% specificity) and changed to 33 DK Units/mL later in the study (standard assay, DASP2010 sensitivity 80%, specificity 97%).

Type 1 Diabetes Genetic Risk Score (T1D GRS)

The T1D GRS was calculated on the development cohort as previously described [18]. In brief, T1D GRS consists of 30 common type 1 diabetes genetic variants (single nucleotide polymorphisms (SNPs)) from HLA and non-HLA loci; each variant is weighted by its effect size on type 1 diabetes risk from previously published literature, with weights for DR3/DR4-DQ8 assigned based on imputed haplotypes (Supplementary Table 2). All SNPs had an INFO > 0.8. The combined score represents an individual's genetic susceptibility to type 1 diabetes. T1D GRS calculation was not performed if genotyping results were missing for either of the two alleles with the greatest weighting (DR3/DR4-DQ8 or HLA_DRB1_15) or if more than two of any other SNPs were missing. For ease of clinical interpretation the score is presented in this article as the score and centile position of the distribution in the Wellcome Trust Case Control Consortium type 1 diabetes population [36].

Statistical analysis

Model development

We used logistic regression analysis to develop the models. Models were developed on a complete-case basis.

For peer review only - http://bmjopen.bmj.com/site/about/guidelines.xhtml

Age at diagnosis, BMI and T1D GRS were modelled as continuous variables and transformations used to ensure linearity on the logit scale [37] (Supplementary Figures 1A and 1B). GADA and IA-2A were both dichotomized into negative or positive based on the cut-off for positivity in line with how the results are reported clinically [2]. Sample sizes were checked using both minimal Events Per Variable (EPV) criteria (>=10) [38] and square root of the mean squared prediction error (rMPSE) [39] and were considered sufficient for reliable diagnostic modelling.

Models were built and validated in four stages, this staged development sequence was selected in order of clinical availability of the predictors and, as some participants had missing diagnostic test data, to maximise the sample size at each stage: 1) model including only clinical features (age at diagnosis and BMI); 2) Addition of GADA to the linear predictor from model 1; 3) Addition of both GADA and IA-2A to the linear predictor from model 1; 4) Addition of T1D GRS to model 3 linear predictor.

Evaluation of model performance: Internal validation

Three internal validation techniques were used to assess the discrimination and calibration performance of the models: 1) directly using the data used to develop the model (apparent validation, ROC AUC); 2) Jack-knife cross-validation; 3) Bootstrapping (with replacement method) [37]

Evaluation of model performance: External validation

Performances of model 1 (clinical features) and model 2 (clinical features + GADA), were evaluated in the YDX study cohort. We were unable to externally evaluate models 3 and 4 as IA-2A autoantibodies and T1D GRS were not available in the YDX study.

For peer review only - http://bmjopen.bmj.com/site/about/guidelines.xhtml

Model comparisons

Four nested replica models were built on the subset of participants with complete data on all predictor variables (n = 943). The predictive information of each additional predictor on the model performance was assessed using the Unitless Index of Adequacy [37], log likelihood ratio test [37], Net Reclassification Improvement and Integrated Discrimination Improvement [40] .

Sensitivity analysis

Model development of all 4 models was repeated on 943 participants with complete data. To assess performance of biomarker models in those difficult to classify on clinical features alone model AUC ROC was repeated for each model in participants with intermediate age of diagnosis (range 25-35 years (inclusive)) and BMI (range 25-35 kg/m^2 (inclusive)).

All statistical analyses were performed using STATA version 15, STATA Corp, Texas, USA (unless otherwise stated).

Patient Involvement

Patients with diabetes were involved in prioritising the research question and development of the original funding application. This study did not involve the collection of primary data, but this research was reviewed and access to data approved by the Peninsula Research Bank Lay steering committee, who also contributed to the design and development of the source cohort studies.

For peer review only - http://bmjopen.bmj.com/site/about/guidelines.xhtml

Results

1,352 (type 1 diabetes n = 179) participants met analysis inclusion criteria for the clinical features model with 943 participants having all predictor variables measured. A flow diagram describing the flow of participants through the study is shown in Supplementary Figure 2. Only 37 (2.7% of the cohort) had an undefinable outcome due to intermediate C-peptide levels (200-600pmol/L when insulin-treated within 3 years of diagnosis). The remaining exclusions were due to either missing data or short duration of diabetes. The characteristics and type 1 diabetes outcome prevalence of the included participants were similar in all four development samples (Supplementary Table 3). There were no clinically relevant differences in the characteristics of the participants who were excluded from the fourth model development stage (n = 409) (Supplementary Table 4). Islet autoantibodies and C-peptide were measured at median 13 years and 16 years post-diagnosis respectively.

Clinical features or biomarkers in isolation overlap substantially between diabetes types (Figure 1)

Participants with type 1 diabetes and rapid insulin requirement were diagnosed younger compared to the participants with type 2 diabetes (median 27 vs 44 years, $p < 0.001$) and had a lower BMI (median 26 vs 34 kg/m^2, $p < 0.001$). Positive autoantibodies (GADA, IA-2A or both) were more common in the participants with type 1 diabetes (71% of participants with type 1 diabetes vs 5% of participants with type 2 diabetes, $p < 0.001$). Patients with type 1 diabetes had a higher T1D GRS (median 0.27 vs 0.23 (equivalent to 40th and 4th centile of the Wellcome Trust Case Control Consortium population with type 1 diabetes [36], $p < 0.001$). These features

For peer review only - http://bmjopen.bmj.com/site/about/guidelines.xhtml

overlapped substantially between participants meeting criteria for type 1 and type 2 diabetes (Figure 1 (A – D)) with AUC ROC for these features in isolation: 0.82 (age at diagnosis), 0.83 (BMI), 0.83 (islet autoantibodies) and 0.85 (T1D GRS).

Combining clinical features using a diagnostic model improves model discrimination

In model 1, age at diagnosis and BMI were both significant independent predictors of type 1 diabetes, with the odds of having type 1 diabetes increasing with younger age at diagnosis and lower BMI. Combined, these features provided excellent discrimination (ROC AUC=0.904, perfect test = 1) (Figure 2a), with low probabilities capturing the majority of participants with type 2 diabetes and type 1 diabetes being very unlikely (Figure 2b; sensitivity, specificity, and positive and negative predictive values at various probability cut-offs are reported in Table 1). In successive models adding in GADA (model 2 (figures 2c and 2d)), then IA-2A (model 3 (figures 2e and 2f)) and then T1D GRS (model 4 (figures 2g and 2h)), the addition of each predictor to the previous model resulted in significant improvements in discrimination (Supplementary Table 5) and model fit (Supplementary Tables 6 and 7). In sensitivity analysis, results were similar when restricting all models to only the 943 participants with complete data on all predictor variables (Supplementary Table 8).

In further sensitivity analysis restricting analysis to those most difficult to classify on clinical features alone due to both intermediate BMI (range 25-35 kg/m^2 (inclusive)) and age of diagnosis (range 25-35 years (inclusive)), model performance remained high for models incorporating biomarker measurement (clinical features + islet autoantibodies AUC ROC 0.89, clinical features + islet autoantibodies + T1D GRS AUC ROC 0.95) Supplementary Table 9. This compares to AUC ROC of 0.72 for

For peer review only - http://bmjopen.bmj.com/site/about/guidelines.xhtml

GADA and IA-2A measurement alone, and 0.89 for T1D GRS measurement alone in this sub population (n = 71).

Internal validation suggests robust model performance

Results of the internal validation bootstrap (Supplementary Table 5) indicate good model discrimination, with very similar model performance in bootstrapped samples (near identical ROC AUC for all models (max decrease = 0.0018)), high calibration indicating the predicted probabilities closely fit the observed probabilities (calibration slope range 0.98 - 1.00 (0.9 – 1.1 is indicative of good calibration)), and very low levels of optimism suggesting little error due to overfitting.

Model performance remains high in an external validation cohort with different characteristics

582 participants in the YDX study met criteria for external validation (Supplementary Figure 3). Compared to the participants in the Exeter model development cohort, the participants in the YDX study were younger at diagnosis (consistent with the narrower age range in YDX (18-45y) (median 37 years vs 43 years, $p < 0.001$)), had a lower BMI (median 31 kg/m^2 vs 33 kg/m^2, $p < 0.001$), had a higher percentage of GADA (20% versus 12%, $p < 0.001$) and a higher prevalence of type 1 diabetes by study definition (22% vs 14%, $p < 0.001$) (see Supplementary Table 10 for participant characteristics).

There was a small decrease in performance of the model 1 (clinical features) and model 2 (clinical features and GADA) when they were applied to the external validation samples but both still showed high levels of discrimination despite differences in the two cohorts (ROC AUC = 0.865 and 0.930 for models 1 (Figures 3a, 3b and 3c) and 2 (Figures 3d, 3e and 3f), respectively, (Supplementary Table

For peer review only - http://bmjopen.bmj.com/site/about/guidelines.xhtml

11). Both models slightly over estimated type 1 diabetes prevalence but there was no evidence of miscalibration (Figures 3b and 3e, Supplementary Table 11). Sensitivity and specificity in the validation cohort are shown in Supplementary Table 12.

Participants with high model probability type 1 diabetes but type 2 diabetes outcome have the characteristics of type 1 diabetes but took > 3 years to commence insulin therapy.

Supplementary Table 13 shows the characteristics of 12 participants in the external validation cohort with >80% model type 1 diabetes probability, but an actual model outcome of type 2 diabetes. These participants had the clinical characteristics associated with type 1 diabetes with GADA positivity and low C-peptide in the majority of cases (median C-peptide 120 pmol/L). However the time to insulin was > 3 years in GADA positive cases, suggesting slow onset autoimmune diabetes. In contrast, the 6 participants who had a low model type 1 diabetes probability (< 16%) but an actual model outcome of type 1 diabetes (Supplementary Table 14) had features associated with type 2 diabetes.

Online calculator

The four models have been incorporated into an online calculator (beta version available at https://www.diabetesgenes.org/t1dt2d-prediction-model/). An additional four models with different combinations of the five predictor variables were also developed for the online calculator, to allow every combination of clinical features plus the other biomarkers as optional. As expected, ROC AUC and prediction error results for these four additional models were intermediate between the basic clinical features model and the full model with all features (see Supplementary Table 15).

For peer review only

Supplementary Tables 16 - 23 inclusive show the β coefficients and odds ratios for all models. The regression equations for the online calculator are shown in Supplementary Table 24.

Discussion

We have developed, evaluated and validated clinical diagnostic models combining age at diagnosis, BMI, GADA, IA-2, and T1D GRS to provide estimates of a patient's risk of having type 1 diabetes requiring rapid insulin therapy from diagnosis. These models show high performance, and could potentially assist classification of diabetes in clinical practice and provide a tool for evidence based classification in research cohorts.

Model performance was optimised in the model combining all five predictors (ROC AUC 0.97). However, all models performed well with ROC AUC > 0.9 and low cross-validated prediction errors in development. The results of the external validation provide additional confidence in model performance. This was undertaken in a distinct dataset with different type 1 diabetes prevalence and biochemical assays.

This is the first study developing clinical diagnostic models for classification of type 1 and 2 diabetes. Key strengths of this study include our systematic approach to model development including robust internal and external validation [41]. Our staged approach to model development means that we have maximised the information gained from each predictor. Our model is parsimonious, we have used only five predictors previously shown to be associated with type 1 diabetes. This, in combination with large datasets, mean we have a high number of events per variable and very low risk of overfitting, a common problem with diagnostic models of this nature. Our use of predominantly population-based cohorts recruited largely from a

For peer review only - http://bmjopen.bmj.com/site/about/guidelines.xhtml

primary care setting (for model development) means our results are likely to reflect true associations in patients seen in clinical practice. The overall prevalence of study defined type 1 diabetes of 13% in our development dataset is close to the 11% reported type 1 diabetes prevalence at diagnosis in a UK population aged 20-50 [42].

A limitation of our study is the cross-sectional nature of our cohorts meaning that age at diagnosis and time to insulin were self-reported at a single visit. Insulin commencement was also based on clinical decision-making rather than a trial protocol. BMI and antibodies were measured at median 13 years after diagnosis. BMI, and GAD and IA-2A antibodies change modestly over time in adult onset diabetes, with previous research suggesting an approximately 18% lower combined GADA and IA-2A prevalence after 13.5 years diabetes duration in this age group [43], and BMI having higher discrimination for diabetes classification when measured at diagnosis [44]. The potential impact on the results of BMI and islet autoantibodies having been measured some years post diagnosis is that the predictions may be under-estimated. The lack of information at diagnosis also meant we were unable to assess whether other features available at diagnosis may assist classification, such as presentation glycaemia, ketosis, or weight loss. A prospective study to validate these models, and assess whether other features may assist classification is therefore ongoing (https://clinicaltrials.gov/ct2/show/NCT03737799).

A further limitation is that this model has been developed and tested in a white European population with young onset diabetes, extension of this work to non-white populations and older age groups is therefore a priority for future research.

These models have the potential to help robustly classify diabetes in research cohorts, and may have particular utility where genetic but not antibody data is

For peer review only - http://bmjopen.bmj.com/site/about/guidelines.xhtml

available, a common situation in many biobanks. They may also assist clinical decision making, with the important caveats that this evidence can only be applied to patients aged 18-50, of white ethnicity, and that these models are intended to act as a decision aid in conjunction with other information which a clinician may use to inform treatment decisions (for example severity of hyperglycaemia): they do not replace expert clinical opinion. A web-based calculator and smartphone app could be used to display the estimate of the patient's probability of having type 1 diabetes based on the predictor variable values entered. The models can be used with age of diagnosis and BMI as a minimum; users will then have a choice to add results of GADA, IA-2A and T1D GRS in any combination. This could therefore be used by clinicians as a triage-based approach to diabetes subtype diagnosis. For example, probabilities calculated on clinical features could be used as the basis for antibody testing, or the additional value likely to be gained from antibody or genetic testing could be assessed by inputting dummy results into the model. We propose providing the continuous probability outcome of the models rather than giving a threshold. This is because the decision made on whether to commence insulin for a given probability of type 1 diabetes will vary enormously due to other factors. For example temporary insulin treatment may be appropriate regardless of likely classification where hyperglycaemia is severe, and in some circumstances it may be appropriate to trial oral therapy even where type 1 diabetes has a high probability, for example where a person's occupation would be affected by insulin treatment and they can be carefully monitored for glycaemic deterioration.

For peer review only

In conclusion clinical diagnostic models integrating clinical features with biomarkers have high accuracy for identifying type 1 diabetes with rapid insulin requirement in white participants aged 18 to 50 at diabetes diagnosis, and may assist clinicians in identifying patients with type 1 diabetes in clinical practice.

Acknowledgments

The authors thank participants who took part in these studies and the research teams who undertook cohort recruitment. We thank Catherine Angwin of the NIHR Exeter Clinical Research Facility for assistance with data preparation, and Rachel Nice of the Blood Sciences Department, Royal Devon and Exeter Hospital for assistance with sample analysis. We are grateful to Maarten van Smeden for allowing us to access to his Beyond EPV R-Shiny app (BETA version).

Transparency statement

The manuscript's guarantors (AGJ and BMS) affirms that the manuscript is an honest, accurate, and transparent account of the study being reported; that no important aspects of the study have been omitted; and that any discrepancies from the study as planned have been explained.

Author Contributions

A.L.L, B.M.S and A.G.J conceived the idea and designed the study. A.L.L, T.J.M, A.V.H, E.R.P, M.N.W, A.T.H, K.R.O and A.G.J researched the data. A.L.L analysed the data with assistance from B.M.S and A.G.J. T.J.M, J.M.D, R.A.O, A.T.H and K.R.O discussed and contributed to study design and provided support for the analysis and interpretation of results. A.L.L drafted the manuscript with assistance from B.M.S and A.G.J. All authors critically revised the manuscript and approved the

For peer review only

final version. The corresponding author attests that all listed authors meet authorship criteria and that no others meeting the criteria have been omitted.

A.G.J. and BMS are the guarantors of this work.

Funding:

This work was funded by NIHR Clinician Scientist award CS-2015-15-018. The Young Diabetes in Oxford Study was funded by the European Community FP7 programme CEED3 (HEALTH-F2-2008-223211), the Oxford Hospitals Charitable Fund and by the NIHR Oxford Biomedical Research Centre. The Diabetes Alliance for Research in England (DARE) study was funded by the Wellcome Trust and supported by the Exeter NIHR Clinical Research Facility. The MASTERMIND study was funded by the UK Medical Research Council (MR/N00633X/) and supported by the NIHR Exeter Clinical Research Facility. The PRIBA study was funded by the National Institute for Health Research (UK) (DRF-2010-03-72) and supported by the NIHR Exeter Clinical Research Facility.

T.J.M is a National Institute for Health Research Senior Clinical Senior Lecturer. R.A.O is supported by a Diabetes UK Harry Keen Fellowship (16/0005529). . E.R.P. is a Wellcome Trust New Investigator (102820/Z/13/Z). A.T.H and B.M.S are supported by the NIHR Exeter Clinical Research Facility. A.T.H is a Wellcome Trust Senior Investigator and NIHR Senior Investigator. A.G.J is supported by an NIHR Clinician Scientist award (CS-2015-15-018).

The views expressed in this article are those of the authors do not necessarily represent those of the National Institute for Health Research, the National Health Service or the Department of Health.

For peer review only - http://bmjopen.bmj.com/site/about/guidelines.xhtml

The study sponsor was not involved in the design of the study; the collection, analysis, and interpretation of data; writing the report; or the decision to submit the report for publication

Copyright/license for publication:

The Corresponding Author has the right to grant on behalf of all authors and does grant on behalf of all authors, a worldwide licence to the Publishers and its licensees in perpetuity, in all forms, formats and media (whether known now or created in the future), to i) publish, reproduce, distribute, display and store the Contribution, ii) translate the Contribution into other languages, create adaptations, reprints, include within collections and create summaries, extracts and/or, abstracts of the Contribution, iii) create any other derivative work(s) based on the Contribution, iv) to exploit all subsidiary rights in the Contribution, v) the inclusion of electronic links from the Contribution to third party material where-ever it may be located; and, vi) licence any third party to do any or all of the above.

Competing interests declaration:

All authors have completed the ICMJE uniform disclosure form at www.icmje.org/coi_disclosure.pdf and declare: no support from any organisation for the submitted work; no financial relationships with any organisations that might have an interest in the submitted work in the previous three years; no other relationships or activities that could appear to have influenced the submitted work.

Ethics approval:

Not required

For peer review only - http://bmjopen.bmj.com/site/about/guidelines.xhtml

Data Availability

Data from the Exeter cohorts included in this research is held by the Peninsula Research Bank, managed by the NIHR Exeter Clinical Research Facility. Guidance for applying to use the Peninsula Research Bank resource are given on the following website: https://exetercrfnihr.org/about/exeter-10000-prb/

For peer review only

References

[1] American Diabetes Association (2018) 2. Classification and Diagnosis of Diabetes: Standards of Medical Care in Diabetes—2018. Diabetes care 41: S13 - S27

[2] National Institute for Health and Care Excellence (2015) Type 1 diabetes in adults: diagnosis and management (NICE guideline NG17). Available from https://www.nice.org.uk/guidance/ng17, accessed 14/08/2018

[3] National Institute for Health and Care Excellence (2015) Type 2 diabetes in adults: management (NICE guideline NG28). Available from https://www.nice.org.uk/guidance/ng28, accessed 14/08/2018

[4] American Diabetes Association (2018) 8. Pharmacologic Approaches to Glycemic Treatment: Standards of Medical Care in Diabetes—2018. Diabetes care 41: S73 - S85

[5] DeWitt DE, Hirsch IB (2003) Outpatient insulin therapy in type 1 and type 2 diabetes mellitus: Scientific review. JAMA 289: 2254-2264

[6] Frandsen CS, Dejgaard TF, Madsbad S (2016) Non-insulin drugs to treat hyperglycaemia in type 1 diabetes mellitus. The Lancet Diabetes & Endocrinology 4: 766-780

[7] (1998) Effect of intensive therapy on residual β-cell function in patients with type 1 diabetes in the diabetes control and complications trial: A randomized, controlled trial. Annals of Internal Medicine 128: 517-523

[8] Inzucchi SE, Bergenstal RM, Buse JB, et al. (2012) Management of hyperglycaemia in type 2 diabetes: a patient-centered approach. Position statement of the American Diabetes Association (ADA) and the European Association for the Study of Diabetes (EASD). Diabetologia 55: 1577-1596

[9] Riddle MC (2008) Combined Therapy With Insulin Plus Oral Agents: Is There Any Advantage? Diabetes care 31: S125

[10] Farmer A, Fox R (2011) Diagnosis, classification, and treatment of diabetes. BMJ 342

[11] Hope SV, Knight BA, Shields BM, et al. (2018) Random non-fasting C-peptide testing can identify patients with insulin-treated type 2 diabetes at high risk of hypoglycaemia. Diabetologia 61: 66-74

[12] Stone MA, Camosso-Stefinovic J, Wilkinson J, De Lusignan S, Hattersley AT, Khunti K (2010) Incorrect and incomplete coding and classification of diabetes: a systematic review. Diabetic Medicine 27: 491-497

[13] World Health Organization (2006) Definition and diagnosis of diabetes mellitus and intermediate hyperglycaemia: report of a WHO/IDF consultation. In:

[14] Shields BM, Peters JL, Cooper C, et al. (2015) Can clinical features be used to differentiate type 1 from type 2 diabetes? A systematic review of the literature. BMJ open 5

[15] Rosenbloom AL, Joe JR, Young RS, Winter WE (1999) Emerging epidemic of type 2 diabetes in youth. Diabetes care 22: 345

[16] Thomas NJ, Jones SE, Weedon MN, Shields BM, Oram RA, Hattersley AT (2018) Frequency and phenotype of type 1 diabetes in the first six decades of life: a cross-sectional, genetically stratified survival analysis from UK Biobank. The Lancet Diabetes & Endocrinology 6: 122-129

[17] Niskanen LK, Tuomi T, Karjalainen J, Groop LC, Uusitupa MIJ (1995) GAD Antibodies in NIDDM: Ten-year follow-up from the diagnosis. Diabetes care 18: 1557

For peer review only - http://bmjopen.bmj.com/site/about/guidelines.xhtml

[18] Oram RA, Patel K, Hill A, et al. (2016) A Type 1 diabetes genetic risk score can aid discrimination between Type 1 and Type 2 diabetes in young adults. Diabetes care 39: 337-344
[19] Jones AG, Besser REJ, Shields BM, et al. (2012) Assessment of endogenous insulin secretion in insulin treated diabetes predicts postprandial glucose and treatment response to prandial insulin. BMC Endocrine Disorders 12: 6
[20] Steffes MW, Sibley S, Jackson M, Thomas W (2003) Beta-cell function and the development of diabetes-related complications in the diabetes control and complications trial. Diabetes care 26: 832-836
[21] Jones AG, Hattersley AT (2013) The clinical utility of C-peptide measurement in the care of patients with diabetes. Diabetic Medicine 30: 803-817
[22] Jones AG, McDonald TJ, Shields BM, et al. (2016) Markers of beta cell failure predict poor glycemic response to GLP-1 receptor agonist therapy in type 2 diabetes. Diabetes care 39: 250-257
[23] Chow LS, Chen H, Miller ME, Marcovina SM, Seaquist ER (2015) Biomarkers related to severe hypoglycaemia and lack of good glycaemic control in ACCORD. Diabetologia 58: 1160-1166
[24] Thunander M, Törn C, Petersson C, Ossiansson B, Fornander J, Landin-Olsson M (2012) Levels of C-peptide, body mass index and age, and their usefulness in classification of diabetes in relation to autoimmunity, in adults with newly diagnosed diabetes in Kronoberg, Sweden. European journal of endocrinology 166: 1021-1029
[25] Collins GS, Mallett S, Omar O, Yu L-M (2011) Developing risk prediction models for type 2 diabetes: a systematic review of methodology and reporting. BMC Medicine 9: 103-103
[26] Shields BM, McDonald TJ, Ellard S, Campbell MJ, Hyde C, Hattersley AT (2012) The development and validation of a clinical prediction model to determine the probability of MODY in patients with young-onset diabetes. Diabetologia 55: 1265-1272
[27] DiabetesGenes.org Diabetes Alliance for Research in England (DARE) Available from https://www.diabetesgenes.org/current-research/dare/, accessed 23 November 2017
[28] ClinicalTrials.gov RetroMASTER - Retrospective Cohort MRC ABPI STratification and Extreme Response Mechanism in Diabetes. Available from https://www.clinicaltrials.gov/ct2/show/NCT02109978
[29] ClinicalTrials.gov MASTERMIND - Understanding Individual Variation in Treatment Response in Type 2 Diabetes (Mastermind). Available from https://www.clinicaltrials.gov/ct2/show/NCT01847144?term=mastermind accessed 31 July 2018
[30] clinicaltrials.gov PROMASTER - PROspective Cohort MRC ABPI STratification and Extreme Response Mechanism in Diabetes (PROMASTER). Available from https://www.clinicaltrials.gov/ct2/show/NCT02105792?term=promaster&rank=1, accessed 31 July 2018
[31] Shields BM, Shepherd M, Hudson M, et al. (2017) Population-Based Assessment of a Biomarker-Based Screening Pathway to Aid Diagnosis of Monogenic Diabetes in Young-Onset Patients. Diabetes care 40: 1017-1025
[32] Woodmansey C, McGovern AP, McCullough KA, et al. (2017) Incidence, Demographics, and Clinical Characteristics of Diabetes of the Exocrine Pancreas (Type 3c): A Retrospective Cohort Study. Diabetes care

For peer review only - http://bmjopen.bmj.com/site/about/guidelines.xhtml

[33] Thanabalasingham G, Pal A, Selwood MP, et al. (2012) Systematic Assessment of Etiology in Adults With a Clinical Diagnosis of Young-Onset Type 2 Diabetes Is a Successful Strategy for Identifying Maturity-Onset Diabetes of the Young. Diabetes care 35: 1206-1212
[34] McDonald TJ, Colclough K, Brown R, et al. (2011) Islet autoantibodies can discriminate maturity-onset diabetes of the young (MODY) from Type 1 diabetes. Diabetic medicine : a journal of the British Diabetic Association 28: 1028-1033
[35] Hope SV, Knight BA, Shields BM, Hattersley AT, McDonald TJ, Jones AG (2016) Random non-fasting C-peptide: bringing robust assessment of endogenous insulin secretion to the clinic. Diabetic medicine : a journal of the British Diabetic Association 33: 1554-1558
[36] The Wellcome Trust Case Control C (2007) Genome-wide association study of 14,000 cases of seven common diseases and 3,000 shared controls. Nature 447: 661-678
[37] Harrell F (2015) Regression Modeling Strategies. Springer International Publishing, Switzerland
[38] Peduzzi P, Concato J, Kemper E, Holford TR, Feinstein AR (1996) A simulation study of the number of events per variable in logistic regression analysis. J Clin Epidemiol 49: 1373-1379
[39] van Smeden M, Moons KGM, de Groot JAH, et al. (2018) Sample size for binary logistic prediction models: Beyond events per variable criteria. Statistical Methods in Medical Research: 0962280218784726
[40] Steyerberg EW, Vickers AJ, Cook NR, et al. (2010) Assessing the performance of prediction models: a framework for some traditional and novel measures. Epidemiology (Cambridge, Mass) 21: 128-138
[41] Steyerberg EW, Vergouwe Y (2014) Towards better clinical prediction models: seven steps for development and an ABCD for validation. European Heart Journal 35: 1925-1931
[42] Scottish Diabetes Survey 2017 http://www.diabetesinscotland.org.uk/Publications/SDS%202017.pdf . Accessed 03/05/2019
[43] Tridgell DM, Spiekerman C, Wang RS, Greenbaum CJ (2011) Interaction of Onset and Duration of Diabetes on the Percent of GAD and IA-2 Antibody–Positive Subjects in the Type 1 Diabetes Genetics Consortium Database. Diabetes care 34: 988
[44] Hope SV, Wienand-Barnett S, Shepherd M, et al. (2016) Practical Classification Guidelines for Diabetes in patients treated with insulin: a cross-sectional study of the accuracy of diabetes diagnosis. The British journal of general practice : the journal of the Royal College of General Practitioners 66: e315-322

For peer review only

Figure Legends

Figure 1: Density plots for (A) age at diagnosis, (B) BMI and (D) T1D GRS. Stacked bar chart (C) showing percentages of participants (total n = 943 (stage 4 model development sample)) by actual type 1 diabetes outcome and GADA/IA-2A status. Dashed line shows the distribution for type 2 diabetes (T2D) (n = 815), solid line shows the distribution for type 1 diabetes (T1D) (n = 128) of participants included in the stage 4 model development.

Figure 2: Development sample validation results. Plots are the results from the validation of the models. First row (a and b): clinical features logistic regression model (n = 1,315). Second row (c and d): clinical features + GADA logistic regression model (n = 1,036). Third row (e and f): clinical features + GADA + IA-2A logistic regression model (n = 1,025). Fourth row (g and h): clinical features + GADA + IA-2A + T1D GRS logistic regression model (n =943). Plots (a), (c), (e), & (g) are ROC curves showing discrimination ability of the models. Plots (b), (d), (f) & (h) are boxplots of fitted model probabilities grouped by actual diabetes outcome.

Figure 3: External validation results. Plots on the first row (a, b, c) are the results from the external validation of the clinical features logistic regression model applied to participants in the YDX study (n = 582). The second row of plots (d, e, f) are the results from the external validation of the clinical features + GADA logistic regression model applied to participants in the YDX study (n = 549). Plots (a) & (d) are ROC curves showing discrimination ability of the models, dashed line represents the reference line. Plots (b) & (e) are calibration plots. Plots (c) & (f) are boxplots of fitted model probabilities grouped by actual diabetes outcome.

For peer review only - http://bmjopen.bmj.com/site/about/guidelines.xhtml

Tables

Clinical features (n = 1,352)						
	Probability (%) cut-off for classifying type 1 diabetes					
	10	30	50	70	90	12 (Youden's Index)
Sensitivity/specificity (%)	85/79	64/95	49/98	35/99	15/100	83/83
Accuracy (%)	80	90	91	90	89	83
Positive predictive value (PPV) (%)	38	64	79	83	90	42
Negative predictive value (NPV) (%)	97	95	93	91	89	97
Clinical features + GADA (n = 1,036)						
	Probability (%) cut-off for classifying type 1 diabetes					
	10	30	50	70	90	16 (Youden's Index)
Sensitivity/specificity (%)	90/88	80/96	66/97	52/99	31/100	86/92
Accuracy (%)	89	94	93	92	90	92
Positive predictive value (PPV) (%)	55	75	80	85	92	64
Negative predictive value (NPV) (%)	98	97	95	93	90	98
Clinical features + GADA + IA-2A (n = 1,025)						
	Probability (%) cut-off for classifying type 1 diabetes					
	10	30	50	70	90	12 (Youden's Index)
Sensitivity/specificity (%)	91/91	80/96	69/98	57/99	37/100	90/92
Accuracy (%)	91	94	94	93	92	92
Positive predictive value (PPV) (%)	59	75	81	85	92	62
Negative predictive value (NPV) (%)	99	97	96	94	92	98
Clinical features + GADA + IA-2A + T1D GRS (n = 943)						
	Probability (%) cut-off for classifying type 1 diabetes					
	10	30	50	70	90	14 (Youden's Index)
Sensitivity/specificity (%)	92/90	84/96	74/98	63/99	41/100	91/93
Accuracy (%)	90	95	94	94	92	93
Positive predictive value (PPV) (%)	59	78	83	88	93	67
Negative predictive value (NPV) (%)	99	98	96	94	92	99

Table 1: Model performance at different cut-offs for all four logistic regression models (development cohort). Positive and negative predictive values relate to type 1 diabetes.
Accuracy = (true positives + true negatives)/total number of participants.
Positive predictive value (PPV) = [(sensitivity × prevalence)/[(sensitivity × prevalence) + ([1 – specificity] × [1−prevalence])].
Negative predictive value (NPV) = [specificity × (1 − prevalence)]/[(specificity × [1 − prevalence]) + ([1 − sensitivity] × prevalence)].
Youden's Index - best trade-off between sensitivity and specificity (sensitivity+specificity – 1).

For peer review only

For peer review only - http://bmjopen.bmj.com/site/about/guidelines.xhtml

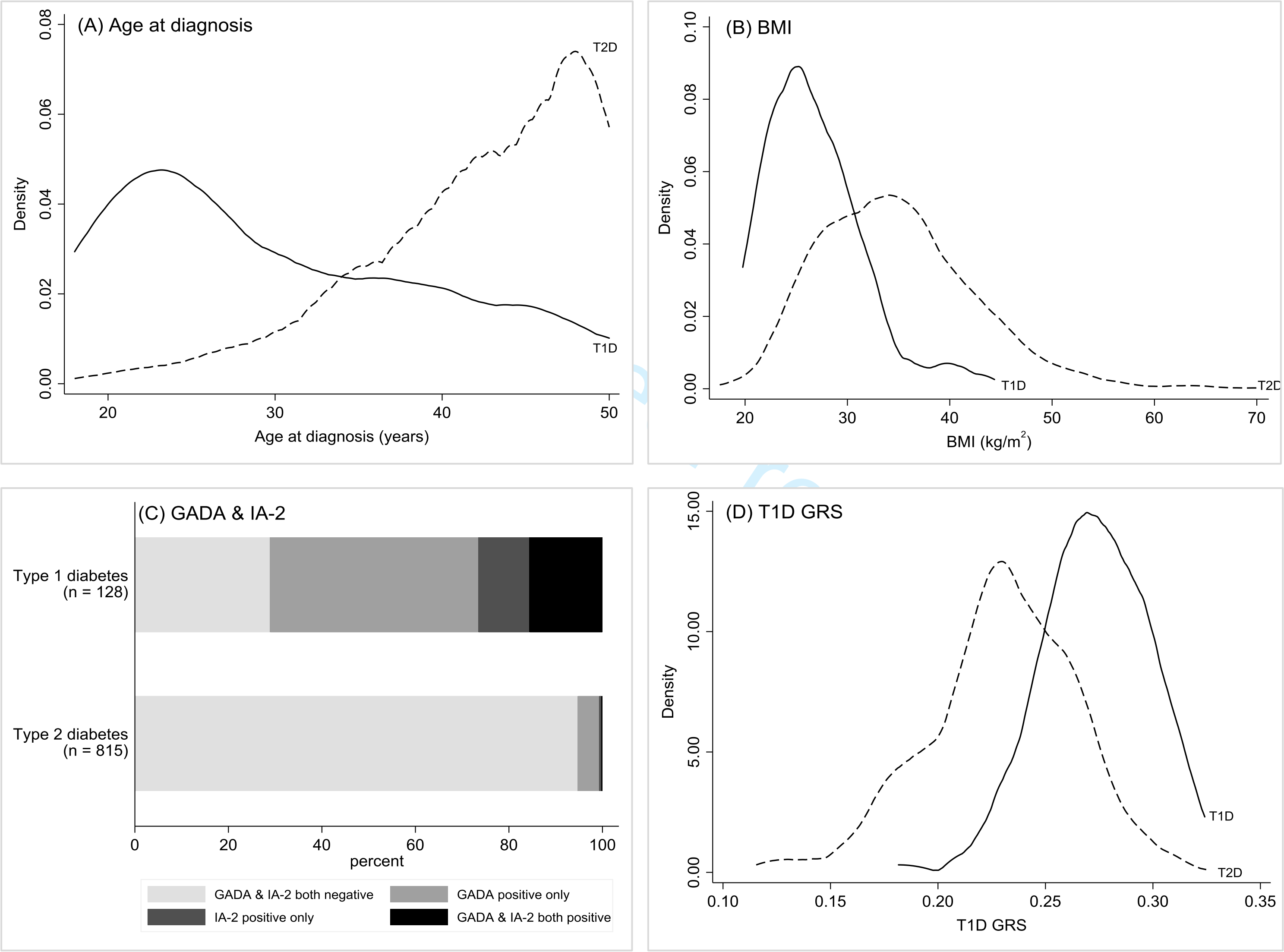
(A) Age at diagnosis
Density
Age at diagnosis (years)
T2D
T1D
(B) BMI
Density
BMI (kg/m²)
T1D
T2D
(C) GADA & IA-2
Type 1 diabetes (n = 128)
Type 2 diabetes (n = 815)
percent
GADA & IA-2 both negative
GADA positive only
IA-2 positive only
GADA & IA-2 both positive
(D) T1D GRS
Density
T1D GRS
T1D
T2D

Figure 1

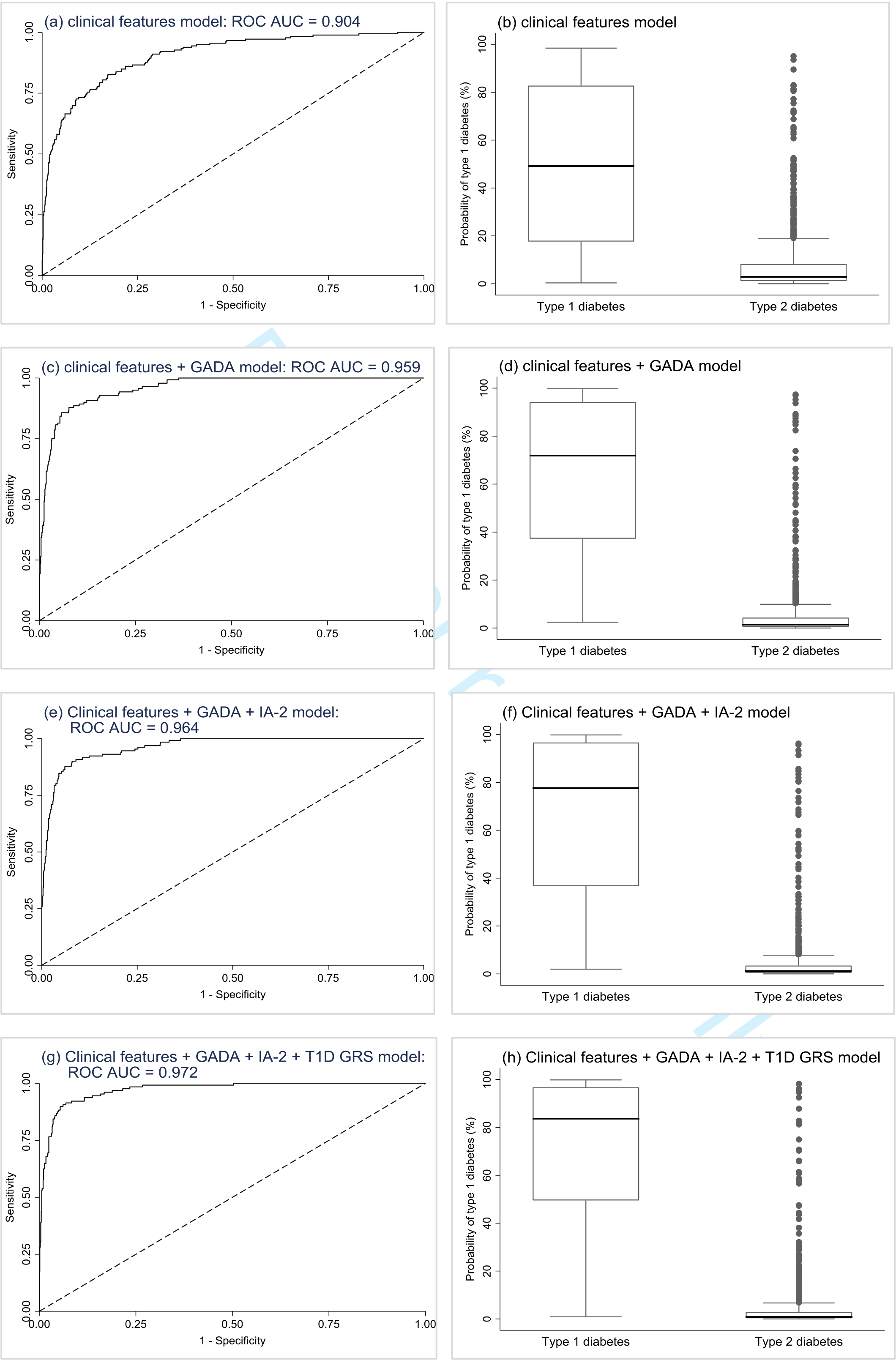
(a) clinical features model: ROC AUC = 0.904
Sensitivity
1 - Specificity
0.00
0.25
0.50
0.75
1.00
(b) clinical features model
Probability of type 1 diabetes (%)
0
20
40
60
80
100
Type 1 diabetes
Type 2 diabetes
(c) clinical features + GADA model: ROC AUC = 0.959
(d) clinical features + GADA model
(e) Clinical features + GADA + IA-2 model: ROC AUC = 0.964
(f) Clinical features + GADA + IA-2 model
(g) Clinical features + GADA + IA-2 + T1D GRS model: ROC AUC = 0.972
(h) Clinical features + GADA + IA-2 + T1D GRS model

Figure 2

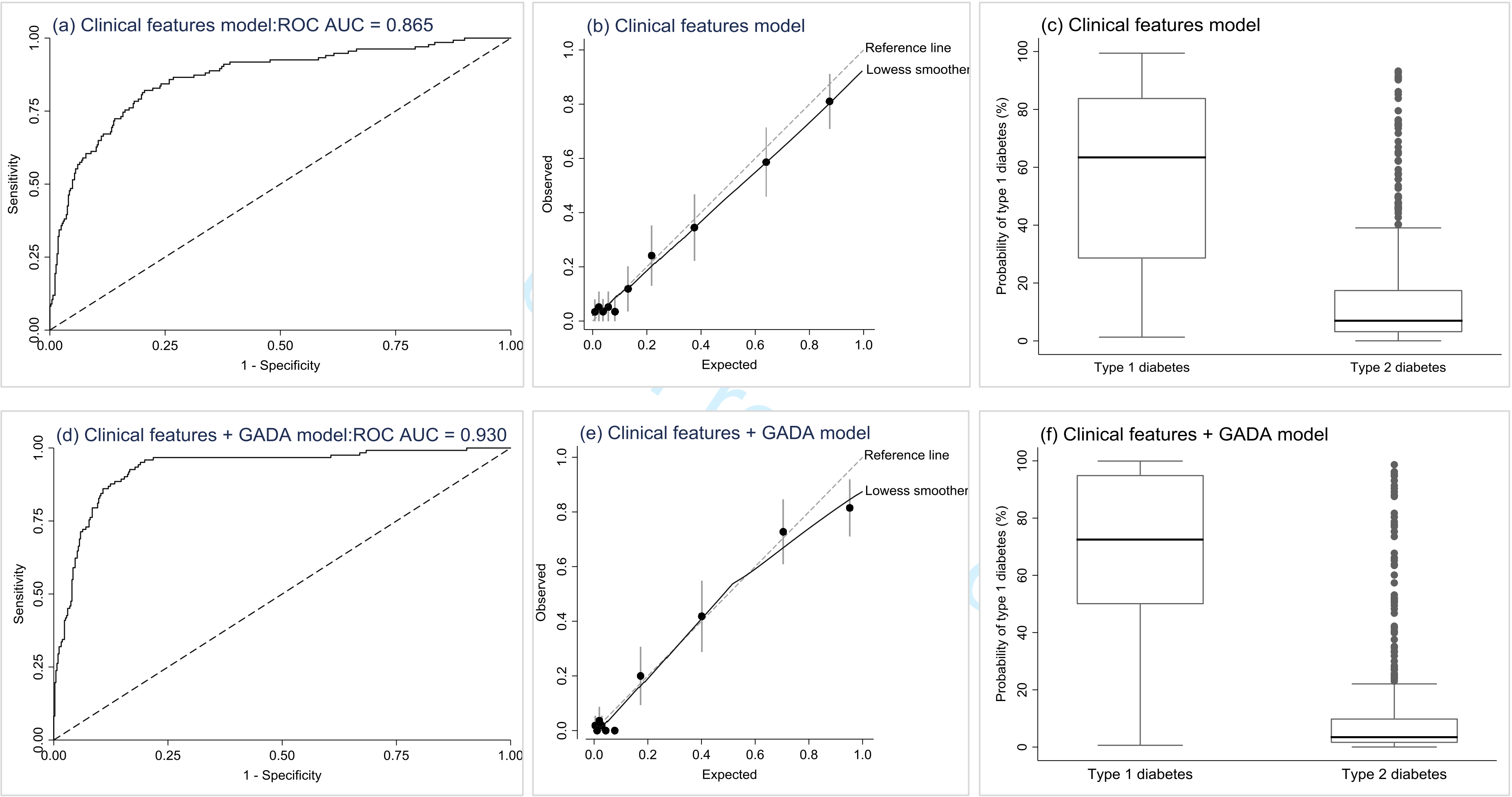
(a) Clinical features model:ROC AUC = 0.865
Sensitivity
1 - Specificity
(b) Clinical features model
Reference line
Lowess smoother
Observed
Expected
(c) Clinical features model
Probability of type 1 diabetes (%)
Type 1 diabetes
Type 2 diabetes
(d) Clinical features + GADA model:ROC AUC = 0.930
Sensitivity
1 - Specificity
(e) Clinical features + GADA model
Reference line
Lowess smoother
Observed
Expected
(f) Clinical features + GADA model
Probability of type 1 diabetes (%)
Type 1 diabetes
Type 2 diabetes

Figure 3

	DARE	PRIBA	MRC Pro/RetroMaster	MRC crossover
Included participants*	904	368	72	8
Data collection period	2007 to 2017	2011 to 2013	2013 to 2015	2013 to 2015
Study design	Cross-sectional	Longitudinal	Cross-sectional	Interventional Crossover
Setting	Primary and secondary care in eight diabetes research regions, England and retinal screening clinics.	Primary and secondary care in South West England	Primary and secondary care sites South West England, Tayside, Oxford, Glasgow, KCL and Newcastle, U.K.	Exeter and Tayside,U.K.
Inclusion criteria	Clinical diagnosis of diabetes (any type).	Clinical diagnosis of type 2 diabetes. Clinician determined requirement for DPP-IV inhibitor or GLP-1 analogue (HbA1c >7.5%)	Clinical diagnosis of type 2 diabetes non-insulin treated within 6 months of diagnosis. Participants were selected on the basis of rapid or slow progression to insulin therapy (<7, >7 years). Age 18-90 inclusive.	Clinical diagnosis of type 2 diabetes, currently treated with sulphonylurea tablets and no change in treatment in previous 3 months, Last HbA1c (within previous 12 months) ≥42 and ≤75 mmol/mol (6-9%). Age 19-79 inclusive.
Data collection	Clinical measurements and blood sample collected at visit. Ongoing biochemical data collected from pathology laboratories.	Clinical measurements and blood taken at initial visit. Follow up clinical measurements and blood collected at three and six months.	Clinical measures and fasting blood sample taken at visit.	MMT at baseline & MMT on each study drug visits. Three fasting blood collected at crossovers.

Supplementary Table 1: Cohort recruitment and data collection methods summary. *Included in the clinical features model stage 1 development.

SNP	Gene	Odds Ratio	Weight	Effect Allele
rs2187668, rs7454108	DR3/DR4	48.18	3.87	
	DR3/DR3	21.12	3.05	
	DR4/DR4	21.98	3.09	
	DR4/X	7.03	1.95	
	DR3/X	4.53	1.51	
rs1264813	HLA_A_24	1.54	0.43	T
rs2395029	HLA_B_5701	2.5	0.92	T
rs3129889	HLA_DRB1_15	14.88	2.70	A
rs2476601	PTPN22	1.96	0.67	A
rs689	INS	1.75	0.56	T
rs12722495	IL2RA	1.58	0.46	T
rs2292239	ERBB3	1.35	0.30	T
rs10509540	C10orf59	1.33	0.29	T
rs4948088	COBL	1.3	0.26	C
rs7202877		1.28	0.25	G
rs12708716	CLEC16A	1.23	0.21	A
rs3087243	CTLA4	1.22	0.20	G
rs1893217	PTPN2	1.2	0.18	G
rs11594656	IL2RA	1.19	0.17	T
rs3024505	IL10	1.19	0.17	G
rs9388489	C6orf173	1.17	0.16	G
rs1465788		1.16	0.15	C
rs1990760	IFIH1	1.16	0.15	T
rs3825932	CTSH	1.16	0.15	C
rs425105		1.16	0.15	T
rs763361	CD226	1.16	0.15	T
rs4788084	IL27	1.16	0.15	C
rs17574546		1.14	0.13	C
rs11755527	BACH2	1.13	0.12	G
rs3788013	UBASH3A	1.13	0.12	A
rs2069762	IL2	1.12	0.11	A
rs2281808		1.11	0.10	C
rs5753037		1.1	0.10	T

Supplementary Table 2: Type 1 diabetes SNPs included in the genetic risk score with weights. Effect allele is the risk increasing allele on the positive strand.

For peer review only

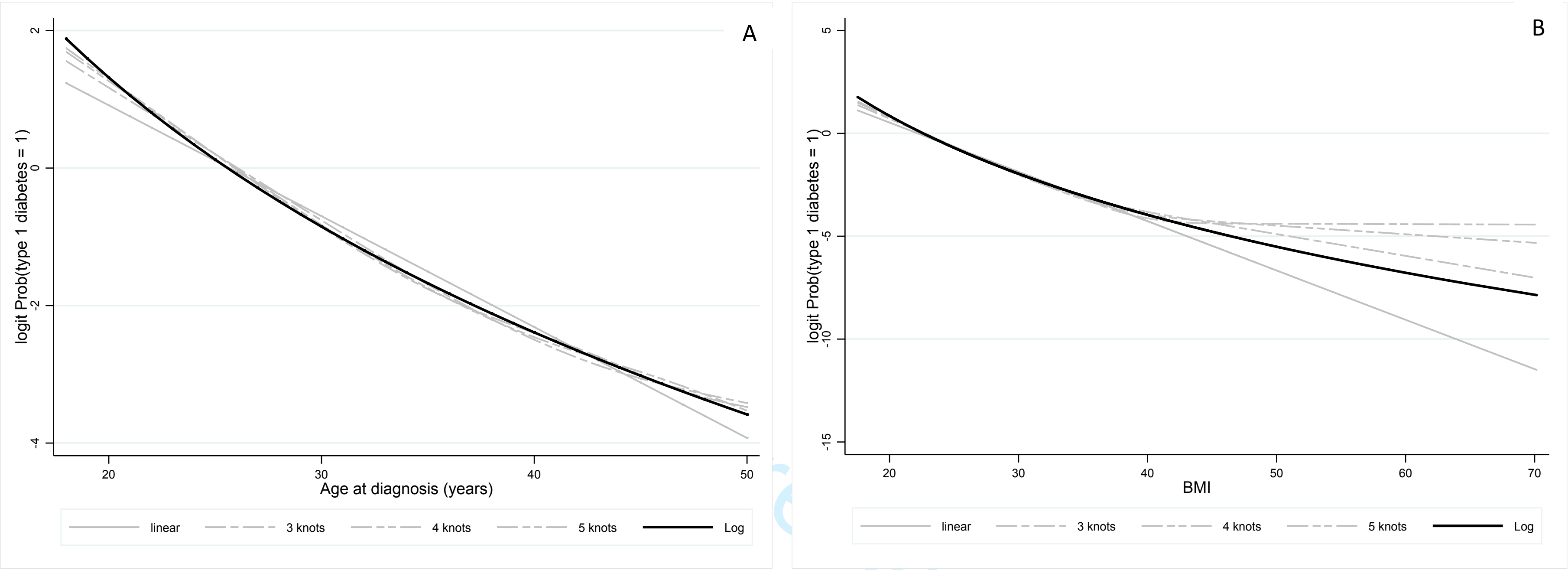

Supplementary Figure 1: Relationship between age at diagnosis (A) and BMI (B) and response modelled using restricted cubic splines (k = 3, 4 and 5) and a simple log transformation. Age at diagnosis and BMI did not predict linearly, the graphs of fitted splines and log transformation suggested that a simple log transformation was sufficient to induce linearity in both variables.

For peer review only - http://bmjopen.bmj.com/site/about/guidelines.xhtml

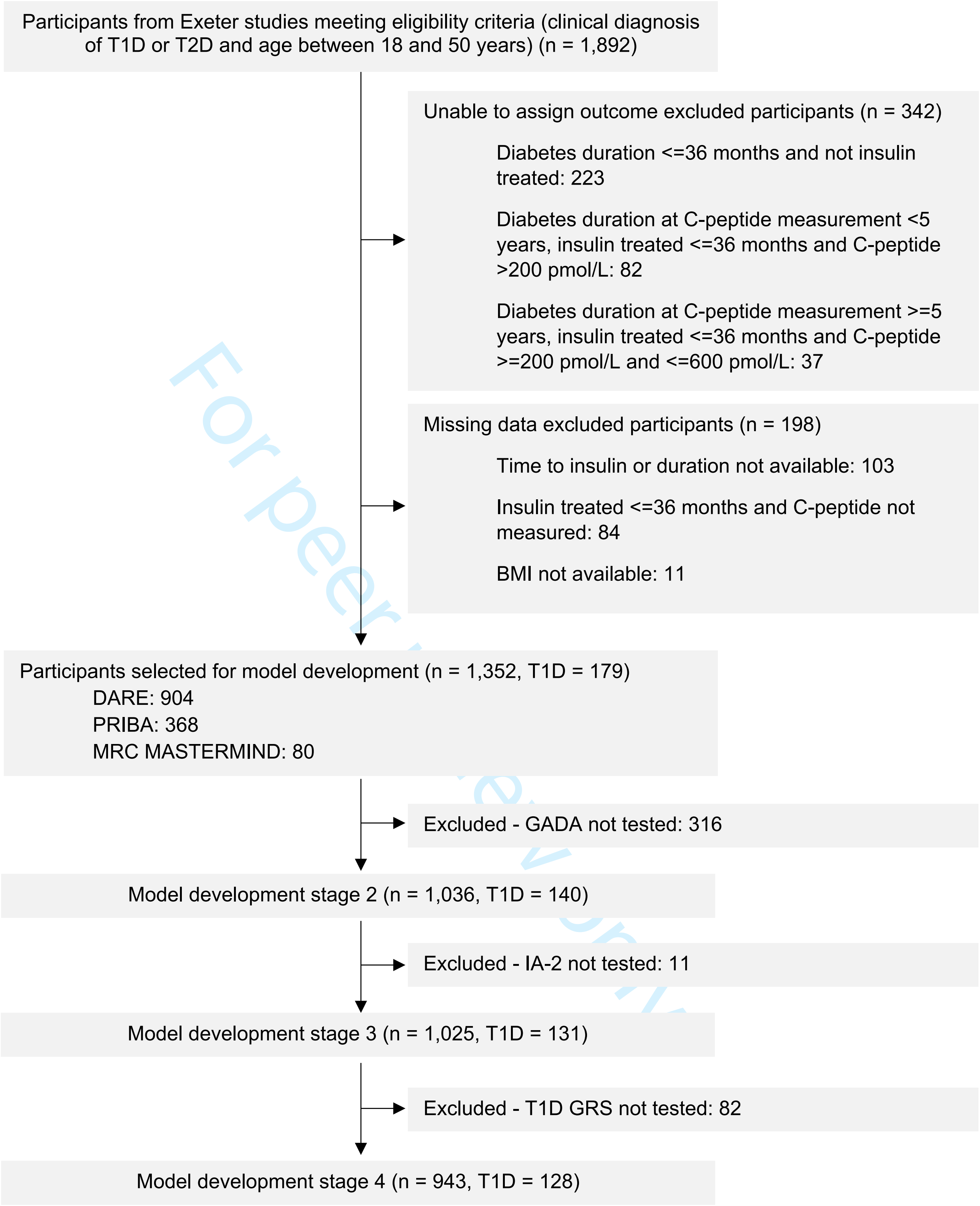

Supplementary Figure 2: Flow diagram of participants through the model development stages. T1D: type 1 diabetes, T2D: type 2 diabetes

For peer review only - http://bmjopen.bmj.com/site/about/guidelines.xhtml

	Model 1 development n = 1,352	Model 2 development n = 1,036	Model 3 development n = 1,025	Model 4 development n = 943
**Characteristic**				
Sex (% Male)	59%	59%	59%	59%
Age at diagnosis (years)*	40 [39, 41]	40 [39, 40]	40 [39, 40]	40 [39, 40]
Age at diagnosis (years) min, max	18, 50	18, 50	18, 50	18, 50
BMI (kg/m^2)*†	33 [32, 33]	33 [32, 33]	33 [32, 33]	33 [32, 33]
BMI (kg/m^2)*† min, max	17.5, 70.2	17.5, 70.2	17.5, 70.2	17.5, 70.2
Duration of diabetes (years)	13 (8, 20)	13 (8, 20)	13 (8, 20)	13 (8, 20)
Type 1 diabetes	13%	14%	13%	14%
HbA1c (%)†	8.2 (7.1, 9.6)	8.3 (7.3, 9.8)	8.3 (7.3, 9.8)	8.2 (7.2, 9.7)
HbA1c (mmol/mol)†	66 (54, 81)	67 (56, 84)	67 (56, 84)	66 (55, 83)
GADA positive (%)	-	12%	12%	12%
IA-2 positive (%)	-	-	4%	4%
T1D GRS	-	-	-	0.24 (0.22, 0.26)
T1D GRS centile	-	-	-	5.8 (1.2, 23.7)
T1D GRS min, max	-	-	-	0.12, 0.32

Supplementary Table 3: Characteristics of the Exeter, U.K. study participants included at each model development stage. Model 1 – Clinical features (Age at diagnosis & BMI), Model 2 – Clinical features + GADA, Model 3 - Clinical features + GADA + IA-2, Model 4 - Clinical features + GADA + IA-2 + T1D GRS. Median (IQR) or % or *Geometric mean [95% CI] for transformed variables. †Measured at recruitment (median 13 years post diagnosis). Minimum and maximum values for each continuous predictor variable used in the models

For peer review only - http://bmjopen.bmj.com/site/about/guidelines.xhtml

	Model 4 development n = 943	Model 4 development exclusions n = 409	p value for comparison
**Characteristic**			
Sex (% Male)	59%	60%	>0.1
Age at diagnosis (years)*	40 [39, 40]	41 [40, 42]	0.04
BMI (kg/m^2)*†	33 [32, 33]	33 [32, 33]	> 0.1
Duration of diabetes (years)	13 (8, 20)	13 (7, 20)	> 0.1
Type 1 diabetes	14%	12%	> 0.1
HbA1c (%)†	8.2 (7.2, 9.7)	8.0 (6.9, 9.3)	0.009
HbA1c (mmol/mol)†	66 (55, 83)	64 (52, 78)	0.009

Supplementary Table 4: Comparison of characteristics for participants included in the model 4 development and participants included in model 1 development but excluded from model 4. Median (IQR) or % or *Geometric mean [95% CI] for transformed variables. †Measured at recruitment (median 13 years post diagnosis).

For peer review only

Performance parameter	Development sample validation	Internal validation (bootstrap 500) Apparent (SD)	test (SD)	Optimism
**Clinical features model (n = 1,352)**				
ROC [95% CI]	0.90 [0.88, 0.93]	0.9056 (0.013)	0.9038 (0.0005)	0.0018
Calibration-in-the-large	0	0.0000 (0.000)	0.0003 (0.1072)	-0.0003
Calibration slope (b_L)	1	1.0000 (0.000)	0.9977 (0.0678)	0.0023
Brier Score	0.07 (p = 0.50)	-	-	-
Hosmer-Lemeshow	p = 0.95	-	-	-
Jack-knife cross validation†	0.09	-	-	-
**Clinical features + GADA model (n = 1,036)**				
ROC [95% CI]	0.96 [0.95, 0.97]	0.9595 (0.0070)	0.9586 (0.0010)	0.0009
Calibration-in-the-large	0	0.0000 (0.0000)	-0.0019 (0.1472)	0.0019
Calibration slope (b_L)	1	1.0000 (0.0000)	0.9850 (0.0787)	0.015
Brier Score	0.05 (p = 0.35)	-	-	-
Hosmer-Lemeshow	p = 0.39	-	-	-
Jack-knife cross validation†	0.07	-	-	-
**Clinical features + GADA + IA-2 model (n = 1,025)**				
ROC [95% CI]	0.96 [0.95, 0.98]	0.9622 (0.007)	0.9633 (0.0015)	0.0011
Calibration-in-the-large	0	0.0000 (0.000)	0.0055 (0.1567)	-0.0055
Calibration slope (b_L)	1	1.0000 (0.000)	0.9780 (0.0707)	0.022
Brier Score	0.04 (p = 0.31)	-	-	-
Hosmer-Lemeshow	p = 0.14	-	-	-
Jack-knife cross validation †	0.06	-	-	-
**Clinical features + GADA + IA-2 + T1D GRS model (n = 943)**				
ROC [95% CI]	0.97 [0.96, 0.98]	0.9718 (0.0060)	0.9710 (0.0006)	0.0008
Calibration-in-the-large	0	0.0000 (0.0000)	0.0084 (0.1675)	-0.0084
Calibration slope (b_L)	1	1.0000 (0.0000)	0.9880 (0.0810)	0.0124
Brier Score	0.04 (p = 0.35)	-	-	-
Hosmer-Lemeshow	p = 0.84	-	-	-
Jack-knife cross validation †	0.06	-	-	-

Supplementary Table 5: Model performance results for the internal validation performed at each development stage. * P value for Brier score is Spiegelhalter's z-test used to evaluate the calibration component of the Brier score, significant p-values indicate poor calibration. †Result reported as raw cross-validation estimate of prediction error with misclassification cost function (cut-off 0.5). cv.glm function in R version 3.3.3.

For peer review only - http://bmjopen.bmj.com/site/about/guidelines.xhtml

Model	LR	Adequacy
Clinical features	324.7 (df 2)	0.67
Clinical features + GADA	418.7 (df 3)	0.87
Clinical features + GADA + IA-2	447.6 (df 5)	0.93
Clinical features + GADA + IA-2 + T1D GRS	481.8 (df 6)	1.00

Supplementary Table 6: Unitless index of adequacy is the proportion of log likelihood explained by each model stage with reference to the end model containing all predictors. Based on replica models developed using stage 4 development sample (n = 943).

Model comparison	Likelihood Ratio test	Net Reclassification Improvement	Integrated Discrimination Improvement
Adding GADA to Clinical features model	LR chi2(1) = 94.02 p <0.001	0.12, p = 0.01	0.13, p < 0.001
Adding IA-2 to Clinical features + GADA model	LR chi2 (2) = 28.82 p < 0.001	0.14, p = 0.004	0.15, p < 0.001
Adding T1D GRS to Clinical features + GADA + IA-2 model	LR chi2 (2) = 34.20 p < 0.001	0.06, p = 0.04	0.06, p < 0.001

Supplementary Table 7: Model fit comparisons of nested models developed using stage 4 development sample (n = 943). Null hypothesis for Likelihood Ratio test: Additional predictor(s) has no predictive information. Net Reclassification Improvement calculated using 50% classification cut-off.

Model	Clinical features ROC AUC	Clinical features + GADA ROC AUC	Clinical features + GADA + IA-2 ROC AUC
Development sample 1 (n = 1,352)	0.90 [0.88, 0.93]	-	-
Development sample 2 (n = 1,036)	-	0.96 [0.95, 0.97]	-
Development sample 3 (n = 1,025)	-	-	0.96 [0.95, 0.98]
**Development sample 4 (n = 943)**	**0.91 [0.89, 0.94]**	**0.96 [0.94, 0.97]**	**0.96 [0.95, 0.98]**

Supplementary Table 8: Model performance comparison with replica models developed using stage 4 development sample (n = 943).

For peer review only - http://bmjopen.bmj.com/site/about/guidelines.xhtml

Model	ROC AUC [95% CI]	n
Clinical Features	0.72 [0.61, 0.83]	104
Clinical Features + GADA	0.89 [0.80, 0.98]	78
Clinical Features + GADA + IA2	0.89 [0.80, 0.98]	77
Clinical Features + GADA + IA2 + T1D GRS	0.95 [0.90, 1.00]	71

Supplementary Table 9: ROC AUC calculated including only patients aged 25-35 years (inclusive) at diagnosis and with BMI 25-35 kg/m^2 (inclusive).

For peer review only

For peer review only - http://bmjopen.bmj.com/site/about/guidelines.xhtml

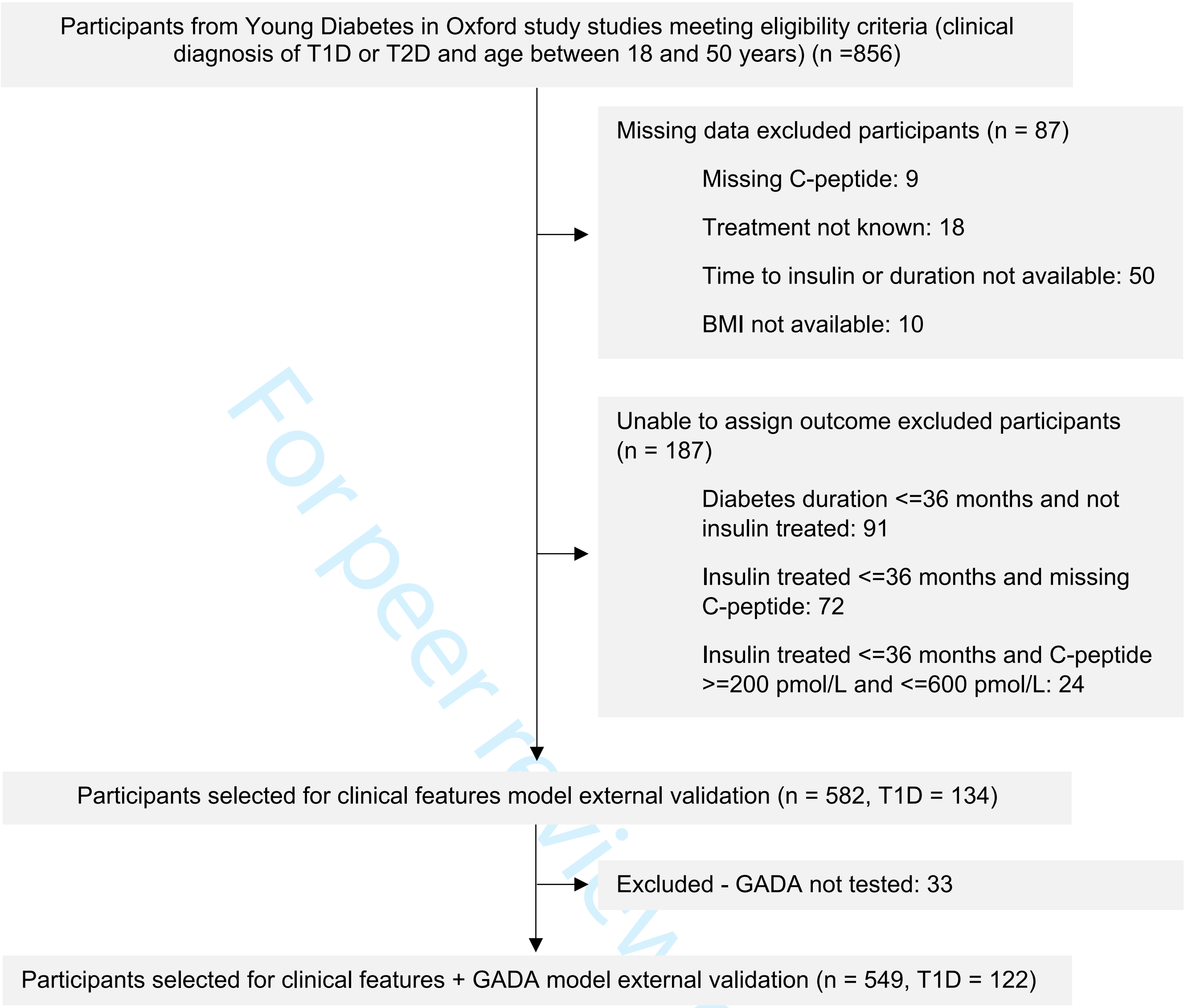

Supplementary Figure 3: Flow diagram of participants through the model external validation stages. T1D: type 1 diabetes, T2D: type 2 diabetes

For peer review only

For peer review only - http://bmjopen.bmj.com/site/about/guidelines.xhtml

	Model 1 development n = 1,352	Model 1 validation n = 582	comparison p value	Model 2 development n = 1,036	Model 2 validation n = 549	comparison p value
**Characteristic**						
Sex (% Male)	59%	61%	>0.1	59%	61%	> 0.1
Age at diagnosis (years)	43 (36, 48)	37 (30, 41)	<0.001	43 (36, 48)	37 (30, 41)	< 0.001
BMI (kg/m^2)*	33 (28, 38)	31 (27, 36)	<0.001	33 (28, 38)	31 (27, 36)	< 0.001
Duration of diabetes (years)*	13 (8, 20)	14 (8, 23)	0.03	13 (8, 20)	13 (8, 23)	> 0.1
Type 1 diabetes	13%	23%	<0.001	14%	22%	< 0.001
HbA1c (%)*	8.2 (7.1, 9.6)	8.1 (7.2, 9.3)	>0.1	8.3 (7.3, 9.8)	8.1 (7.2, 9.4)	0.08
HbA1c (mmol/mol)*	66 (54, 81)	65 (55, 78)	>0.1	67 (56, 84)	65 (55, 79)	0.08
GADA (% positive)	-	-	-	12%	20%	< 0.001

Supplementary Table 10: Baseline characteristics comparison of the development and validation data sets for: Model 1 – Clinical features (Age at diagnosis & BMI) and Model 2 – Clinical features + GADA. *Measured at recruitment (median 13 years and 14 years post diagnosis in development data sets and validation data sets). Kruskal-Wallis used for comparison testing continuous variables, chi-square for categorical variables.

For peer review only

For peer review only - http://bmjopen.bmj.com/site/about/guidelines.xhtml

Performance parameter	External validation
**Clinical features model (n = 582)**	
ROC [95% CI]	0.86 [0.83, 0.90]
Expected/Observed	1.06
Calibration-in-the-large ($a|b_L$=1)	-0.14
Calibration slope (b_L)	0.85
Overall misclassification	-0.14 p = 0.05
Brier Score*	0.11 (p = 0.14)
**Clinical features + GADA model (n = 549)**	
ROC [95% CI]	0.93 [0.90, 0.96]
Expected/Observed	1.08
Calibration-in-the-large ($a|b_L$=1)	-0.23
Calibration slope (b_L)	0.90
Overall misclassification	-0.10 p > 0.1
Brier Score*	0.08 (p = 0.29)

Supplementary Table 11: Model performance results for the external validation of the clinical features and clinical features+ GADA models. * P value for Brier score is Spiegelhalter's z-test used to evaluate the calibration component of the Brier score, significant p-values indicate poor calibration.

For peer review only

Clinical features	Development (n = 1,352)					Validation (n = 582)				
	Probability cut-off for classifying type 1 diabetes					Probability cut-off for classifying type 1diabletes				
	10	30	50	70	90	10	30	50	70	90
Sensitivity/specificity (%)	85/79	64/95	49/98	35/99	15/100	91/62	73/85	59/93	45/96	13/99
Accuracy (%)	80	90	91	90	89	69	82	85	84	79
Positive predictive value (PPV) (%)	38	64	79	83	90	42	59	71	77	77
Negative predictive value (NPV) (%)	97	95	93	91	89	96	91	88	85	79
Clinical features + GADA	Development (n = 1,036)					Validation (n = 549)				
	Probability cut-off for classifying type 1 diabetes					Probability cut-off for classifying type 1diabletes				
	10	30	50	70	90	10	30	50	70	90
Sensitivity/specificity (%)	90/88	80/96	66/97	52/99	31/100	97/75	86/89	75/93	55/96	42/97
Accuracy (%)	89	94	93	92	90	80	88	88	87	85
Positive predictive value (PPV) (%)	55	75	80	85	92	53	69	73	80	81
Negative predictive value (NPV) (%)	98	97	95	93	90	99	96	93	88	85

Supplementary Table 12: Classification table comparing the development and validation samples at different cut-offs for probability of type 1 diabetes using the clinical features and clinical features + GADA logistic regression models.
Accuracy = (true positives + true negatives)/total number of participants.
Positive predictive value (PPV) = (sensitivity × prevalence)/[(sensitivity × prevalence) + ([1 – specificity] × [1−prevalence])].
Negative predictive value (NPV) = [specificity × (1 − prevalence)]/[(specificity × [1 − prevalence]) + ([1 − sensitivity] × prevalence)].
PPV and NPV assume prevalence for type 1 diabetes: Clinical features model – 13% (development) and 23% (validation), Clinical features + GADA model - 14% (development) and 22% (validation).

For peer review only - http://bmjopen.bmj.com/site/about/guidelines.xhtml

Age at diagnosis (years)	BMI (kg/m²)	GADA positive	C-Peptide (PmolL)*	Insulin Treated	Time to insulin (months)	Duration at screening (years)†	Actual diabetes outcome	Probability of type 1 diabetes‡ (%)
18	26	0	775	1	Immediate	15	Type 2 diabetes	80
21	23	0	868	1	Immediate	10	Type 2 diabetes	82
27	29	1	-	0	-	3	Type 2 diabetes	88
38	22	1	550	1	48	10	Type 2 diabetes	88
36	22	1	175	1	72	12	Type 2 diabetes	89
23	32	1	25	1	48	29	Type 2 diabetes	90
30	25	1	25	1	36	30	Type 2 diabetes	91
29	25	1	225	1	48	12	Type 2 diabetes	93
23	28	1	50	1	120	28	Type 2 diabetes	95
33	21	1	65	1	96	47	Type 2 diabetes	95
34	20	1	25	1	120	22	Type 2 diabetes	96
23	22	1	-	0	-	3	Type 2 diabetes	99

Supplementary table 13: Characteristics of participants with probability of Type 1 diabetes > 80% but with type 2 diabetes actual outcome *Non fasting equivalent, measured > 5 years post diagnosis (unless < 200 PmolL prior to 5 years). † C-peptide measured at single screening visit. ‡Clinical features + GADA model applied to participants in the YDX study.

For peer review only

For peer review only - http://bmjopen.bmj.com/site/about/guidelines.xhtml

Age at diagnosis (years)	BMI (kg/m^2)	GADA positive	C-Peptide (PmolL)*	Insulin Treated	Time to insulin (months)	Duration at screening (years)†	Actual diabetes outcome	Probability of type 1 diabetes (%)‡
41	40	0	50	1	12	41	Type 1 diabetes	0.6
40	34	0	198	1	12	34	Type 1 diabetes	1.8
43	31	0	125	1	3	1	Type 1 diabetes	2.1
39	33	0	25	1	24	17	Type 1 diabetes	2.5
38	25	0	68	1	Immediate	19	Type 1 diabetes	12.7
39	40	1	50	1	Immediate	16	Type 1 diabetes	14.9

Supplementary table 14: Characteristics of participants with probability of Type 1 diabetes < 16% (Youden's Index cut-off) but with type 1 diabetes actual outcome *Non-fasting equivalent, measured > 5 years post diagnosis (unless < 200 pmolL prior to 5 years). † C-peptide measured at single screening visit. ‡Clinical features + GADA model applied to participants in the YDX study.

Model	ROC [95% CI]	Jack-knife cross validation *
Clinical features + IA-2	0.93 [0.90, 0.95]	0.07
Clinical features + T1D GRS	0.93 [0.90, 0.95]	0.08
Clinical features + IA-2 + T1D GRS	0.95 [0.93, 0.97]	0.06
Clinical features + GADA + T1D GRS	0.97 [0.96, 0.98]	0.07

Supplementary table 15: Model performance results for the four additional models in the online calculator. * Result reported as raw cross-validation estimate of prediction error with misclassification cost function (cut-off 0.5). cv.glm function in R version 3.3.3.

For peer review only - http://bmjopen.bmj.com/site/about/guidelines.xhtml

For peer review only

Included	β (SE)	Odds Ratio [95% CI]	p value
Constant (intercept)	37.94 (2.67)	-	-
Age at diagnosis (years) *	-5.09 (0.41)	0.006 [0.003, 0.014]	<0.001
BMI (kg/m^2) *	-6.34 (0. 60)	0.002 [0.001, 0.005]	<0.001

Supplementary Table 16: Clinical features logistic regression model (model 1). * Log transformed. Linear Predictor mean -2.96, sd 1.98

Included	β (SE)	Odds Ratio [95% CI]	p value
Constant (intercept)	-0.98 (0.19)	-	-
Model 1 linear predictor	0.94 (0.08)	2.57 (2.18, 3.03)	< 0.001
GADA positive	3.11 (0.32)	22.50 (12.13, 41.76)	< 0.001

Supplementary Table 17: Clinical features + GADA logistic regression model (model 2). Linear Predictor mean -3.37, sd 2.53

For peer review only

For peer review only - http://bmjopen.bmj.com/site/about/guidelines.xhtml

Included	β (SE)	Odds Ratio [95% CI]	p value
Constant (intercept)	-1.28 (0.21)	-	
Model 1 linear predictor	0.92 (0.09)	2.50 [2.10, 2.98]	< 0.001
Antibody status - GADA positive only	3.08 (0.35)	21.81 [11.06, 43.02]	< 0.001
Antibody status - IA-2 positive only	3.49 (0.78)	32.93 [7.11, 152.64]	< 0.001
Antibody status - GADA & IA-2 both positive	4.35 (0.75)	77.53 [17.74, 338.84]	< 0.001

Supplementary Table 18: Clinical features + GADA + IA-2 logistic regression model (model 3). Linear Predictor mean -3.55, sd 2.58

Included	β (SE)	Odds Ratio [95% CI]	p value
Constant (intercept)	-0.67 (0.24)	-	-
Model 3 linear predictor	0.88 (0.08)	2.40 [2.06, 2.80]	< 0.001
T1D GRS (per 1 SD change)	1.08 (0.21)	2.93 [1.96, 4.39]	< 0.001

Supplementary Table 19: Clinical features + GADA + IA-2 + T1D GRS logistic regression model (model 4). T1D GRS standardized using mean 0.2356997, sd 0.0363499. Linear Predictor mean -3.74, sd 2.89.

Included	β (SE)	Odds Ratio [95% CI]	p value
Constant (intercept)	-0.36 (0.17)	-	-
Model 1 linear predictor	0.99 (0.08)	2.70 [2.30, 3.16]	< 0.001
IA-2 positive	3.19 (0.55)	24.39 [8.27, 71.92]	< 0.001

Supplementary Table 20: Clinical features + IA-2 logistic regression model. Linear Predictor mean -3.17, SD 2.28

For peer review only - http://bmjopen.bmj.com/site/about/guidelines.xhtml

Included	β (SE)	Odds Ratio [95% CI]	p value
Constant (intercept)	-0.65 (0.18)	-	-
Model 1 linear predictor	0.87 (0.07)	2.39 [2.09, 2.74]	< 0.001
T1D GRS (per 1 SD change)	1.22 (0.15)	3.38 [2.51, 4.54]	< 0.001

Supplementary Table 21: Clinical features + T1D GRS logistic regression model. T1D GRS standardized using mean 0.2360879, sd 0.0358468. Linear Predictor mean -3.180108, sd 2.401089.

Included	β (SE)	Odds Ratio [95% CI]	p value
Constant (intercept)	-1.12 (0.23)	-	-
Model 1 linear predictor	0.87 (0.09)	2.40 [2.02, 2.84]	< 0.001
T1D GRS (per 1 SD change)	1.36 (0.20)	3.89 [2.64, 5.74]	< 0.001
IA-2 positive	2.95 (0.65)	19.17 [5.33, 68.81]	< 0.001

Supplementary Table 22: Clinical features + IA-2 + T1D GRS logistic regression model. T1D GRS standardized using mean 0.235673, sd 0.0363399. Linear Predictor mean -3.537275, sd 2.79395.

Included	β (SE)	Odds Ratio [95% CI]	p value
Constant (intercept)	-1.50 (0.24)	-	-
Model 1 linear predictor	0.85 (0.09)	2.33 [1.97, 2.76]	< 0.001
T1D GRS (per 1 SD change)	1.12 (0.20)	3.05 [2.09, 4.46]	< 0.001
GADA positive	2.63 (0.34)	13.89 [7.17, 26.90]	< 0.001

Supplementary Table 23: Clinical features + GADA + T1D GRS logistic regression model. T1D GRS standardized using mean 0.2359649, sd 0.0363407. Linear Predictor mean - 3.596086, sd 2.868552.

For peer review only - http://bmjopen.bmj.com/site/about/guidelines.xhtml

Model	Linear predictor (lp) regression equation*
Clinical features	37.94 + (-5.09 * log(age)) + (-6.34 * log(BMI))
Clinical features + GADA†	34.8057844720 + (-4.801441792 * log (Age)) + (-5.980577792 * log(BMI)) + (2.937107976 * GADA†)
Clinical features + GADA + IA-2	33.49649577 + (-4.665598345 * Log(Age)) + (-5.81137397 * Log(BMI)) + (3.082366 * AntiStatus1‡) + (3.494462 * AntiStatus2‡) + (4.350717 * AntiStatus3‡)
Clinical features + GADA + IA-2 + T1D GRS	21.57649882 + (-4.086215772 * Log(Age)) + (-5.096252172 * Log(BMI)) + (2.702010666 * AntiStatus1‡) + (3.063255174 * AntiStatus2‡) + (3.813850704 * AntiStatus3‡) + (30.11052 * T1D GRS)
Clinical features + IA-2	37.26905033 + (3.194096 * IA-2†) + (-5.047657308 * Log(Age)) + (-6.287258808 * Log(BMI))
Clinical features + T1D GRS	24.46138054 + (-4.443506884 * Log(Age)) + (-5.534741384 *Log(BMI)) + (33.93968 * T1D GRS)
Clinical features + IA-2 + T1D GRS	23.2151829 +(2.953142 * IA-2†) + (-4.446784844 *Log(Age))+(-5.538824344 * Log(BMI)) + (37.40205 * T1D GRS)
Clinical features + GADA + T1D GRS	23.20924904 + (2.63093 * GADA†) + (-4.303557843 * Log(Age)) + (-5.360423718 *Log(BMI)) + (31.22606 * T1D GRS)

Supplementary Table 24: *To convert to probability use exp(lp)/(1+exp(lp)). †Dummy variable: negative = 0, positive = 1 ‡Dummy variables: false = 0, true = 1, AntiStatus1 = GADA positive only, AntiStatus2 = IA-2 positive only, AntiStatus3 = Both GADA and IA-2 positive.

For peer review only - http://bmjopen.bmj.com/site/about/guidelines.xhtml

TRIPOD Checklist: Prediction Model Development and Validation

Section/Topic	Item		Checklist Item	Page
**Title and abstract**				
Title	1	D;V	Identify the study as developing and/or validating a multivariable prediction model, the target population, and the outcome to be predicted.	1
Abstract	2	D;V	Provide a summary of objectives, study design, setting, participants, sample size, predictors, outcome, statistical analysis, results, and conclusions.	4,5
**Introduction**				
Background and objectives	3a	D;V	Explain the medical context (including whether diagnostic or prognostic) and rationale for developing or validating the multivariable prediction model, including references to existing models.	7
	3b	D;V	Specify the objectives, including whether the study describes the development or validation of the model or both.	9
**Methods**				
Source of data	4a	D;V	Describe the study design or source of data (e.g., randomized trial, cohort, or registry data), separately for the development and validation data sets, if applicable.	10 ,
	4b	D;V	Specify the key study dates, including start of accrual; end of accrual; and, if applicable, end of follow-up.	S.T.1
Participants	5a	D;V	Specify key elements of the study setting (e.g., primary care, secondary care, general population) including number and location of centres.	S.T.1
	5b	D;V	Describe eligibility criteria for participants.	10, SF2
	5c	D;V	Give details of treatments received, if relevant.	NA
Outcome	6a	D;V	Clearly define the outcome that is predicted by the prediction model, including how and when assessed.	11
	6b	D;V	Report any actions to blind assessment of the outcome to be predicted.	NA
Predictors	7a	D;V	Clearly define all predictors used in developing or validating the multivariable prediction model, including how and when they were measured.	11-13
	7b	D;V	Report any actions to blind assessment of predictors for the outcome and other predictors.	NA
Sample size	8	D;V	Explain how the study size was arrived at.	14, S.F.2
Missing data	9	D;V	Describe how missing data were handled (e.g., complete-case analysis, single imputation, multiple imputation) with details of any imputation method.	13,14
Statistical analysis methods	10a	D	Describe how predictors were handled in the analyses.	13,14
	10b	D	Specify type of model, all model-building procedures (including any predictor selection), and method for internal validation.	13,14
	10c	V	For validation, describe how the predictions were calculated.	13,14
	10d	D;V	Specify all measures used to assess model performance and, if relevant, to compare multiple models.	13,14
	10e	V	Describe any model updating (e.g., recalibration) arising from the validation, if done.	NA
Risk groups	11	D;V	Provide details on how risk groups were created, if done.	NA
Development vs. validation	12	V	For validation, identify any differences from the development data in setting, eligibility criteria, outcome, and predictors.	18
**Results**				
Participants	13a	D;V	Describe the flow of participants through the study, including the number of participants with and without the outcome and, if applicable, a summary of the follow-up time. A diagram may be helpful.	S.F.2
	13b	D;V	Describe the characteristics of the participants (basic demographics, clinical features, available predictors), including the number of participants with missing data for predictors and outcome.	S.F.2 S.T.3
	13c	V	For validation, show a comparison with the development data of the distribution of important variables (demographics, predictors and outcome).	S.T.9
Model development	14a	D	Specify the number of participants and outcome events in each analysis.	S.F.2
	14b	D	If done, report the unadjusted association between each candidate predictor and outcome.	17
Model specification	15a	D	Present the full prediction model to allow predictions for individuals (i.e., all regression coefficients, and model intercept or baseline survival at a given time point).	S.T.15 - 22
	15b	D	Explain how to the use the prediction model.	S.T. 23
Model performance	16	D;V	Report performance measures (with CIs) for the prediction model.	S.T.5
Model-updating	17	V	If done, report the results from any model updating (i.e., model specification, model performance).	NA
**Discussion**				
Limitations	18	D;V	Discuss any limitations of the study (such as nonrepresentative sample, few events per predictor, missing data).	21
Interpretation	19a	V	For validation, discuss the results with reference to performance in the development data, and any other validation data.	20
	19b	D;V	Give an overall interpretation of the results, considering objectives, limitations, results from similar studies, and other relevant evidence.	20-22
Implications	20	D;V	Discuss the potential clinical use of the model and implications for future research.	22
**Other information**				
Supplementary information	21	D;V	Provide information about the availability of supplementary resources, such as study protocol, Web calculator, and data sets.	19
Funding	22	D;V	Give the source of funding and the role of the funders for the present study.	23, 24

For peer review only

TRIPOD Checklist: Prediction Model Development and Validation

*Items relevant only to the development of a prediction model are denoted by D, items relating solely to a validation of a prediction model are denoted by V, and items relating to both are denoted D;V. We recommend using the TRIPOD Checklist in conjunction with the TRIPOD Explanation and Elaboration document.

Abbreviations:

NA = not applicable

S.T = Supplementary table

S.F = Supplementary Figure

For peer review only
